# Diagnosis, Prognosis, and Drug Target Discovery for Chronic Widespread Pain: A Large Proteogenomic Study

**DOI:** 10.1002/advs.202507691

**Published:** 2025-09-30

**Authors:** Li Chen, Eoin Kelleher, Ruogu Meng, Duanke Liu, Yuchen Guo, Yunhe Wang, Yaqing Gao, Zhe Huang, Zhu Liang, Shuai Yuan, Chao Zeng, Jun Ma, Yanhui Dong, Anushka Irani, Guanghua Lei, Junqing Xie, Daniel Prieto‐Alhambra

**Affiliations:** ^1^ Centre for Statistics in Medicine and NIHR Biomedical Research Centre Oxford NDORMS University of Oxford Oxford OX3 7LD UK; ^2^ Institute of Child and Adolescent Health School of Public Health Peking University Beijing 100191 China; ^3^ Nuffield Department of Clinical Neurosciences University of Oxford Oxford OX3 9DU UK; ^4^ National Institute of Health Data Science Peking University Beijing 100191 China; ^5^ Beijing Institute of Ophthalmology Beijing Tongren Eye Center Beijing Key Laboratory of Ophthalmology and Visual Sciences Beijing Tongren Hospital, Capital Medical University Beijing 100730 China; ^6^ Nuffield Department of Population Health University of Oxford Oxford OX3 7LF UK; ^7^ Nuffield Department of Medicine Target Discovery Institute Center for Medicines Discovery University of Oxford Oxford OX3 7FZ UK; ^8^ Department of Surgery Perelman School of Medicine at the University of Pennsylvania PA 19104 USA; ^9^ Institute of Environmental Medicine Karolinska Institutet Stockholm 171 77 Sweden; ^10^ Department of Orthopaedics Xiangya Hospital, Central South University Changsha 410013 China; ^11^ Division of Rheumatology Mayo Clinic Florida Florida 32224 USA; ^12^ The Queen's College University of Oxford Oxford OX1 4AW UK; ^13^ Department of Medical Informatics Erasmus University Medical Center Rotterdam 3015 GD The Netherlands

**Keywords:** biomarker, drug‐repurposing, nociplastic pain, proteomic, wide spread pain

## Abstract

Chronic widespread pain (CWP) remains challenging due to its heterogeneous causes and complex mechanisms. A total of 2920 plasma proteins are analyzed from 29,254 UK Biobank participants. A total of 256 proteins are identified as cross‐sectionally correlated with CWP. A simple (top 10 proteins) and comprehensive (all significant proteins) proteomic‐based score (ProtS) is created for CWP diagnosis, both outperforming and improving the existing clinical score (area under the curve, AUC: 0.801, 0.723, and 0.791 alone, and 0.856 and 0.880 in combination). In addition, the protein score predicted 13‐years risk of pain‐related traits over the body, including pain onset, progression, and intensity; Moreover, it has stronger associations with nociplastic pain and fibromyalgia compared to nociceptive and neuropathic pain, implying a unique protein signature of different pain mechanisms. Finally, among 434 candidate proteins prioritized in the observational analysis, 18 are corroborated with causal relevance by Mendelian randomization, and importantly, four (CA14, DPEP1, LGALS3, and TNF) showed potential as novel drug targets repurposed for treating CWP.

## Graphical abstract

Plasma proteomics is leveraged to decode the biological underpinnings of chronic widespread pain. A nested machine learning framework integrates proteomic signatures, prospective outcomes, and Mendelian randomization to uncover 18 causal proteins. Several targets are druggable, including CA14 and TNF, highlighting new avenues for precision pain therapeutics and drug repurposing in nociplastic pain.

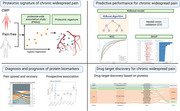

## 1 Introduction

Chronic pain affects 27.5% of the global population and is one of the leading causes of medical consultation and disability,^[^ ^1^, ^2^ ^]^ particularly among adults over 65, where joint, back, and neck pain are common.^[^ ^3^, ^4^ ^]^ The burden of chronic pain extends beyond physical suffering, as it poses significant societal costs through lost productivity and increased healthcare resource utilization, presenting a major global health challenge.^[^ ^5^, ^6^ ^]^

The International Association for the Study of Pain (IASP) defines pain as an unpleasant sensory and emotional experience. While acute pain serves a protective function by signaling potential or actual tissue damage, chronic pain is defined as pain persisting for more than three months, continuing beyond the normal healing period. Unlike acute pain, which is adaptive, chronic pain is often maladaptive as it no longer serves a protective role. Indeed, chronic pain is increasingly recognized as a distingct condition, with the 2019 ICD‐11 classification introducing “chronic primary pain”, where pain is the principal problem rather than a result of another disease.^[^ ^7^, ^8^, ^9^, ^10^ ^]^ Chronic pain was traditionally classified as either neuropathic pain, which originates from a lesion or disease of the somatosensory nervous system, or nociceptive pain, which results from actual or threatened non‐neural tissue damage.^[^ ^8^, ^9^ ^]^ However, in 2016, a third category—nociplastic pain—was introduced to describe pain arising from altered nociception without clear evidence of tissue or nerve damage. Nociplastic pain, characterized by its widespread and diffuse nature, is particularly debilitating and is best typified by conditions such as fibromyalgia.^[^ ^11^ ^]^

Despite advances in understanding these pain mechanisms, clinical tools to accurately diagnose and distinguish between them remain lacking. Existing clinical models do not account for the molecular underpinnings that could differentiate nociceptive, neuropathic, and nociplastic pain. Here, proteomics – the large‐scale study of proteins – offers a promising avenue. By examining proteins as biomarkers, proteomics offers promising opportunities to improve diagnostic accuracy and support personalized therapeutic strategies for chronic pain.

The field of proteomics has significantly advanced our understanding of molecular mechanisms in complex diseases like cardiovascular disease, diabetes, and cancer by integrating genetic, lifestyle, and environmental influences.^[^ ^12^ ^]^ Given the recognition of pain vulnerability—where genetic and biological factors predispose certain individuals to developing chronic pain^[^ ^13^, ^14^, ^15^ ^]^—proteomic analyses could help identify those at higher risk, facilitating early recognition and intervention. Prior biomarker studies in chronic pain have been limited by small sample sizes and a narrow focus on specific proteins, failing to capture the full complexity of pain mechanisms.^[^ ^16^ ^]^ By leveraging a comprehensive proteomic dataset in a large population‐based study, our research addresses this gap and provides an opportunity to identify novel biomarkers associated with pain vulnerability and distinct pain mechanisms.^[^ ^17^ ^]^ These innovative approaches may offer a deeper understanding of chronic pain mechanisms, opening pathways to improved diagnostic assays and novel therapeutic targets.

We therefore aimed to: (1) evaluate the proteomic signatures distinguishing acute and chronic pain, identifying unique molecular patterns; (2) assess the diagnostic and prognostic validity of models based on 2923 unique plasma proteins in predicting widespread chronic pain, comparing their performance to conventional clinical risk models, and to explore the added value of integrating plasma proteins into clinical risk models; and (3) to assess the causal relevance of key identified proteins and their potential as therapeutic targets for the treatment of chronic pain.

## 2 Results

### 2.1 Baseline Characteristics and Pain Phenotypes

We analyzed 29254 participants with plasma proteomics data (Olink) from the UK Biobank, of whom 44.6% were male, with a median age of 55.8 years (Table S2, Supporting Information). The comparison of participants with and without proteins is provided in Table S1 (Supporting Information). In the present study, 52.3% of participants reported suffering from chronic pain at baseline. Regarding pain types, 85.2% reported musculoskeletal pain, 22.5% pain in the head and face, 10.7% abdominal pain, and 3.0% experienced widespread pain. Overall, 24.4% of participants reported at least two pain sites affected. A Venn diagram depicting the overlap between different chronic pain types, including widespread chronic pain, is provided in Figure S5 (Supporting Information). In 2019, data on pain mechanisms were collected from a total of 8225 participants. Among them, 571 reported nociceptive pain, 75 reported neuropathic pain, 4153 experienced nociplastic pain, and 717 had a diagnosis of fibromyalgia (Table S2, Supporting Information).

### 2.2 Proteomic Associations with Chronic and Acute Pain

**Figure 1:**
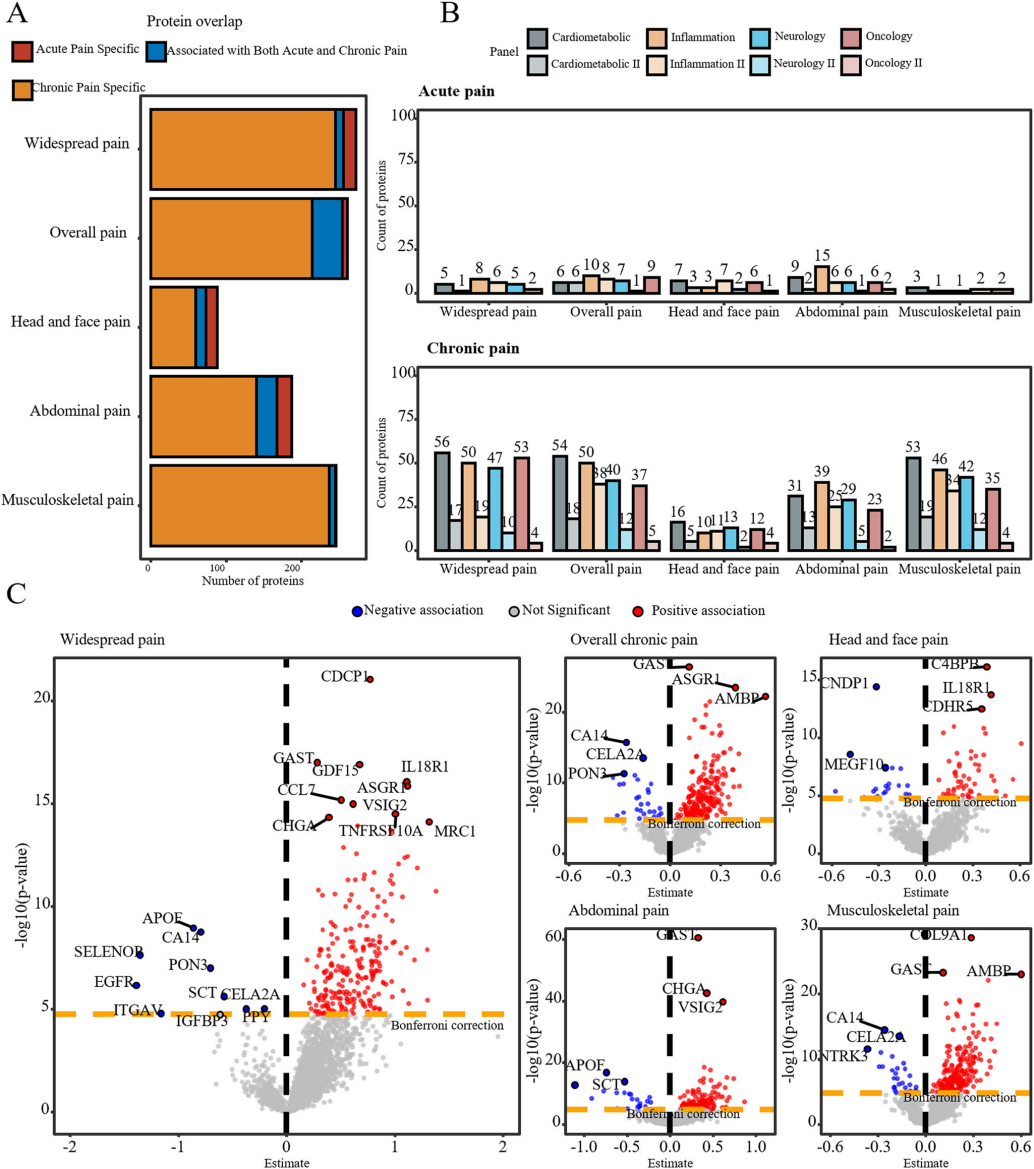
Proteomic signature of major types of chronic pain and acute pain. Notes: A, Comparison of proteins significantly associated with both chronic and acute pain. B, Count of proteins significantly associated with specific types of acute and chronic pain, categorized by panel. C, Volcano plots show protein associations with different pain types after applying Bonferroni correction. Proteins with p‐values below 0.05/2920 are highlighted in red (positive) and blue (negative), while nonsignificant associations are represented in gray. The top 10 positively and negatively associated proteins with the lowest p‐values for each pain type are labelled.

We conducted a proteome‐wide association study (PWAS) to evaluate the ability of protein signatures to distinguish chronic pain by comparing protein expression profiles between individuals with chronic pain and pain‐free controls. People with chronic pain showed widespread and substantial changes in protein expression compared to pain‐free controls. In contrast, those with acute pain showed minimal associations with protein levels (**Figure** **1**A and Figures S6, Supporting Information). To assess the difference in protein signatures associated with chronic and acute pain, we analyzed the overlap between proteins linked to each condition. Only 16% (40/254) of the proteins associated with chronic pain overlapped with those linked to acute pain, underscoring distinct molecular pathways (Figure 1A,B and Table S3, Supporting Information).

In the analysis of widespread chronic pain, the proteins most significantly upregulated compared to controls were CDCP1, IL18R1, GAST, GDF15, ASGR1, CCL7, VSIG2, CHGA, TNFRSF10A, and MRC1. In contrast, the ten most downregulated proteins were CA14, APOF, PON3, SELENOP, EGFR, ITGAV, SCT, CELA2A, IGFBP3, and PPY (Figure 1B). A total of 256 proteins out of 2920 (8.7%) exhibited significant associations with widespread chronic pain. In the PWAS, 254 out of 2920 proteins (8.7%) exhibited significant associations with overall chronic pain. Musculoskeletal pain had the highest number of significant associations, with 245 proteins (8.7%), followed by abdominal pain with 167 proteins (5.7%), and head and face pain with 73 proteins (2.5%) (Figure 1B and Table S4, Supporting Information). Several proteins, including APOF, ASGR1, CA14, CA6, MXRA8, PLTP, PON3, and RARRES2, were shared across multiple pain types (Figure 1C).

### 2.3 Functional Enrichment of Proteins Associated with Widespread Chronic Pain

We performed GO enrichment analysis on proteins significantly associated with widespread chronic pain. The most enriched biological processes involved positive regulation of leukocyte migration, regulation of cell activation, and symbiont entry into host, implicating immune activation and host–pathogen interactions as central mechanisms. Enriched cellular components, including lysosomal lumen, vacuolar lumen, and external side of the plasma membrane, indicated roles in intracellular trafficking and membrane‐associated immune processes. For molecular functions, terms such as signaling receptor activity, virus receptor activity, and death receptor activity were highly represented, highlighting dysregulated cytokine and death receptor signaling.

KEGG enrichment further confirmed immune and inflammatory involvement, identifying cytokine–cytokine receptor interaction, viral protein interaction with cytokine and cytokine receptor, and lysosome as top pathways. Reactome analysis similarly emphasized immune dysregulation, with significant enrichment in Interleukin‑10 signaling, TNF binding their physiological receptors, TNFR2 non‑canonical NF‑κB signaling, and cytokine signaling in the immune system.

Together, these results indicate that widespread chronic pain is driven by coordinated dysregulation of immune signaling, cytokine‐receptor interactions, and lysosome‑mediated processes (Figure S7, Supporting Information).

### 2.4 Protein Signatures Improved Classification of Widespread Chronic Pain

Proteins identified from PWAS for widespread chronic pain and specific chronic pain types were used to derive comprehensive proteomic scores (C‐ProtS) for chronic pain. Additionally, simple proteomic scores (S‐ProtS) were generated using the top 10 proteins associated with widespread chronic pain and specific chronic pain types to evaluate the potential of these simple plasma protein signatures in distinguishing specific chronic pain types.

**Figure 2:**
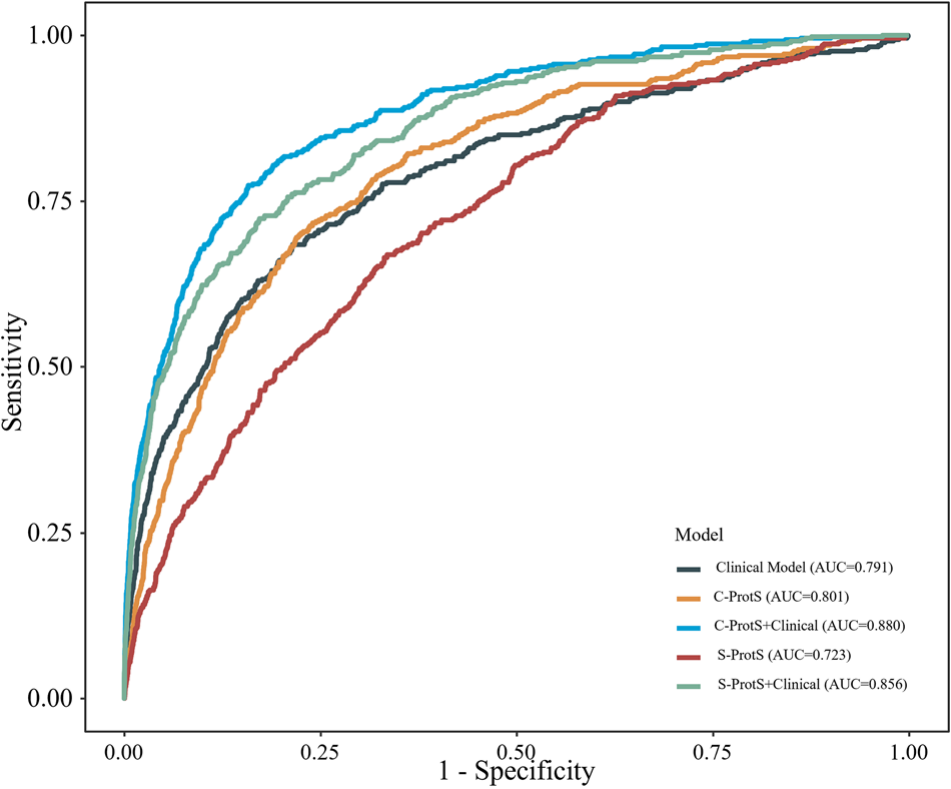
Diagnostic performance of the proteomic score, clinical score, and combined. Notes: This figure presents the ROC curves and Area Under the Curve (AUC) for widespread chronic pain. Higher AUC values indicate improved model performance in distinguishing individuals with widespread chronic pain from those without. Abbreviations: CS: clinical risk model; S‐ProtS: simple proteomic score; C‐ProtS: comprehensive proteomic score; CS+S‐ProtS: combined model with clinical model and S‐ProtS; CS+C‐ProtS: combined model with clinical model and C‐ProtS.

Clinical risk models (CS) incorporating previously^[^ ^13^ ^]^ identified key risk factors like sleep difficulties, neuroticism, powerlessness, mental health issues, life stressors, and obesity achieved a median Area Under the Curve (AUC) of 0.791 (95% CI: 0.767, 0.814) for widespread chronic pain. A protein‐only model based on S‐ProtS showed comparable performance, with an AUC of 0.723 (95% CI: 0.700,0.746). The C‐ProtS model achieved numerically higher performance, with an AUC of 0.801 (95% CI: 0.780,0.822). Incorporating C‐ProtS into the CS model enhanced discrimination, yielding an AUC of 0.856 (95% CI: 0.838,0.874) for widespread chronic pain. The combined C‐ProtS and CS model reached the highest discrimination, with an AUC of 0.880 (95% CI: 0.864,0.897) (**Figure** **2**A and Table S5, Supporting Information). To assess the interpretability of the classification models, we evaluated their calibration and performed decision curve analyses. For widespread chronic pain, the combined models demonstrated good calibration, as evidenced by a calibration slope of 0.860 (95% CI 0.803–0.916), indicating strong alignment between predicted and observed risks (Figure S8, Supporting Information).

### 2.5 Prospective Prediction of Pain Progression and Onset

**Figure 3:**
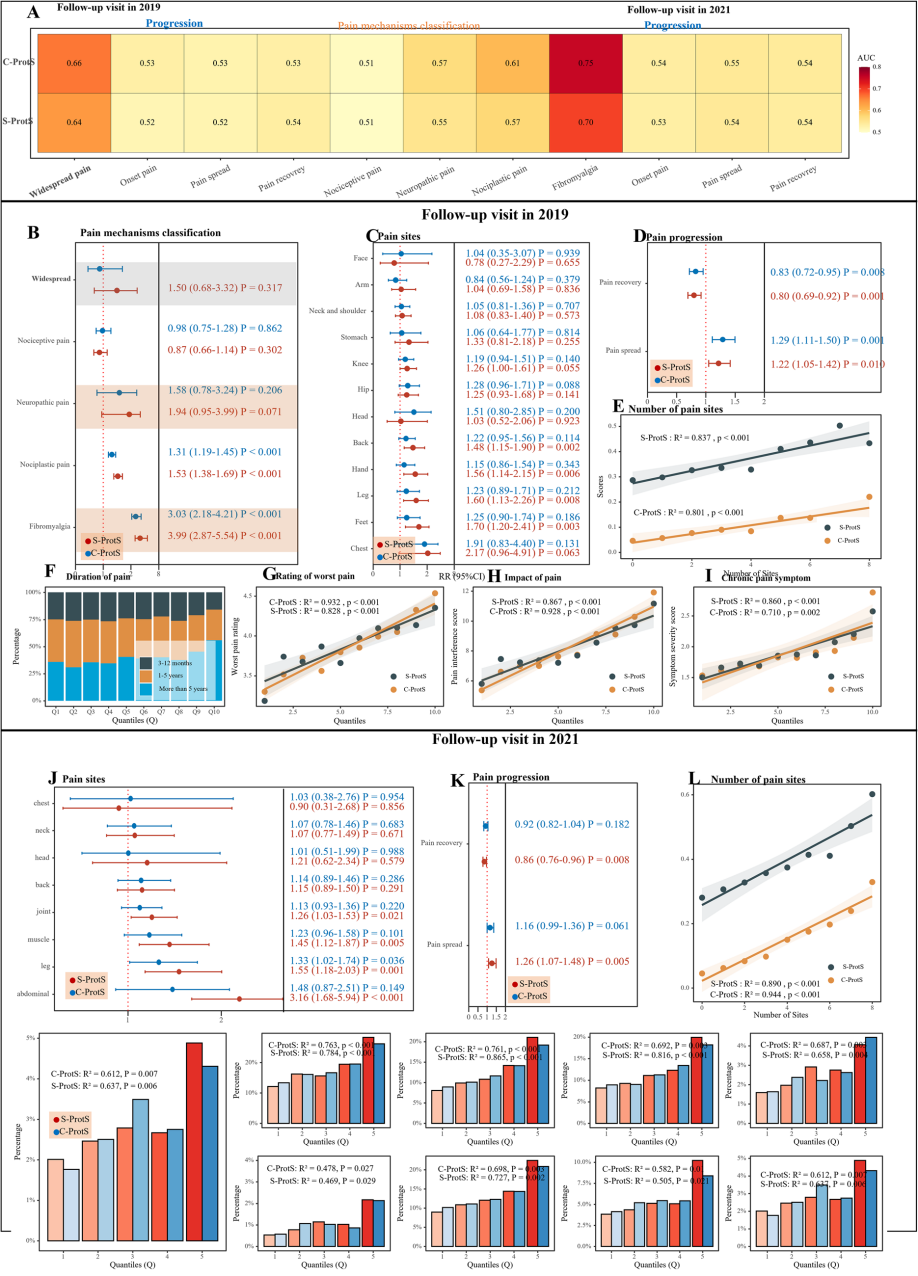
Prospective associations of pain‐specific proteomic scores with mechanism pain classification, pain onset, progression, multisite chronic pain, duration, intensity, impact, and activity limitations in longitudinal data. Notes: The 2019 follow‐up refers to the online follow‐up conducted in 2019, and the 2022 follow‐up refers to the one conducted in 2022. Panel A shows the AUC of C‐ProtS and S‐ProtS for selected pain types. Panel B: Displays the association between the onset of widespread chronic pain, pain mechanism classification, and the proteomic scores (ProtS) in 2019, adjusted for age, gender, sleeplessness, feeling fed‐up, tiredness, mood, stressful life events, and BMI. Panels C and J: Show the associations between the onset of chronic pain and the proteomic scores in 2019 and 2022, respectively, adjusted for the same covariates. "Onset of chronic pain" includes participants who were pain‐free at baseline but reported chronic pain at follow‐up. Panels D and K: "Pain spreading" refers to participants whose number of pain sites increased, while "pain recovery" refers to those whose pain sites decreased. Examining pain spreading and pain recovery, also adjusted for covariates. Panels E and L: Depict the number of pain sites (multisite chronic pain) plotted against quantiles of the Simple Proteomic Score (S‐ProtS) and Comprehensive Proteomic Score (C‐ProtS). Panel F: Shows the proportion of chronic pain duration across quantiles of C‐ProtS. Panels J‐I: Illustrate the rating of worst pain, impact of pain, and symptom severity, respectively, in relation to quantiles of S‐ProtS and C‐ProtS. Panel M: Illustrates the proportion of activity limitations caused by overall chronic pain among participants with chronic pain. Panel N: Shows activity limitations by specific chronic pain sites. Abbreviations: S‐ProtS, Simple Proteomic Score; C‐ProtS, Comprehensive Proteomic Score.

The stability and individual changes in pain site numbers between baseline and the two follow‐up visits are shown in Figure S9 (Supporting Information). We assessed the predictive power of C‐ProtS and S‐ProtS for predicting new widespread pain, pain mechanisms, and pain progression. Both C‐ProtS and S‐ProtS demonstrated good predictive discrimination for fibromyalgia, with AUCs of 0.75 and 0.70, respectively (**Figure** **3**A).

Both C‐ProtS and S‐ProtS for widespread chronic pain were significantly associated with the onset of widespread chronic pain at follow‐up visits (Figure 3B). In the pain mechanism classification, C‐ProtS and S‐ProtS were significantly associated with nociplastic pain and fibromyalgia. However, for nociceptive pain and neuropathic pain, no significant associations were observed for either C‐ProtS or S‐ProtS (Figure 3B).

For the onset of specific pain sites, C‐ProtS and S‐ProtS also showed significant positive associations, although these were weaker for certain pain sites, such as headache, facial pain, and abdominal pain, compared to other chronic pain sites (Figure 3C,J). Both follow‐up visits demonstrated a positive correlation between proteomic scores and pain spread, and a negative correlation with pain recovery. Higher proteomic scores were associated with an increased number of pain sites (multisite chronic pain), while lower scores corresponded with fewer pain sites and greater pain recovery (Figure 3D,E,K,L). The proteomic scores, both C‐ProtS and S‐ProtS, demonstrated a strong association with the number of pain sites at the 2019 follow‐up (C‐ProtS: R^2^ = 0.837, P<0.001; S‐ProtS: R^2^ = 0.801, P<0.001) and an even stronger association at the 2022 follow‐up (C‐ProtS: R^2^ = 0.890, P<0.001; S‐ProtS: R^2^ = 0.944, P<0.001) (Figure 3C,G).

Proteomic scores exhibited a monotonic increase with pain duration, rating of worst pain, pain impact, and pain symptom severity nine years later (Figure 3F–I). The percentage of participants reporting activity limitations due to chronic pain increased across higher proteomic score quantiles (C‐ProtS and S‐ProtS) (Figure 3L). S‐ProtS showed comparable associations with activity limitations related to chronic pain sites, similar to those observed with C‐ProtS (Figure 3N).

### 2.6 Contributions of Proteins to the Classification of Widespread Chronic Pain

**Figure 4:**
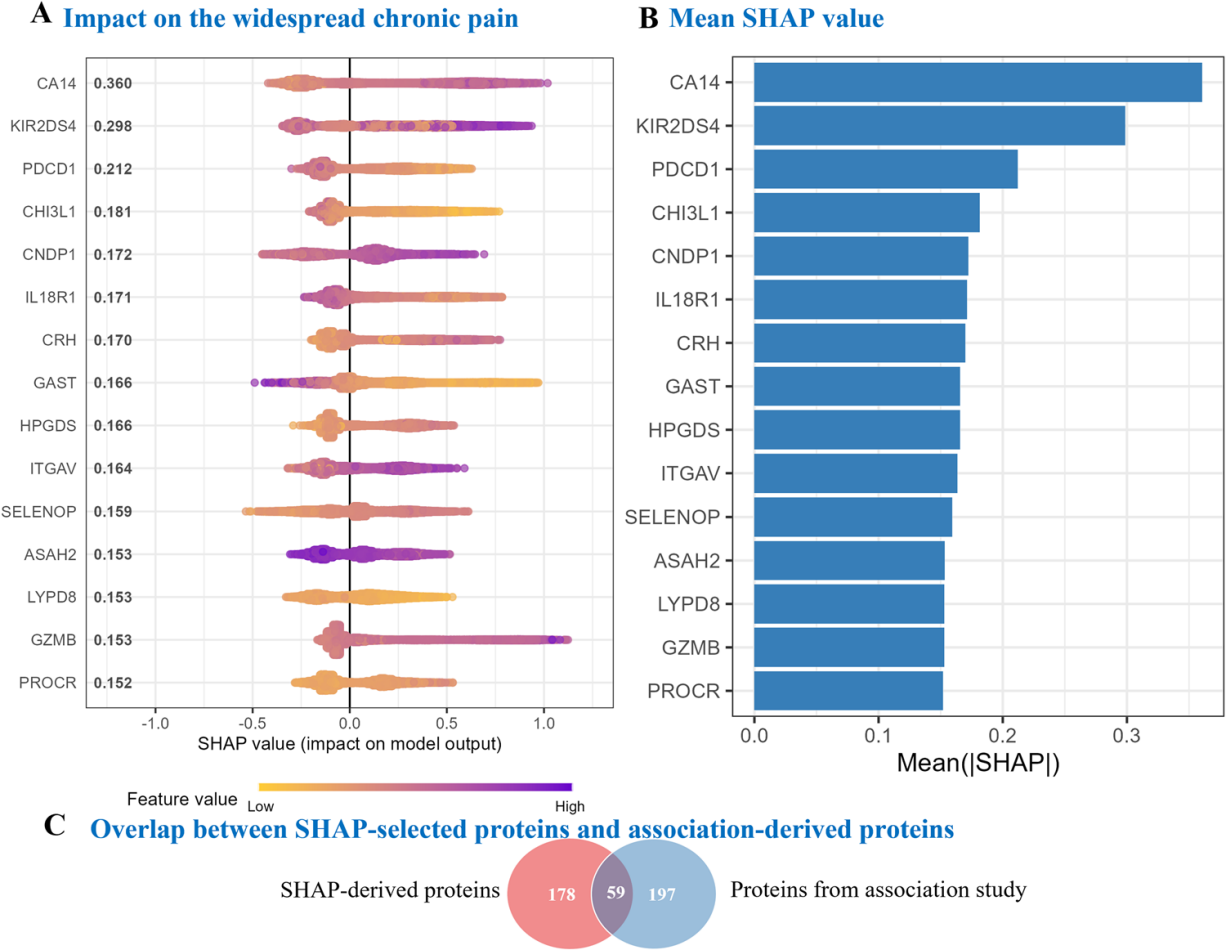
SHAP of protein contributions to widespread chronic pain. Notes: (A) SHAP plot illustrating the impact of individual proteins on widespread chronic pain prediction. Brighter colours (yellow) represent higher feature values, while darker colours (purple) indicate lower feature values, demonstrating how each protein contributes to the prediction model. (B) Mean SHAP values for the top contributing proteins to widespread chronic pain. Abbreviations: SHAP, SHapley Additive exPlanations. (C) Overlap between SHAP‐selected proteins and association‐derived proteins.

Proteins, including CA14, KIR2DS4, PDCD1, CHI3L1, CNDP1, IL18R1, CRH, GAST, HPGDS, ITGAV, SELENOP, ASAH2, LYPD8, GZMB, and PROCR, were identified as key contributors to widespread chronic pain. Among these, CA14 emerged as the most impactful, reflected by its highest SHAP (SHapley Additive exPlanations) value, suggesting a prominent role in pain prediction. KIR2DS4 and PDCD1 followed as major contributors. The SHAP analysis further highlighted the role of GAST, where both high and low levels influenced risk prediction. Elevated GZMB values (indicated by purple) are particularly associated with an increased risk of widespread chronic pain (**Figure** **4**).

To comprehensively prioritize protein biomarkers for widespread chronic pain, we integrated machine learning‐derived feature importance (non‐zero SHAP values) with traditional association analyses. A total of 237 proteins exhibited non‐zero SHAP values, while epidemiological analysis identified 256 proteins significantly associated with widespread chronic pain. Notably, 59 proteins were shared between both approaches, representing high‐confidence candidates for mechanistic and therapeutic investigation. The union of these datasets yielded 434 unique proteins, constituting a robust resource for subsequent biological and translational studies.

### 2.7 Mendelian Randomization and Causal Inferences

To investigate potential causal relationships between circulating proteins and pain phenotypes, we performed Mendelian randomization (MR) analyses on a combined set of 434 prioritized proteins. Pain outcomes included chronic widespread pain from the UKB, a European meta‐analysis cohort excluding UKB, and multisite chronic pain from UKB, enabling a comprehensive evaluation of causality across clinically relevant endpoints.

**Figure 5:**
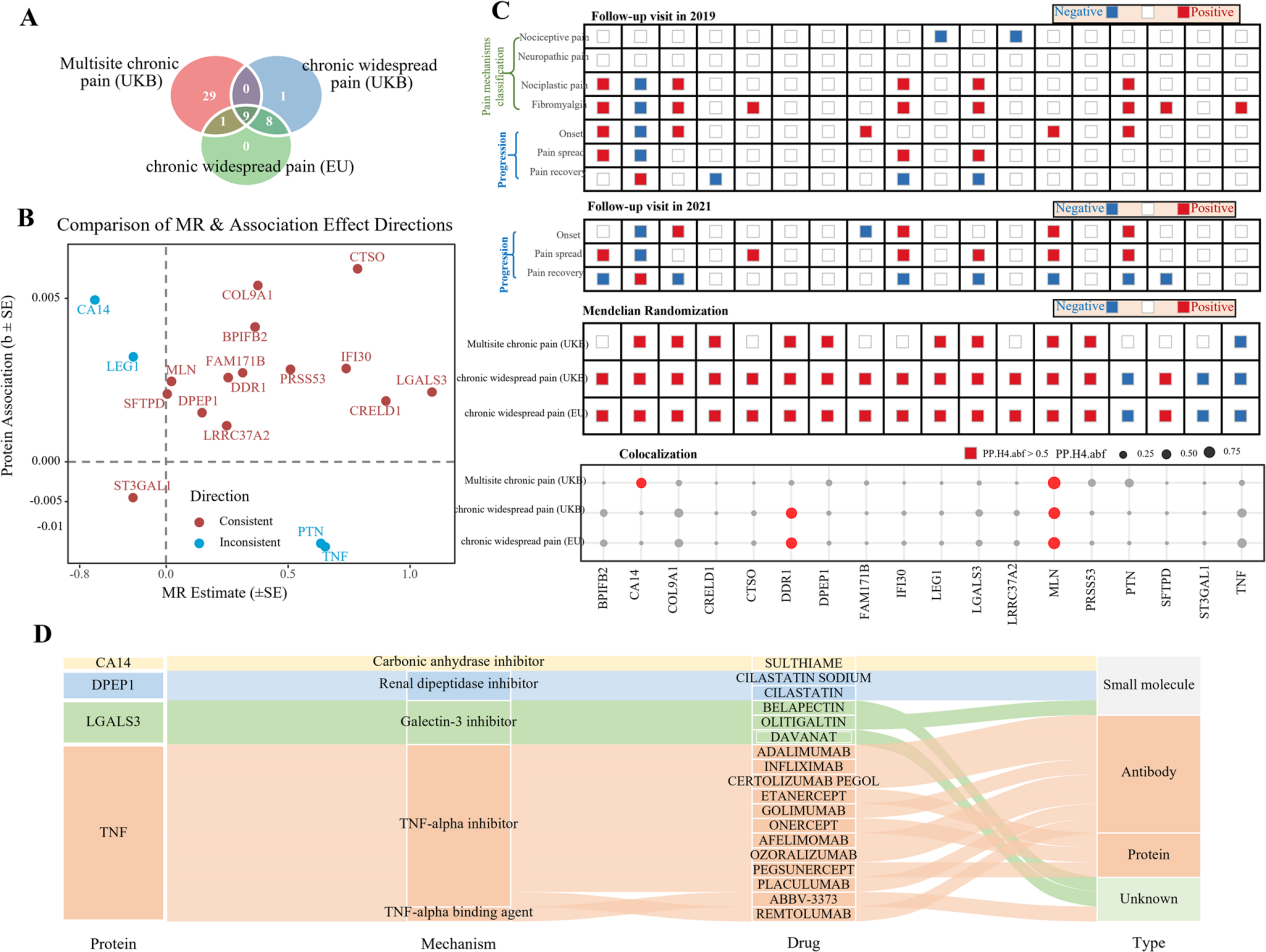
Integrated causal, longitudinal, and pharmacological characterization of top protein biomarkers for widespread chronic pain. Notes:   (A) Overlap of proteins with significant causal associations across three phenotypes: CWP in UKB, CWP in European meta‐analysis, and multisite chronic pain in UKB. (B) Comparison of MR estimates and epidemiological associations for the 18 MR‐identified proteins (concordant: red; discordant: blue). (C) Multi‐layer evidence: top—prospective associations with pain type, onset, spread, and recovery (UKB 2019/2021); middle—MR across three phenotypes; bottom—colocalization (CA14, DDR1, MLN with PP.H4 > 0.5). (D) Drug repurposing landscape for targetable proteins (CA14, DPEP1, LGALS3, TNF) by mechanism and therapeutic type.

We designated the European meta‐analysis of chronic widespread pain as the primary MR outcome, using UKB multisite chronic pain and UKB chronic widespread pain as independent replication cohorts. In the primary MR analysis, 18 proteins demonstrated significant causal associations with widespread pain. Of these, 10 were validated in the UKB multisite chronic pain cohort, and 17 in the UKB chronic widespread pain cohort, with 9 proteins consistently validated in both replication analyses (**Figure** **5**A). Notably, 14 of the 18 proteins showed concordant effect directions between epidemiological association and MR results, while four proteins—PTN, TNF, CA14, and LEG1—exhibited discordant directions. The 18 proteins identified include CA14, COL9A1, CRELD1, DPEP1, LEG1, LGALS3, MLN, PRSS53, TNF, BPIFB2, CTSO, DDR1, FAM171B, IFI30, LRRC37A2, PTN, SFTPD, and ST3GAL1 (Figure 5B).

To further elucidate shared genetic architecture, we conducted colocalization analysis. Three proteins—CA14, DDR1, and MLN—demonstrated strong evidence of colocalization with pain phenotypes (PP.H4 > 0.5), suggesting that their associations are likely attributable to shared causal variants (Figure 5C).

### 2.8 Prospective Association of Top 18 Proteins with Pain Phenotypes

We investigated the prospective associations between levels of the top 18 proteins identified for widespread chronic pain and pain mechanisms (nociceptive pain, neuropathic pain, nociplastic pain, and fibromyalgia), as well as the onset, spread, and recovery of chronic pain.

9 proteins (BPIFB2, CA14, COL9A1, CTSO, IFI30, LGALS3, PTN, SFTPD, and TNF) exhibited significant associations with nociplastic pain or fibromyalgia. 2 of the 15 proteins (LEG1 and LRRC37A2) showed significant associations with nociceptive pain or neuropathic pain, indicating that these 14 proteins can effectively distinguish nociplastic pain/fibromyalgia from nociceptive and neuropathic pain up to 9 years in advance (Figure 5C). At the 2021 follow‐up, the onset and progression of chronic pain showed significant associations with the 10 proteins (BPIFB2, CA14, COL9A1, CTSO, FAM171B, IFI30, LGALS3, MLN, PTN, and SFTPD). Among these, BPIFB2, CA14, COL9A1, IFI30, LGALS3, MLN, and PTN exhibited significant associations with all aspects of chronic pain onset and progression, including pain spreading and recovery (Figure 5C). The detailed prospective associations between the top 18 proteins and pain mechanism classification (nociceptive pain, neuropathic pain, nociplastic pain, and fibromyalgia), as well as the onset and progression of chronic pain, including non‐linear associations, are presented in the Figure S10–S28 (Supporting Information).

### 2.9 Proteomics‐Based Drug Repurposing

To evaluate the translational potential of our protein prioritization strategy, we systematically interrogated Open target databases for therapeutic agents targeting the 18 proteins identified by MR as causally linked to widespread chronic pain. Of these, four proteins—CA14, DPEP1, LGALS3, and TNF—had existing pharmacological modulators, either approved or in clinical development, encompassing 18 distinct compounds across small molecules, antibodies, and protein‐based biologics (Table S5, Supporting Information).

CA14, a carbonic anhydrase expressed in neuronal tissues, is targeted by sulthiame, an antiepileptic drug approved for obstructive sleep apnea and seizure disorders. Given the observed non‐linear associations between CA14 levels and pain phenotypes, CA14 agonists—rather than inhibitors—may offer greater therapeutic utility in this context (Figure S29, Supporting Information).

DPEP1, a renal dipeptidase involved in inflammatory responses, is inhibited by cilastatin, a small molecule co‐administered with β‐lactam antibiotics to reduce renal toxicity in infectious disease settings.

LGALS3 (galectin‐3), a regulator of immune signaling and fibrosis, is targeted by several investigational agents, including belapectin, davanat, and olitigaltin, with indications ranging from non‐alcoholic steatohepatitis to fibrotic lung disease and cancer.

TNF, a master cytokine in inflammatory signaling, is the target of multiple approved biologics—such as adalimumab, infliximab, and etanercept—used in immune‐mediated diseases including rheumatoid arthritis, Crohn's disease, and psoriasis.

Collectively, these findings point to multiple actionable opportunities for drug repurposing in nociplastic pain, supported by proteome‐wide causal inference. In particular, TNF antagonists and galectin‐3 inhibitors emerge as strong candidates for translational advancement in pain therapeutics (Figure 5D).

## 3 Discussion

Our analysis revealed marked differences in the protein signatures of chronic versus acute pain. By leveraging these protein changes, we developed pain‐specific proteomic scores that not only effectively discriminated individuals with chronic pain from those who were pain‐free, but also predicted the intensity, phenotype, onset, spread, and recovery of chronic pain, particularly widespread chronic pain, a key feature of nociplastic pain. Notably, a comprehensive proteomic signature based on the 10 proteins performed comparably to models based on conventional clinical variables alone. Both the simple and comprehensive proteomic scores significantly enhanced the predictive performance when added to clinical predictors. Among the 434 proteins identified through machine learning–based feature importance and conventional association analyses, 18 demonstrated evidence of causal relevance supported by Mendelian randomization analysis, with 9 proteins significantly associated with specific pain mechanisms. Notably, four of these 18 proteins have existing drugs targeting them. CA14, identified as the top‐ranking protein and supported by both MR and colocalization analyses, is a known target of sulthiame (CHEMBL328560), suggesting a potential therapeutic target for nociplastic pain conditions such as fibromyalgia.

### 3.1 Distinct Protein Signatures in Chronic and Acute Pain

Chronic pain exhibits more extensive protein changes compared to acute pain. Traditionally, chronic pain is defined primarily by its duration and lacks specific biomarkers to guide clinical management. Our study addresses his challenge by identifying distinct proteomic signatures that differentiate between chronic and acute pain, suggesting that proteomic information could predict those who will transition from acute to acute pain. Previous research has highlighted the utility of proteomics in understanding pain sensitivity, especially in experimental models.^[^ ^18^ ^]^ Notably, several proteins identified in our study provide novel insights into the biological pathways involved in pain mechanisms, in particular highlighting the involvement of metabolic and immune pathways in chronic pain development and persistence. For instance, leptin (LEP), known for its role in energy regulation, was strongly associated with chronic pain, indicating the potential involvement of metabolic dysregulation. Additionally, proteins such as ASGR1 and RARRES2, involved in immune responses, highlight the role of inflammation in chronic pain mechanisms. Inflammatory biomarkers may aid in predicting high‐risk groups for developing nociplastic pain and guide the development of tailored treatments.

The distinct molecular profiles observed across specific pain types, such as head and face pain, abdominal pain, and musculoskeletal pain, further emphasize the heterogeneity of chronic pain. However, the identification of shared proteins across multiple pain types — such as APOF and ASGR1— suggests that there may be common underlying biological mechanisms driving pain across body regions. This finding could pave the way for the development of universal biomarkers that apply across multiple pain syndromes.

### 3.2 Classification Accuracy of Proteomic Profiling

We demonstrate that proteomic profiling can perform as well as conventional clinical risk scores in predicting chronic pain and its progression. Moreover, combining proteomic scores with clinical variables further enhances predictive performance, suggesting that an integrated approach may be beneficial. This study is the first to comprehensively demonstrate the predictive power of plasma proteins across a broad spectrum of chronic pain types. We achieved this by directly comparing proteomic data with clinical variables and evaluating the added predictive value that proteomics offers beyond traditional clinical predictors. The proteomic score alone exhibited high predictive accuracy for chronic pain and its various subtypes. This predictive performance was further enhanced when proteomic data were combined with clinical variables. Our findings underscore the substantial potential of proteomic profiling as a promising technique to complement clinical evaluation, aiding in both the risk assessment for transition to chronic pain and the prognostication of chronic pain outcomes.

The proteomic score demonstrated a strong association with the onset, spread, and recovery of chronic pain. This profile effectively identifies individuals at higher risk for developing chronic pain, experiencing its spread to new sites, and their likelihood of recovery. Pain is influenced by a complex interplay of biological, psychological, and social factors.^[^ ^13^ ^]^ Proteins integrate genetic, environmental, age‐related, behavioral, and medication influences, offering comprehensive insights into pain mechanisms. Previous research has suggested that pain spread is not random but shows strong dependencies between adjacent pain sites.^[^ ^13^ ^]^ Our study supports this by revealing significant associations between pain‐specific proteomic scores and pain at various sites. Additionally, we observed robust correlations between proteomic scores and pain recovery, indicating shared biological foundations for both the spread and recovery of pain.

The proteomic score demonstrated a significant and specific association with nociplastic pain and fibromyalgia, distinguishing them from nociceptive and neuropathic pain. Nociplastic pain is characterized by abnormal sensory processing without clear peripheral damage. The association between proteomic score and nociplastic pain suggests that proteomic signatures capture the complex biological underpinnings of this condition, including abnormalities in sensory perception, a hallmark feature.^[^ ^11^ ^]^

### 3.3 Causal Relevance and Therapeutic Potential of Key Proteins

Carbonic anhydrase 14 and leptin were identified as the primary proteins consistently associated with chronic pain, particularly nociplastic pain and fibromyalgia, and progression of chronic pain (onset, spreading, and recovery of chronic pain) in both longitudinal analyses. carbonic anhydrase 14, identified here for the first time as associated with chronic pain, plays an important role in neuronal signal transmission via zinc ion binding and carbonate dehydratase activity. It is primarily enriched in the spinal cord and is crucial for neuronal function. Leptin is involved in immune regulation and inflammation, contributing to pain modulation, especially in nociplastic pain. Fibromyalgia affects 10–48% of patients with rheumatic diseases, compared to ≈2–6% in the general population, likely due to shared mechanisms of immune dysregulation and inflammation.^[^ ^19^, ^20^, ^21^ ^]^ Both leptin and gastrin showed positive correlations with new‐onset pain and pain spread, suggesting their role in the development and propagation of chronic pain. This relationship is further supported by their impact on pain recovery outcomes. Animal studies have demonstrated that leptin can influence pain thresholds.^[^ ^22^, ^23^ ^]^ Together, these proteins suggest potential complementary roles in pain, with leptin influencing inflammation and carbonic anhydrase 14 affecting neuronal signaling.

The role of carbonic anhydrase 14 and leptin were both supported by mendelian randomization, with carbonic anhydrase 14 further validated through colocalization analysis. Our search on the Open Targets platform revealed that carbonic anhydrase 14 is the target of an approved small molecule drug, sulthiame, which is currently used to treat epilepsy and obstructive sleep apnoea. However, there are currently no well‐documented drugs that activate CA14. Most research on carbonic anhydrase modulation has centered on inhibitors rather than activators, as seen with sulthiame in epilepsy treatment. If enhancing CA14 activity proves to be therapeutically relevant for pain management, future research will need to focus on developing specific agonist or allosteric modulators. In addition to carbonic anhydrase 14, drugs for four other proteins were identified, primarily used for cancer and autoimmune diseases. While these therapies carry potential risks related to immune modulation, they remain possibilities for treatment options. Carbonic anhydrase 14, the top‐ranked protein with a strong causal association, suggests that targeting this protein could offer a promising therapeutic avenue for nociplastic pain.

A notable observation in our study is the inverse directionality between observational and MR associations for certain proteins, such as CA14, LEG1, PTN, and TNF. This inverse directionality is not unique to our study and likely reflects the distinction between state‐dependent protein alterations versus lifelong genetic predisposition. For instance, while CA14 and LEG1 were negatively associated with pain cross‐sectionally, MR suggested protective effects of genetically elevated expression. In contrast, PTN and TNF were upregulated in pain states but showed inverse effects in MR, highlighting the complex, context‐specific roles of inflammatory and neuroimmune pathways in pain biology.

### 3.4 Limitations and Strengths

While our study provides strong evidence for the role of proteomic profiling in characterisitng chronic pain, there are limitations which warrant discussion. First, while proteomic profiling demonstrated predictive value, the Olink panels used may have excluded key proteins relevant to chronic pain, potentially limiting the comprehensiveness of our analysis. Future studies should incorporate a wider array of proteomic markers to capture a more complete picture of the biological pathways involved in chronic pain. Second, the focus was primarily on a specific subset of pain types. Other pain characteristics not included may have different proteomic profiles and predictors. Furthermore, some participants in the control group, despite being classified as pain‐free, may have had a history of chronic pain. Third, the study population was predominantly white British, which may limit the generalizability of the findings to other ethnic groups. Proteomic profiles and pain associations could vary across different genetic backgrounds. Fourth, while l CA14, DDR1, and MLN showed a causal relationship with pain, potential protein‐protein interactions were not considered. The underlying biological pathways also remain unclear, warranting further research. Fifth, the findings have not been validated in independent cohorts. External validation in other large and diverse populations is necessary to confirm the robustness and applicability of the results. A potential limitation is the partial sample overlap between the protein and pain GWAS datasets, as both were derived from the UK Biobank. While all instruments had F‐statistics > 10 and sensitivity analyses supported the robustness of the results, some bias toward the null cannot be excluded.^[^ ^24^ ^]^ We acknowledge that the MR findings would be more robust if there were evidence indicating that the pQTLs and genetic variations associated with the outcome are shared. However, we also recognize that the lack of colocalization evidence does not undermine the validity of the results, as colocalization methods are known to have a high false‐negative rate, typically ≈60%.^[^ ^24^ ^]^ Finally, the top proteins were prioritized based on SHAP values derived from the cross‑validated model trained on the full dataset. However, as no independent test set was available, we cannot directly assess the generalizability of these features. Future studies with external cohorts are needed to validate the predictive performance and robustness of these selected proteins.

## 4 Conclusion

In conclusion, this study underscores the significant potential of proteomic profiling to advance our understanding and management of chronic pain, particularly nociplastic pain, and fibromyalgia. By coupling machine learning–based prioritization with genetic validation, we identified 18 circulating proteins with putative causal roles in widespread chronic pain, several of which—such as CA14, DDR1, and MLN—also demonstrated colocalized genetic regulation. Importantly, four of these proteins (CA14, DPEP1, LGALS3, and TNF) are targetable by approved or investigational therapeutics, revealing immediate opportunities for drug repurposing. TNF inhibitors and galectin‐3 antagonists, in particular, emerge as promising candidates for translation into clinical pain management.

## 5 Experimental Section

### Study Population

The UK Biobank was a prospective, population‐based cohort comprising over 500 000 individuals aged 40–69, recruited from 22 assessment centers across the UK between 2006 and 2010. In 2019, additional online surveys were conducted to track specific pain‐related outcomes over time. Plasma proteomic data were available for 53029 UK Biobank participants, comprising a randomly selected subset at baseline and 6229 individuals preselected by the UK Biobank Pharma Proteomics Project (UKB‐PPP). To minimize potential selection bias, individuals from the UKB‐PPP subgroup were excluded. After quality control (Figure S1, Supporting Information), proteins were excluded with >50% missing values (n = 3), and samples with >50% missing proteins (n = 6709). Participants reporting acute pain were also excluded (n = 7755), as were those with any cancer diagnosis or from the UKB‐PPP group (n = 9311), yielding a final analytic sample of 29254 participants with high‐quality proteomic and phenotype data. More details of the study design, cohort construction, and study window are provided in Figures S1 and S2 (Supporting Information). Ethical approval was obtained from the North West Multi‐centre Research Ethics Committee (MREC, https://www.ukbiobank.ac.uk/learn‐more‐about‐uk‐biobank/about‐us/ethics), with all participants providing written informed consent. This research was approved under application number 98 358.

### Inclusion and Exclusion Criteria

To ensure biological specificity and minimize confounding, analysis‑specific exclusion criteria were applied, reflecting the well‑established mechanistic differences between acute and chronic pain. Chronic pain involves long‐term central sensitization, neuroimmune dysregulation, and psychosocial modulation, whereas acute pain was typically short‐term and self‐limited. Participants with acute pain (≤3 months) were excluded from chronic pain analyses, whereas those with chronic pain (>3 months) were excluded from acute pain analyses. Individuals were also excluded with any history of cancer, as cancer‐related pain arises from tumor invasion or oncologic treatment and may reflect distinct biological processes. Finally, participants who were not randomly selected—specifically those recruited through the UKB‐PPP—were excluded due to their non‐random sampling and potential selection bias. These exclusions were made to improve etiological coherence and ensure that the observed proteomic associations reflect mechanisms underlying chronic, non‐cancer pain in the general population.

### Clinical Variables

Six variables capturing biological, psychological, and social factors were included as predictors for the construction of a clinical model. They were specified in prior with literature evidence suggesting their predictive value for chronic pain.^[^ ^13^ ^]^ The factors were collected by an standardized touchscreen questionnaire in UK Biobank and include sleep difficulties (difficulty falling asleep, rated as quite a lot or very much), neuroticism (perceived life effort as constant, rated as quite a lot or very much), powerlessness (feelings of lack of energy or powerlessness, rated as quite a lot or very much), mental health issues (history of diagnosis or treatment by a doctor), life stressors (events such as divorce, death of a partner, or significant unemployment history), and a BMI over 30 kg m^−2^. All the variables listed above were transformed into binary variables (Table S1, Supporting Information). Binary thresholds were defined as follows: sleeplessness and feeling “fed‐up” were scored as positive if experienced “usually”; tiredness was scored positive if reported “more than half the days”; mood disturbance was coded as “yes” if participants reported feeling nervous, anxious, tense, or depressed; stressful life events included events such as bereavement or unemployment; and BMI > 30 kg m^−^^2^ was considered obese.

### Proteomic Profiling

The UK Biobank Pharma Proteomics Project consortium has generated extensive blood‐based proteomic data. Proteomic profiling was conducted on EDTA‐plasma samples from a cohort of 54893 UKB participants at baseline recruitment, derived from a randomized subset representative of the broader UKB population. Utilising the Olink Explore 1536 and Expansion platforms, the profiling encompassed 2920 unique proteins, assessed through 2941 assays. The protein targeting assays were organized into four 384‐plex panels: Inflammation, Oncology, Cardiometabolic, and Neurology. Detailed methodologies concerning assay execution, sample selection, and handling were available in the online document (https://biobank.ndph.ox.ac.uk/showcase/label.cgi?id = 1839). No notable impacts from batch and plate variations, nor anomalies in protein coefficients of variation (CVs), were detected. The inter‐ and intraplate CVs for all OLINK panels remained below 20% and 10%, respectively, with a median protein CV of 6.7%. There were strong correlations observed for identical proteins across different panels and between the OLINK assay and independent assays within the UK Biobank. The proteomics data were provided as Normalized Protein Expression (NPX) values on a log_2_ transformation.

This analysis focuses on participants with less than 50% missing proteomic data. Additionally, three proteins missing in over 50% of participants were excluded. Post‐quality control, missing NPX values were imputed using chained random forests, incorporating age and sex as predictors in the imputation process. The NPX data of proteins were standardized to have a mean of 0 and a variance of 1, following a standard normal distribution.

### Baseline Pain Phenotype

At the baseline recruitment between 2006 and 2010, 501243 participants were asked about pain experiences that interfered with their usual activities in eight major body regions in the past one month.

Participants who reported pain at a specific site in the past month were subsequently asked whether the pain had persisted for more than three months. This follow‐up question was used to define chronic pain (pain persisting for more than three months) and acute pain (having pain in the last one month but persisting for three months or less). Among the eight painful locations specifically asked in the questionnaire, They were grouped into four categories based on the similarity of underlying aetiologies and conditions: (1) headache and facial pain, which may reflect conditions like migraines or tension headaches; (2) abdominal pain, potentially linked to visceral causes such as irritable bowel syndrome (IBS) or endometriosis; (3) musculoskeletal pain (neck or shoulder pain, hip pain, and knee pain), which may indicate localized mechanical or inflammatory issues; and (4) widespread pain (“pain all over the body”), often associated with conditions like fibromyalgia. For widespread pain, participants who reported experiencing it were not given the option to select other specific body regions.

### Longitudinal Pain Phenotypes—Online UK Biobank in 2019 – Pain Mechanism Classification

Pain outcomes were classified based on participants' chronic pain status and diagnostic criteria derived from validated scales. In 2019, participants were invited to complete an online pain questionnaire that incorporated the 2016 revised Fibromyalgia Survey Criteria (FSC)^[^ ^25^ ^]^ and the Douleur Neuropathique 4 (DN4).^[^ ^26^ ^]^

### Longitudinal Pain Phenotypes—Douleur Neuropathique 4 (DN4)

The DN4 was a seven‐item questionnaire designed to assess neuropathic pain. In the UK Biobank study, an abbreviated version was used, omitting the clinical examination assessment. Scores range from 0 to 7, with a score of ≥3 indicating the presence of neuropathic pain. Although primarily used to diagnose neuropathic pain, the DN4 may also reflect central sensitisation, a key feature of nociplastic pain.^[^ ^27^ ^]^

### Longitudinal Pain Phenotypes—Fibromyalgia Survey Criteria (FSC)

The FSC was based on the 2016 ACR Fibromyalgia Survey Criteria^[^ ^25^ ^]^ and consists of two components: the Widespread Pain Index (WPI), which measures pain across 19 body areas (score 0–19), and the Symptom Severity Scale (SSS), which assesses somatic symptoms including fatigue, sleep disturbances, and cognitive difficulties (score 0–12). The total score (range 0–31) was calculated by combining the WPI and SSS scores.

The WPI was used to assess the severity of nociplastic pain, while the DN4 helped to identify both neuropathic pain and potential central sensitisation, providing a comprehensive evaluation of different pain types.

Based on these criteria, participants were classified into four distinct pain categories:

1. Nociceptive pain was defined for participants with chronic pain but no neuropathic characteristics (DN4 score = 0) and an FSC score <3, indicating a low likelihood of nociplastic pain. Participants without chronic pain were classified as having no nociceptive pain.
2. Neuropathic pain was defined for participants with chronic pain and a DN4 score ≥3, suggesting the presence of neuropathic pain. Participants with an FSC score ≥3 were excluded to reduce the likelihood of including those with possible nociplastic or fibromyalgia‐related pain.
3. Nociplastic pain was defined for participants exhibiting DN4 ≥3 or a total WPI and SSS score >12. Notably, universally accepted diagnostic criteria for nociplastic pain remain absent. Fibromyalgia, widely recognized as the prototypical manifestation of nociplastic pain, highlights the utility of the FSC score as an effective marker of symptom severity. In addition, the FSC score may reliably reflect the burden of nociplastic pain.^[^ ^28^ ^]^ Accordingly, an FSC score ≥12 serves as a practical threshold for identifying individuals likely to experience nociplastic pain as the predominant driver of their chronic pain.
4. Fibromyalgia was identified either through self‐reported diagnoses from baseline or the 2019 questionnaires or via general practitioner records. Additionally, participants with a WPI and SSS combined score >12 were classified as having fibromyalgia.^[^ ^28^ ^]^

### Longitudinal Pain Phenotypes—Online UK Biobank in 2019 – Pain Sites

Participants were inquired about experiencing pain or discomfort, either constantly or intermittently, for a duration exceeding three months. They were then requested to specify the affected sites, which included the head, face, neck, shoulders, chest, stomach, abdomen, back, hip, legs, knees, feet, arms, hands, and widespread body pain.

### Longitudinal Pain Phenotypes—New Onset Pain

Participants who did not report any chronic pain at baseline but reported chronic pain at the follow‐up visit were defined as having the onset of pain.

### Longitudinal Pain Phenotypes—Spreading Chronic Pain

The spreading of chronic pain was assessed by monitoring changes in the number of chronic pain sites. An increase in the number of pain sites at the follow‐up visit compared to the baseline visit was defined as pain spread.

### Longitudinal Pain Phenotypes—Recovery of Chronic Pain

Recovery of chronic pain was defined as a decrease in the number of pain sites at the follow‐up visit compared to the baseline visit.

### Longitudinal Pain Phenotypes—Duration of Pain

Participants reporting chronic pain were asked to specify the duration of their discomfort through the online questionnaire, selecting from the following categories: 3–12 months, 1–5 years, or more than 5 years.

### Longitudinal Pain Phenotypes—Pain Rating in the Last 24 H

Participants were also asked to rate their pain intensity at each reported chronic pain site over the preceding 24 h using a numeric rating scale of 0 to 10, where 0 represented no pain and 10 indicated the worst possible pain.

### Longitudinal Pain Phenotypes—Worst Pain Rating

Participants were prompted to rate the most severe pain they had experienced in the last 24 h at the chronic pain site that bothered them the most, again on a numeric rating scale from 0 (no pain) to 10 (the worst imaginable pain). or widespread pain, a separate questionnaire (“All‐over body pain in the last three months and rating of pain”) was used. The ratings from specific body sites and the overall widespread pain were combined for analysis.

### Longitudinal Pain Phenotypes—Pain Interference

The impact of pain on daily functioning was evaluated across seven domains: general activity, mood, walking ability, normal work, relationships with others, sleep, and enjoyment of life. Each domain was rated on a scale of 0 to 10, where 0 indicated no interference and 10 signified complete interference. A total pain interference score was derived by summing the individual scores for these seven items.

### Longitudinal Pain Phenotypes—Symptom Severity

Participants were asked to report the severity of three symptoms—fatigue, sleep quality, and cognitive symptoms—over the past week, as measured by the fibromyalgia symptom severity scale. Each symptom was rated on a scale from 0 (no problem) to 3 (severe, pervasive, continuous, and life‐disrupting issues). The total symptom severity score was calculated by summing the scores for fatigue, sleep quality, and cognitive symptoms.

### Longitudinal Pain Phenotypes—Online UK Biobank in 2022‐ Pain sites

In 2022, the UK Biobank conducted an online survey to assess pain at various anatomical sites among participants. Respondents were asked to identify current pain in specific areas, including the neck, back, chest, abdomen, legs, muscles, and joints. Additionally, participants provided information on the duration of the pain and the degree to which each site of pain impacted their daily lives. Pain persisting for more than twelve weeks was classified as chronic pain.

The onset of chronic pain, spreading chronic pain, and recovery of chronic pain in the follow‐up questionnaire 2022 were also defined, followed the phenotype at the follow‐up questionnaire 2019.

### Statistical Analyses—Proteome‐Wide Association Study

To identify protein biomarkers associated with diverse pain phenotypes, a proteome‐wide association study (PWAS) was conducted across 2920 plasma proteins. Logistic regression models were fitted for each of the following phenotypes: overall pain, head and face pain, abdominal pain, musculoskeletal pain, and widespread pain. Pain‐free individuals served as controls for each analysis. Protein levels were inverse rank‐normalized and standardized prior to modeling. Covariates included age, sex, sleeplessness, feeling fed‐up, tiredness, mood, stressful life events, and BMI. Statistical significance was defined using a Bonferroni threshold (P < 0.05 / 2920).

### Statistical Analyses—Functional Enrichment Analysis

Proteins significantly associated with widespread chronic pain were subjected to Gene Ontology (GO), KEGG, and Reactome pathway enrichment using clusterProfiler and ReactomePA in R. All quantified plasma proteins (n  =  2920) were used as the background set. Pathways with FDR‑adjusted P < 0.05 were considered significant, and the top enriched GO terms and KEGG/Reactome pathways were visualized by –log10(P‑adjusted).

### Statistical Analyses—Proteomic Score

To derive predictive signatures of widespread chronic pain, a two‐step machine learning framework leveraging nested cross‐validation (Figure S3, Supporting Information) was implemented. First, comprehensive proteomic score models (C‐ProtS) were developed using XGBoost, a scalable and efficient gradient boosting algorithm, trained on all proteins selected through internal feature filtering. Second, simple proteomic score models (S‐ProtS) were constructed by restricting the input to a SHAP‐informed subset of top‐ranked proteins contributing most to model prediction. XGBoost was a high‐performance machine learning algorithm designed for efficiency, flexibility, and portability, utilizing parallel tree boosting to develop the protein‐profiling model.^[^ ^29^ ^]^

### Statistical Analyses—Feature Selection

Feature selection was embedded within a nested cross‐validation framework to ensure unbiased variable selection and prevent information leakage. In each training fold of the inner loop, LASSO (least absolute shrinkage and selection operator) regression was applied to identify a subset of proteins most predictive of widespread chronic pain. The LASSO penalty encourages sparsity by shrinking less informative coefficients to zero, enabling data‐driven selection of relevant features within each fold.

### Statistical Analyses—Proteomic Score Development

A series of XGBOOST models was developed to assess the potential of plasma proteomics as a single‐domain assay for predicting widespread chronic pain. For chronic widespread pain, a separate XGBOOST model was constructed using nested cross‐validation (5 outer folds and 10 inner folds), a technique employed to minimize the risk of random error caused by data splitting. The predicted risk values from these models were designated as proteomic scores (comprehensive proteomic scores, C‐ProtS). To interpret model outputs and identify the most influential proteins, SHAP (SHapley Additive exPlanations) values based on the final XGBoost model were computed, which was retrained on the full dataset using the optimal hyperparameters selected through nested cross‐validation. SHAP values were derived from cooperative game theory and provide a unified measure to explain the contribution of each feature—in this case, each protein—to the model's prediction. A positive SHAP value indicates that the protein increases the predicted risk for widespread chronic pain, while a negative SHAP value suggests the protein decreases the predicted risk. The top 15 proteins were ranked based on their absolute SHAP values, indicating their relative importance in the model. SHAP plots were also generated to visually interpret the impact of each protein on the model's predictions, allowing for a clear understanding of how specific proteins influence the risk predictions for widespread chronic pain. Nested cross‑validation was used to mitigate overfitting and provide an internal estimate of model performance, although no external test set was available for independent evaluation.

To construct a simplified and interpretable model, all proteins were ranked by their SHAP values and sequentially added them to a new XGBoost model in descending order of importance. The process was terminated once the model's AUC reached 90% of that achieved by the full proteomic model (C‐ProtS). The resulting subset of proteins defined the simple model (simple proteomic score, S‐ProtS), which was developed using the same nested cross‐validation strategy.

### Statistical Analyses—Model for Pain Prediction

XGBOOST models were lifted, incorporating different sets of clinical predictors (Figure S4, Supporting Information). The model‐based based was developed on six predictors identified from a previous study, utilizing nested cross‐validation. Additionally, models combining these six clinical predictors were created with the proteomic score to evaluate their additive predictive value. Model performance was assessed using AUC, ranging from 0.5 (non‐informative) to 1 (perfect discrimination). Calibration plots were employed to visualize the agreement between predicted risks and observed pain rates, while net benefit curves illustrated the additive predictive value of ProRS alongside different clinical predictor sets.

Importantly, model predictions were derived from the test folds across each outer loop of the nested cross‐validation, ensuring unbiased performance estimates by avoiding information leakage. Key predictive metrics, including the area under the Receiver Operating Characteristic (ROC) curve (AUC), were reported. Calibration was evaluated using calibration plots and quantified through the calibration slope statistic, with a slope of 1 indicating optimal overall calibration. Net benefit curves were also generated to evaluate the incremental clinical value of incorporating the proteomic risk score into existing clinical models. All models underwent development and evaluation through nested cross‐validation, with continuous variables standardised and categorical variables one‐hot encoded.

### Statistical Analyses—Prospective Association of the Proteomic Score

To evaluate the prognostic association of the widespread chronic pain‐specific proteomic score, Poisson regression was employed to estimate the relationship between the proteomic score (quantile 5 vs quantile 1) and nociceptive pain, neuropathic pain, nociplastic pain, fibromyalgia, onset of chronic pain, pain spread, and pain recovery. For onset pain, the analysis focused on participants without any chronic pain at baseline who developed onset pain by the follow‐up visit. All models were adjusted for age, gender, sleeplessness, feelings of being fed‐up, tiredness, mood, stressful life events, and BMI.

### Statistical Analyses—Causal Relevance of the Proteins (Mendelian Randomization Analysis)

To systematically prioritize protein biomarkers implicated in widespread chronic pain, machine learning–based feature importance (proteins with non‐zero SHAP values) and conventional association analyses were integrated. To explore the causal relationship between integrated proteins and pain, a two‐sample Mendelian randomization (MR) analysis was conducted. Selection of genetic instruments: Instrumental variables were derived from two large‐scale genome‐wide association studies (GWAS): the UK Biobank Pharma Proteomics Project (UKB‐PPP). Due to the moderate heritability of circulating protein levels and limited sample sizes in current proteomic GWAS datasets, relatively few proteins possess genome‐wide significant instruments (p < 5 × 10^−8^). To enable proteome‐wide coverage and avoid restricting the analysis to only highly heritable proteins, a relaxed significance threshold of p < 5 × 10^−5^ was adopted for selecting genetic instruments. This threshold has been commonly used in proteomic MR studies to ensure adequate instrument availability. Cis‐SNPs MR Analysis: Cis‐SNPs associated with the top shared plasma proteins at genome‐wide significance (p < 5 × 10^−5^) and LD (R^2^ < 0.1) were utilized as instrumental variables. Cis‐SNPs were defined as those located within 5 Mb of the gene encoding the protein, with LD estimated based on the 1000 Genomes European panel. Furthermore, F‐statistics > 10 were applied to ensure adequate instrumental strength associated with exposure traits.

### Statistical Analyses—Protein Source

Genetic instruments for protein levels were obtained from the UK Biobank Pharma Proteomics Project, an external large‐scale proteomic GWAS that profiled 2923 plasma proteins in ≈54000 UK Biobank participants using the Olink platform.^[^ ^30^ ^]^

### Statistical Analyses—Pain Sources

To avoid potential bias arising from population diversity, the genetic background was restricted in drug target MR analysis to participants of European descent. Three large‐scale genome‐wide association studies (GWAS) of chronic pain was employed. The primary MR analysis was based on a meta‐analysis of chronic widespread pain across six population‐based European cohorts (14 177 cases and 28 903 controls), excluding UK Biobank: the Rotterdam Study I, II, and III (Netherlands), TwinsUK and the English Longitudinal Study of Ageing (UK), and the Nord‐Trøndelag Health Survey (Norway).^[^ ^31^ ^]^ For independent validation, two UK Biobank‐derived phenotypes was used: (1) multisite chronic pain, comprising 73082 cases and 105474 controls,^[^ ^32^ ^]^ and (2) chronic widespread musculoskeletal pain defined through self‐reported and ICD‐coded diagnoses (6914 cases and 223 606 controls).^[^ ^31^ ^]^ This three‐source design enabled robust causal inference and cross‐cohort replication.

### Statistical Analyses—Sensitivity Analysis

The Steiger test was employed to assess potential reverse causality in the MR analysis. Additionally, the bidirectional Mendelian randomization approach was used to explore the reverse causal relationship between multisite chronic pain and specific proteins. Associations between cis‐pQTLs and outcomes were calculated as beta with corresponding confidence intervals (CIs) using the random‐effects inverse‐variance weighted (IVW) method. Furthermore, MR‐PRESSO was utilized to detect outlier genetic variants. The MR‐Egger intercept test was conducted to evaluate horizontal pleiotropy, and Cochrane's Q value was calculated to assess heterogeneity in IVW estimators, with P < 0.05 indicating evidence of heterogeneity.

### Statistical Analyses—Colocalization Analysis

Colocalization analysis was performed to determine whether the observed associations between the top shared proteins and multisite chronic pain were driven by linkage disequilibrium. For each locus, a Bayesian framework was applied to evaluate support for five mutually exclusive hypotheses: (1) no association with either trait; (2) association with trait 1 only; (3) association with trait 2 only; (4) both traits were associated but have distinct causal variants; and (5) both traits were associated and share the same causal variant. The analysis yielded posterior probabilities for each hypothesis (H0, H1, H2, H3, and H4). Prior probabilities were assigned as follows: the SNP being associated with trait 1 only (p1) was set at 1 × 10^−4^; the SNP being associated with trait 2 only (p2) at 1 × 10^−4^; and the SNP being associated with both traits (p12) at 1 × 10^−5^. Strong evidence of colocalization was defined as a posterior probability for shared causal variants (PH4) ≥ 0.8, while medium evidence was indicated by 0.5 < PH4 < 0.8.

### Statistical Analyses—Prospective Association of the Top Proteins

Among the 434 proteins tested, 18 showed significant causal associations with widespread chronic pain in MR analyses. Poisson regression was employed to estimate the prognostic association between these top proteins and pain mechanism classification (nociceptive pain, neuropathic pain, nociplastic pain, and fibromyalgia), new onset chronic pain, pain spread, and pain recovery (quantile 5 vs quantile 1). The analysis was adjusted for variables including age, gender, sleeplessness, feeling fed‐up, tiredness, mood, stressful life events, and BMI.

To explore dose‐response relationships between the top proteins and pain outcomes, restricted cubic spline (RCS) regression was used, with the same multivariable adjustments. Nonlinearity was tested using the likelihood ratio test, and the number of nodes was selected based on the lowest Akaike Information Criterion (AIC) value. The same analytical approach was applied to the other 18 proteins.

### Statistical Analyses—Protein‐Protein Interaction (PPI)

Protein‐protein interaction (PPI) analysis was performed for the 18 causal proteins identified in our models using the STRING database (https://string‐db.org/).^[^ ^33^ ^]^ STRING offers comprehensive interaction networks derived from experimental data, computational predictions, and known biological pathways. This analysis enabled us to examine potential interactions between the top proteins and to identify biologically relevant networks that may be involved in the pathogenesis of widespread chronic pain.

### Statistical Analyses—Drug Repurposing

To explore the potential for drug repurposing, the Open Targets platform^[^ ^34^ ^]^ was searched for the 18 proteins identified in our analysis. Open Targets integrates data from multiple sources, including genetic associations, known drug interactions, and disease pathways, to evaluate the therapeutic potential of various proteins. This search enabled us to determine whether any of the top proteins were associated with existing drugs that could be repurposed for chronic pain treatment. By identifying proteins already targeted by approved or investigational drugs, opportunities were aimed to uncover for repurposing these drugs to address widespread chronic pain. This approach may offer faster and more cost‐effective therapeutic options by leveraging drugs that have already undergone substantial testing for safety and efficacy in other conditions.

All modeling and statistical analyses were performed using R software (V4.3.0). Missing value imputation was conducted using the missRanger package. The XGBOOST and nested cross‐validation were implemented with the nestedcv package, with calibration curves generated by the CalibrationCurve package and net benefit curves by the rmda package. The two‐sample Mendelian randomization analysis was conducted using the TwosampleMR package. Colocalization analysis was carried out with the *coloc* package.

## Conflict of Interest

The authors declare no conflict of interest.

## Supporting information



Supporting Information

## Data Availability

The data that support the findings of this study are available on request from the corresponding author.
