# Supplementary material for: Diagnosis, Prognosis, and Drug Target Discovery for Chronic Widespread Pain: A Large Proteogenomic Study
Source: Adv Sci (Weinh). 2025 Sep 30;12(47):e07691. doi: 10.1002/advs.202507691 (PMC12713070; doi:10.1002/advs.202507691)
Supplement: Supplementary file 1 — Supporting Information [file ADVS-12-e07691-s001.docx]

**Supplementary files**

| Table S1. Definitions of Binary Clinical Predictors and the comparison of the participants with proteins and without proteins | | | | |
| --- | --- | --- | --- | --- |
| **Variables** |  | participants without proteins | participants with proteins |  |
| **Number** |  | 449337 | 53029 |  |
| **Age** |  | 56.50 (8.08) | 56.81 (8.21) | <0.001 |
| **Gender** | Female | 244708 (54.5) | 28590 (53.9) | 0.017 |
|  | Male | 204629 (45.5) | 24439 (46.1) |  |
| **Sleeplessness** | Rarely or sometimes | 321741 (71.8) | 37760 (71.4) | 0.027 |
|  | Usually | 126210 (28.2) | 15150 (28.6) |  |
| **Feeling ‘fed-up’** | Rarely or sometimes | 260134 (59.4) | 30589 (59.1) | 0.215 |
|  | Usually | 177912 (40.6) | 21168 (40.9) |  |
| **Tiredness** | Not at all or several days | 378582 (87.2) | 44367 (86.7) | <0.001 |
|  | More than half the days | 55340 (12.8) | 6825 (13.3) |  |
| **Mood (Nerves, anxiety, tension, or depression)** | No | 290040 (65.4) | 34006 (64.9) | 0.031 |
|  | Yes | 153429 (34.6) | 18370 (35.1) |  |
| **Stressful life events** | No | 248129 (55.6) | 28790 (54.7) | <0.001 |
|  | Yes | 198067 (44.4) | 23845 (45.3) |  |
| **Body mass index >30** | <= 30 | 337327 (75.6) | 39804 (75.4) | 0.542 |
|  | > 30 | 109162 (24.4) | 12966 (24.6) |  |

| Table S2. Demographic information for population. | | | | |
| --- | --- | --- | --- | --- |
|  | Female | Male | | Overall |
|  | (N=16215) | (N=13039) | | (N=29254) |
| **Age** |  |  | |  |
| Mean (SD) | 55.9 (8.02) | 55.7 (8.22) | | 55.8 (8.11) |
| Median [Min, Max] | 57.0 [40.0, 70.0] | 57.0 [39.0, 70.0] | | 57.0 [39.0, 70.0] |
| **Sleeplessness** |  |  | |  |
| Rarely or sometimes | 10905 (67.3%) | 9925 (76.1%) | | 20830 (71.2%) |
| Usually | 5310 (32.7%) | 3114 (23.9%) | | 8424 (28.8%) |
| **Feeling ‘fed-up’** |  |  | |  |
| No | 9273 (57.2%) | 8071 (61.9%) | | 17344 (59.3%) |
| Yes | 6942 (42.8%) | 4968 (38.1%) | | 11910 (40.7%) |
| **Tiredness** |  |  | |  |
| Not at all or several days | 13977 (86.2%) | 11592 (88.9%) | | 25569 (87.4%) |
| More than half the days | 2238 (13.8%) | 1447 (11.1%) | | 3685 (12.6%) |
| **Mood** |  |  | |  |
| No | 9445 (58.2%) | 9483 (72.7%) | | 18928 (64.7%) |
| Yes | 6770 (41.8%) | 3556 (27.3%) | | 10326 (35.3%) |
| **Stressful life events** |  |  | |  |
| No | 8873 (54.7%) | 7372 (56.5%) | | 16245 (55.5%) |
| Yes | 7342 (45.3%) | 5667 (43.5%) | | 13009 (44.5%) |
| **Body mass index >30** |  |  | |  |
| <= 30 | 12427 (76.6%) | 9788 (75.1%) | | 22215 (75.9%) |
| > 30 | 3788 (23.4%) | 3251 (24.9%) | | 7039 (24.1%) |
| **Chronic pain status** |  |  | |  |
| No | 7444 (45.9%) | 6524 (50.0%) | | 13968 (47.7%) |
| Yes | 8771 (54.1%) | 6515 (50.0%) | | 15286 (52.3%) |
| **Pain types^a^** |  |  | |  |
| Head and face pain | 2413 | 1024 | | 3437 |
| Abdominal pain | 1041 | 601 | | 1642 |
| Musculoskeletal pain | 7246 | 5777 | | 13023 |
| Widespread pain | 276 | 183 | | 459 |
| Number of pain site |  |  | |  |
| 0 | 7401 (45.6%) | 6480 (49.7%) | | 13881 (47.5%) |
| 1 | 4520 (27.9%) | 3713 (28.5%) | | 8233 (28.1%) |
| 2 | 2226 (13.7%) | 1572 (12.1%) | | 3798 (13.0%) |
| 3 | 1063 (6.6%) | 706 (5.4%) | | 1769 (6.0%) |
| 4 | 475 (2.9%) | 265 (2.0%) | | 740 (2.5%) |
| 5 | 158 (1.0%) | 61 (0.5%) | | 219 (0.7%) |
| 6 | 46 (0.3%) | 13 (0.1%) | | 59 (0.2%) |
| 7 | 7 (0.0%) | 2 (0.0%) | | 9 (0.0%) |
| 8 (Widespread pain) | 276 (1.7%) | 183 (1.4%) | | 459 (1.6%) |
| Pain mechanism classification in 2019 | | | total number of participants is 8225 | |
| **Nociceptive_pain** |  |  | |  |
| 0 | 2865 | 2379 | | 5244 |
| 1 | 283 | 282 | | 571 |
| **Neuropathic_pain** |  |  | |  |
| 0 | 2865 | 2379 | | 5244 |
| 1 | 35 | 40 | | 75 |
| **Nociplastic_pain** |  |  | |  |
| 0 | 2865 | 2379 | | 5244 |
| 1 | 2660 | 1493 | | 4153 |
| **Fibromyalgia** |  |  | |  |
| 0 | 2865 | 2379 | | 5244 |
| 1 | 569 | 148 | | 717 |
| Notes: a, the percentage of pain types represents the proportion of specific pain relative to the total chronic pain. | | | | |

| Table S3. Number of proteins associated chronic pain and acute pain | | | |
| --- | --- | --- | --- |
| Pain type | Chronic pain specific | Acute pain specific | Associated with Both acute pain and chronic pain |
| Overall pain | 214 | 7 | 40 |
| Head and Face Pain | 59 | 15 | 14 |
| Abdominal Pain | 140 | 20 | 27 |
| Musculoskeletal Pain | 237 | 1 | 8 |
| Widespread pain | 245 | 16 | 11 |

| Table S4. Number of proteins significant associated with specific pain types and its overlap of the proteins. | | |
| --- | --- | --- |
| Pain type | Number of proteins | Proportion |
| Chronic pain status | 254 | 15.75% |
| Head and Face Pain | 73 | 19.18% |
| Abdominal Pain | 167 | 16.17% |
| Musculoskeletal Pain | 245 | 3.27% |
| Widespread pain | 256 | 4.30% |

Table S5. DeLong’s Test for Pairwise AUC Comparisons Between Models .

| Model_A | Model_B | AUC_A_ci | AUC_B_ci | p_value |
| --- | --- | --- | --- | --- |
| **C-ProtS** | **Clinical** | 0.801 (0.780,0.822) | 0.791 (0.767,0.814) | 0.533 |
| **C-ProtS** | **S-ProtS** | 0.801 (0.780,0.822) | 0.723 (0.700,0.746) | 9.55E-07 |
| **C-ProtS** | **C-ProtS+Clinical** | 0.801 (0.780,0.822) | 0.880 (0.864,0.897) | 4.03E-09 |
| **C-ProtS** | **S-ProtS+Clinical** | 0.801 (0.780,0.822) | 0.856 (0.838,0.874) | 9.17E-05 |
| **S-ProtS** | **Clinical** | 0.723 (0.700,0.746) | 0.791 (0.767,0.814) | 5.83E-05 |
| **Clinical** | **S-ProtS** | 0.791 (0.767,0.814) | 0.856 (0.838,0.874) | 1.84E-05 |
| **Clinical** | **C-ProtS+Clinical** | 0.791 (0.767,0.814) | 0.880 (0.864,0.897) | 1.05E-09 |

Table S6. Proteomics-based drug repurposing

| protein | drugName | type | mechanismOfAction | actionType | diseaseName | phase | status |
| --- | --- | --- | --- | --- | --- | --- | --- |
| CA14 | [SULTHIAME](https://platform.opentargets.org/drug/CHEMBL328560) | Small molecule | Carbonic anhydrase inhibitor | Inhibitor | [epilepsy](https://platform.opentargets.org/disease/EFO_0000474) | 4 | N/A |
| CA14 | [SULTHIAME](https://platform.opentargets.org/drug/CHEMBL328560) | Small molecule | Carbonic anhydrase inhibitor | Inhibitor | [epilepsy](https://platform.opentargets.org/disease/EFO_0000474) | 3 | Terminated |
| CA14 | [SULTHIAME](https://platform.opentargets.org/drug/CHEMBL328560) | Small molecule | Carbonic anhydrase inhibitor | Inhibitor | [obstructive sleep apnea](https://platform.opentargets.org/disease/EFO_0003918) | 2 | Completed |
| CA14 | [SULTHIAME](https://platform.opentargets.org/drug/CHEMBL328560) | Small molecule | Carbonic anhydrase inhibitor | Inhibitor | [epilepsy](https://platform.opentargets.org/disease/EFO_0000474) | 1 | Completed |
| DPEP1 | [CILASTATIN SODIUM](https://platform.opentargets.org/drug/CHEMBL1201057) | Small molecule | Renal dipeptidase inhibitor | Inhibitor | [endocarditis](https://platform.opentargets.org/disease/EFO_0000465) | 4 | N/A |
| DPEP1 | [CILASTATIN SODIUM](https://platform.opentargets.org/drug/CHEMBL1201057) | Small molecule | Renal dipeptidase inhibitor | Inhibitor | [infection](https://platform.opentargets.org/disease/EFO_0000544) | 4 | N/A |
| DPEP1 | [CILASTATIN SODIUM](https://platform.opentargets.org/drug/CHEMBL1201057) | Small molecule | Renal dipeptidase inhibitor | Inhibitor | [infection](https://platform.opentargets.org/disease/EFO_0000544) | 4 | Completed |
| DPEP1 | [CILASTATIN SODIUM](https://platform.opentargets.org/drug/CHEMBL1201057) | Small molecule | Renal dipeptidase inhibitor | Inhibitor | [urinary tract infection](https://platform.opentargets.org/disease/EFO_0003103) | 4 | N/A |
| DPEP1 | [CILASTATIN SODIUM](https://platform.opentargets.org/drug/CHEMBL1201057) | Small molecule | Renal dipeptidase inhibitor | Inhibitor | [pneumonia](https://platform.opentargets.org/disease/EFO_0003106) | 4 | N/A |
| DPEP1 | [CILASTATIN SODIUM](https://platform.opentargets.org/drug/CHEMBL1201057) | Small molecule | Renal dipeptidase inhibitor | Inhibitor | [pneumonia](https://platform.opentargets.org/disease/EFO_0003106) | 4 | Completed |
| DPEP1 | [CILASTATIN SODIUM](https://platform.opentargets.org/drug/CHEMBL1201057) | Small molecule | Renal dipeptidase inhibitor | Inhibitor | [Sepsis](https://platform.opentargets.org/disease/HP_0100806) | 4 | N/A |
| DPEP1 | [CILASTATIN](https://platform.opentargets.org/drug/CHEMBL766) | Small molecule | Renal dipeptidase inhibitor | Inhibitor | [bacterial disease](https://platform.opentargets.org/disease/EFO_0000771) | 3 | Completed |
| DPEP1 | [CILASTATIN](https://platform.opentargets.org/drug/CHEMBL766) | Small molecule | Renal dipeptidase inhibitor | Inhibitor | [urinary tract infection](https://platform.opentargets.org/disease/EFO_0003103) | 3 | Not yet recruiting |
| DPEP1 | [CILASTATIN](https://platform.opentargets.org/drug/CHEMBL766) | Small molecule | Renal dipeptidase inhibitor | Inhibitor | [urinary tract infection](https://platform.opentargets.org/disease/EFO_0003103) | 3 | Recruiting |
| DPEP1 | [CILASTATIN](https://platform.opentargets.org/drug/CHEMBL766) | Small molecule | Renal dipeptidase inhibitor | Inhibitor | [pyelonephritis](https://platform.opentargets.org/disease/EFO_1001141) | 3 | Not yet recruiting |
| DPEP1 | [CILASTATIN](https://platform.opentargets.org/drug/CHEMBL766) | Small molecule | Renal dipeptidase inhibitor | Inhibitor | [bacterial pneumonia](https://platform.opentargets.org/disease/EFO_1001272) | 3 | Completed |
| DPEP1 | [CILASTATIN](https://platform.opentargets.org/drug/CHEMBL766) | Small molecule | Renal dipeptidase inhibitor | Inhibitor | [bacterial pneumonia](https://platform.opentargets.org/disease/EFO_1001272) | 3 | Recruiting |
| DPEP1 | [CILASTATIN](https://platform.opentargets.org/drug/CHEMBL766) | Small molecule | Renal dipeptidase inhibitor | Inhibitor | [ventilator-associated pneumonia](https://platform.opentargets.org/disease/EFO_1001865) | 3 | Terminated |
| DPEP1 | [CILASTATIN](https://platform.opentargets.org/drug/CHEMBL766) | Small molecule | Renal dipeptidase inhibitor | Inhibitor | [acute pyelonephritis](https://platform.opentargets.org/disease/MONDO_0003529) | 3 | Recruiting |
| DPEP1 | [CILASTATIN](https://platform.opentargets.org/drug/CHEMBL766) | Small molecule | Renal dipeptidase inhibitor | Inhibitor | [infection](https://platform.opentargets.org/disease/EFO_0000544) | 2 | Completed |
| DPEP1 | [CILASTATIN](https://platform.opentargets.org/drug/CHEMBL766) | Small molecule | Renal dipeptidase inhibitor | Inhibitor | [infection](https://platform.opentargets.org/disease/EFO_0000544) | 2 | Terminated |
| DPEP1 | [CILASTATIN](https://platform.opentargets.org/drug/CHEMBL766) | Small molecule | Renal dipeptidase inhibitor | Inhibitor | [urinary tract infection](https://platform.opentargets.org/disease/EFO_0003103) | 2 | Completed |
| DPEP1 | [CILASTATIN](https://platform.opentargets.org/drug/CHEMBL766) | Small molecule | Renal dipeptidase inhibitor | Inhibitor | [pyelonephritis](https://platform.opentargets.org/disease/EFO_1001141) | 2 | Completed |
| DPEP1 | [CILASTATIN](https://platform.opentargets.org/drug/CHEMBL766) | Small molecule | Renal dipeptidase inhibitor | Inhibitor | [bacterial pneumonia](https://platform.opentargets.org/disease/EFO_1001272) | 2 | Terminated |
| DPEP1 | [CILASTATIN](https://platform.opentargets.org/drug/CHEMBL766) | Small molecule | Renal dipeptidase inhibitor | Inhibitor | [acute pyelonephritis](https://platform.opentargets.org/disease/MONDO_0003529) | 2 | Completed |
| DPEP1 | [CILASTATIN](https://platform.opentargets.org/drug/CHEMBL766) | Small molecule | Renal dipeptidase inhibitor | Inhibitor | [hematopoietic and lymphoid cell neoplasm](https://platform.opentargets.org/disease/MONDO_0044881) | 2 | Completed |
| DPEP1 | [CILASTATIN](https://platform.opentargets.org/drug/CHEMBL766) | Small molecule | Renal dipeptidase inhibitor | Inhibitor | [infection](https://platform.opentargets.org/disease/EFO_0000544) | 1 | Completed |
| DPEP1 | [CILASTATIN](https://platform.opentargets.org/drug/CHEMBL766) | Small molecule | Renal dipeptidase inhibitor | Inhibitor | [bacterial disease](https://platform.opentargets.org/disease/EFO_0000771) | 1 | Completed |
| DPEP1 | [CILASTATIN](https://platform.opentargets.org/drug/CHEMBL766) | Small molecule | Renal dipeptidase inhibitor | Inhibitor | [Sepsis](https://platform.opentargets.org/disease/HP_0100806) | 1 | Completed |
| DPEP1 | [CILASTATIN](https://platform.opentargets.org/drug/CHEMBL766) | Small molecule | Renal dipeptidase inhibitor | Inhibitor | [osteomyelitis](https://platform.opentargets.org/disease/EFO_0003102) | 1 | Terminated |
| LGALS3 | [BELAPECTIN](https://platform.opentargets.org/drug/CHEMBL4297577) | Unknown | Galectin-3 inhibitor | Inhibitor | [portal hypertension](https://platform.opentargets.org/disease/EFO_0000666) | 2 | Completed |
| LGALS3 | [BELAPECTIN](https://platform.opentargets.org/drug/CHEMBL4297577) | Unknown | Galectin-3 inhibitor | Inhibitor | [psoriasis](https://platform.opentargets.org/disease/EFO_0000676) | 2 | Completed |
| LGALS3 | [OLITIGALTIN](https://platform.opentargets.org/drug/CHEMBL4297442) | Small molecule | Galectin-3 inhibitor | Inhibitor | [idiopathic pulmonary fibrosis](https://platform.opentargets.org/disease/EFO_0000768) | 2 | Completed |
| LGALS3 | [DAVANAT](https://platform.opentargets.org/drug/CHEMBL4297960) | Unknown | Galectin-3 inhibitor | Inhibitor | [bile duct carcinoma](https://platform.opentargets.org/disease/EFO_0005540) | 2 | Withdrawn |
| LGALS3 | [BELAPECTIN](https://platform.opentargets.org/drug/CHEMBL4297577) | Unknown | Galectin-3 inhibitor | Inhibitor | [non-alcoholic steatohepatitis](https://platform.opentargets.org/disease/EFO_1001249) | 2 | Completed |
| LGALS3 | [DAVANAT](https://platform.opentargets.org/drug/CHEMBL4297960) | Unknown | Galectin-3 inhibitor | Inhibitor | [colorectal cancer](https://platform.opentargets.org/disease/MONDO_0005575) | 2 | Withdrawn |
| LGALS3 | [DAVANAT](https://platform.opentargets.org/drug/CHEMBL4297960) | Unknown | Galectin-3 inhibitor | Inhibitor | [gallbladder cancer](https://platform.opentargets.org/disease/MONDO_0005411) | 2 | Withdrawn |
| LGALS3 | [BELAPECTIN](https://platform.opentargets.org/drug/CHEMBL4297577) | Unknown | Galectin-3 inhibitor | Inhibitor | [melanoma](https://platform.opentargets.org/disease/EFO_0000756) | 1 | Completed |
| LGALS3 | [OLITIGALTIN](https://platform.opentargets.org/drug/CHEMBL4297442) | Small molecule | Galectin-3 inhibitor | Inhibitor | [idiopathic pulmonary fibrosis](https://platform.opentargets.org/disease/EFO_0000768) | 1 | Completed |
| LGALS3 | [BELAPECTIN](https://platform.opentargets.org/drug/CHEMBL4297577) | Unknown | Galectin-3 inhibitor | Inhibitor | [liver disease](https://platform.opentargets.org/disease/EFO_0001421) | 1 | Completed |
| LGALS3 | [BELAPECTIN](https://platform.opentargets.org/drug/CHEMBL4297577) | Unknown | Galectin-3 inhibitor | Inhibitor | [metastatic melanoma](https://platform.opentargets.org/disease/EFO_0002617) | 1 | Completed |
| LGALS3 | [DAVANAT](https://platform.opentargets.org/drug/CHEMBL4297960) | Unknown | Galectin-3 inhibitor | Inhibitor | [metastatic melanoma](https://platform.opentargets.org/disease/EFO_0002617) | 1 | Terminated |
| LGALS3 | [BELAPECTIN](https://platform.opentargets.org/drug/CHEMBL4297577) | Unknown | Galectin-3 inhibitor | Inhibitor | [non-alcoholic fatty liver disease](https://platform.opentargets.org/disease/EFO_0003095) | 1 | Completed |
| LGALS3 | [OLITIGALTIN](https://platform.opentargets.org/drug/CHEMBL4297442) | Small molecule | Galectin-3 inhibitor | Inhibitor | [COVID-19](https://platform.opentargets.org/disease/MONDO_0100096) | 1 | Recruiting |
| TNF | ADALIMUMAB | Antibody | TNF-alpha inhibitor | Inhibitor | Crohn's disease | 4 |  |
| TNF | ADALIMUMAB | Antibody | TNF-alpha inhibitor | Inhibitor | Crohn's disease | 4 | Active, not recruiting |
| TNF | ADALIMUMAB | Antibody | TNF-alpha inhibitor | Inhibitor | Crohn's disease | 4 | Completed |
| TNF | ADALIMUMAB | Antibody | TNF-alpha inhibitor | Inhibitor | Crohn's disease | 4 | Not yet recruiting |
| TNF | ADALIMUMAB | Antibody | TNF-alpha inhibitor | Inhibitor | Crohn's disease | 4 | Recruiting |
| TNF | ADALIMUMAB | Antibody | TNF-alpha inhibitor | Inhibitor | Crohn's disease | 4 | Withdrawn |
| TNF | INFLIXIMAB | Antibody | TNF-alpha inhibitor | Inhibitor | Crohn's disease | 4 |  |
| TNF | INFLIXIMAB | Antibody | TNF-alpha inhibitor | Inhibitor | Crohn's disease | 4 | Active, not recruiting |
| TNF | INFLIXIMAB | Antibody | TNF-alpha inhibitor | Inhibitor | Crohn's disease | 4 | Unknown status |
| TNF | CERTOLIZUMAB PEGOL | Antibody | TNF-alpha inhibitor | Inhibitor | Crohn's disease | 4 |  |
| TNF | CERTOLIZUMAB PEGOL | Antibody | TNF-alpha inhibitor | Inhibitor | Crohn's disease | 4 | Terminated |
| TNF | ADALIMUMAB | Antibody | TNF-alpha inhibitor | Inhibitor | Crohn's disease | 4 | Enrolling by invitation |
| TNF | ADALIMUMAB | Antibody | TNF-alpha inhibitor | Inhibitor | Crohn's disease | 4 | Terminated |
| TNF | ADALIMUMAB | Antibody | TNF-alpha inhibitor | Inhibitor | Crohn's disease | 4 | Unknown status |
| TNF | INFLIXIMAB | Antibody | TNF-alpha inhibitor | Inhibitor | Crohn's disease | 4 | Completed |
| TNF | INFLIXIMAB | Antibody | TNF-alpha inhibitor | Inhibitor | Crohn's disease | 4 | Enrolling by invitation |
| TNF | INFLIXIMAB | Antibody | TNF-alpha inhibitor | Inhibitor | Crohn's disease | 4 | Not yet recruiting |
| TNF | INFLIXIMAB | Antibody | TNF-alpha inhibitor | Inhibitor | Crohn's disease | 4 | Recruiting |
| TNF | INFLIXIMAB | Antibody | TNF-alpha inhibitor | Inhibitor | Crohn's disease | 4 | Terminated |
| TNF | INFLIXIMAB | Antibody | TNF-alpha inhibitor | Inhibitor | Crohn's disease | 4 | Withdrawn |
| TNF | CERTOLIZUMAB PEGOL | Antibody | TNF-alpha inhibitor | Inhibitor | Crohn's disease | 4 | Unknown status |
| TNF | ETANERCEPT | Protein | TNF-alpha inhibitor | Inhibitor | immune system disease | 4 |  |
| TNF | INFLIXIMAB | Antibody | TNF-alpha inhibitor | Inhibitor | immune system disease | 4 |  |
| TNF | CERTOLIZUMAB PEGOL | Antibody | TNF-alpha inhibitor | Inhibitor | immune system disease | 4 |  |
| TNF | GOLIMUMAB | Antibody | TNF-alpha inhibitor | Inhibitor | immune system disease | 4 |  |
| TNF | ADALIMUMAB | Antibody | TNF-alpha inhibitor | Inhibitor | immune system disease | 4 |  |
| TNF | ETANERCEPT | Protein | TNF-alpha inhibitor | Inhibitor | psoriasis | 4 |  |
| TNF | ETANERCEPT | Protein | TNF-alpha inhibitor | Inhibitor | psoriasis | 4 | Completed |
| TNF | ETANERCEPT | Protein | TNF-alpha inhibitor | Inhibitor | psoriasis | 4 | Terminated |
| TNF | ETANERCEPT | Protein | TNF-alpha inhibitor | Inhibitor | psoriasis | 4 | Withdrawn |
| TNF | ADALIMUMAB | Antibody | TNF-alpha inhibitor | Inhibitor | psoriasis | 4 | Completed |
| TNF | INFLIXIMAB | Antibody | TNF-alpha inhibitor | Inhibitor | psoriasis | 4 |  |
| TNF | INFLIXIMAB | Antibody | TNF-alpha inhibitor | Inhibitor | psoriasis | 4 | Completed |
| TNF | ETANERCEPT | Protein | TNF-alpha inhibitor | Inhibitor | rheumatoid arthritis | 4 |  |
| TNF | ETANERCEPT | Protein | TNF-alpha inhibitor | Inhibitor | rheumatoid arthritis | 4 | Completed |
| TNF | ETANERCEPT | Protein | TNF-alpha inhibitor | Inhibitor | rheumatoid arthritis | 4 | Recruiting |
| TNF | ETANERCEPT | Protein | TNF-alpha inhibitor | Inhibitor | rheumatoid arthritis | 4 | Terminated |
| TNF | ETANERCEPT | Protein | TNF-alpha inhibitor | Inhibitor | rheumatoid arthritis | 4 | Unknown status |
| TNF | ADALIMUMAB | Antibody | TNF-alpha inhibitor | Inhibitor | rheumatoid arthritis | 4 |  |
| TNF | ADALIMUMAB | Antibody | TNF-alpha inhibitor | Inhibitor | rheumatoid arthritis | 4 | Active, not recruiting |
| TNF | ADALIMUMAB | Antibody | TNF-alpha inhibitor | Inhibitor | rheumatoid arthritis | 4 | Completed |
| TNF | ADALIMUMAB | Antibody | TNF-alpha inhibitor | Inhibitor | rheumatoid arthritis | 4 | Recruiting |
| TNF | ADALIMUMAB | Antibody | TNF-alpha inhibitor | Inhibitor | rheumatoid arthritis | 4 | Unknown status |
| TNF | INFLIXIMAB | Antibody | TNF-alpha inhibitor | Inhibitor | rheumatoid arthritis | 4 |  |
| TNF | INFLIXIMAB | Antibody | TNF-alpha inhibitor | Inhibitor | rheumatoid arthritis | 4 | Terminated |
| TNF | INFLIXIMAB | Antibody | TNF-alpha inhibitor | Inhibitor | rheumatoid arthritis | 4 | Unknown status |
| TNF | CERTOLIZUMAB PEGOL | Antibody | TNF-alpha inhibitor | Inhibitor | rheumatoid arthritis | 4 |  |
| TNF | GOLIMUMAB | Antibody | TNF-alpha inhibitor | Inhibitor | rheumatoid arthritis | 4 |  |
| TNF | GOLIMUMAB | Antibody | TNF-alpha inhibitor | Inhibitor | rheumatoid arthritis | 4 | Completed |
| TNF | GOLIMUMAB | Antibody | TNF-alpha inhibitor | Inhibitor | rheumatoid arthritis | 4 | Unknown status |
| TNF | ADALIMUMAB | Antibody | TNF-alpha inhibitor | Inhibitor | rheumatoid arthritis | 4 | Not yet recruiting |
| TNF | ADALIMUMAB | Antibody | TNF-alpha inhibitor | Inhibitor | rheumatoid arthritis | 4 | Terminated |
| TNF | INFLIXIMAB | Antibody | TNF-alpha inhibitor | Inhibitor | rheumatoid arthritis | 4 | Active, not recruiting |
| TNF | INFLIXIMAB | Antibody | TNF-alpha inhibitor | Inhibitor | rheumatoid arthritis | 4 | Completed |
| TNF | CERTOLIZUMAB PEGOL | Antibody | TNF-alpha inhibitor | Inhibitor | rheumatoid arthritis | 4 | Completed |
| TNF | CERTOLIZUMAB PEGOL | Antibody | TNF-alpha inhibitor | Inhibitor | rheumatoid arthritis | 4 | Terminated |
| TNF | CERTOLIZUMAB PEGOL | Antibody | TNF-alpha inhibitor | Inhibitor | rheumatoid arthritis | 4 | Unknown status |
| TNF | GOLIMUMAB | Antibody | TNF-alpha inhibitor | Inhibitor | rheumatoid arthritis | 4 | Active, not recruiting |
| TNF | GOLIMUMAB | Antibody | TNF-alpha inhibitor | Inhibitor | rheumatoid arthritis | 4 | Recruiting |
| TNF | GOLIMUMAB | Antibody | TNF-alpha inhibitor | Inhibitor | rheumatoid arthritis | 4 | Terminated |
| TNF | ETANERCEPT | Protein | TNF-alpha inhibitor | Inhibitor | psoriasis | 4 | Recruiting |
| TNF | ETANERCEPT | Protein | TNF-alpha inhibitor | Inhibitor | psoriasis | 4 | Unknown status |
| TNF | ADALIMUMAB | Antibody | TNF-alpha inhibitor | Inhibitor | psoriasis | 4 |  |
| TNF | ADALIMUMAB | Antibody | TNF-alpha inhibitor | Inhibitor | ulcerative colitis | 4 |  |
| TNF | ADALIMUMAB | Antibody | TNF-alpha inhibitor | Inhibitor | ulcerative colitis | 4 | Completed |
| TNF | ADALIMUMAB | Antibody | TNF-alpha inhibitor | Inhibitor | ulcerative colitis | 4 | Terminated |
| TNF | INFLIXIMAB | Antibody | TNF-alpha inhibitor | Inhibitor | ulcerative colitis | 4 | Terminated |
| TNF | GOLIMUMAB | Antibody | TNF-alpha inhibitor | Inhibitor | ulcerative colitis | 4 |  |
| TNF | GOLIMUMAB | Antibody | TNF-alpha inhibitor | Inhibitor | ulcerative colitis | 4 | Completed |
| TNF | ADALIMUMAB | Antibody | TNF-alpha inhibitor | Inhibitor | ulcerative colitis | 4 | Enrolling by invitation |
| TNF | ADALIMUMAB | Antibody | TNF-alpha inhibitor | Inhibitor | ulcerative colitis | 4 | Recruiting |
| TNF | ADALIMUMAB | Antibody | TNF-alpha inhibitor | Inhibitor | ulcerative colitis | 4 | Withdrawn |
| TNF | INFLIXIMAB | Antibody | TNF-alpha inhibitor | Inhibitor | ulcerative colitis | 4 |  |
| TNF | INFLIXIMAB | Antibody | TNF-alpha inhibitor | Inhibitor | ulcerative colitis | 4 | Active, not recruiting |
| TNF | INFLIXIMAB | Antibody | TNF-alpha inhibitor | Inhibitor | ulcerative colitis | 4 | Completed |
| TNF | INFLIXIMAB | Antibody | TNF-alpha inhibitor | Inhibitor | ulcerative colitis | 4 | Enrolling by invitation |
| TNF | INFLIXIMAB | Antibody | TNF-alpha inhibitor | Inhibitor | ulcerative colitis | 4 | Recruiting |
| TNF | INFLIXIMAB | Antibody | TNF-alpha inhibitor | Inhibitor | ulcerative colitis | 4 | Unknown status |
| TNF | GOLIMUMAB | Antibody | TNF-alpha inhibitor | Inhibitor | ulcerative colitis | 4 | Not yet recruiting |
| TNF | GOLIMUMAB | Antibody | TNF-alpha inhibitor | Inhibitor | ulcerative colitis | 4 | Recruiting |
| TNF | GOLIMUMAB | Antibody | TNF-alpha inhibitor | Inhibitor | ulcerative colitis | 4 | Unknown status |
| TNF | ETANERCEPT | Protein | TNF-alpha inhibitor | Inhibitor | rheumatoid arthritis | 4 | Active, not recruiting |
| TNF | ETANERCEPT | Protein | TNF-alpha inhibitor | Inhibitor | rheumatoid arthritis | 4 | Not yet recruiting |
| TNF | ETANERCEPT | Protein | TNF-alpha inhibitor | Inhibitor | rheumatoid arthritis | 4 | Withdrawn |
| TNF | ETANERCEPT | Protein | TNF-alpha inhibitor | Inhibitor | juvenile idiopathic arthritis | 4 | Unknown status |
| TNF | ADALIMUMAB | Antibody | TNF-alpha inhibitor | Inhibitor | juvenile idiopathic arthritis | 4 |  |
| TNF | ETANERCEPT | Protein | TNF-alpha inhibitor | Inhibitor | juvenile idiopathic arthritis | 4 |  |
| TNF | ETANERCEPT | Protein | TNF-alpha inhibitor | Inhibitor | juvenile idiopathic arthritis | 4 | Completed |
| TNF | ADALIMUMAB | Antibody | TNF-alpha inhibitor | Inhibitor | juvenile idiopathic arthritis | 4 | Active, not recruiting |
| TNF | ETANERCEPT | Protein | TNF-alpha inhibitor | Inhibitor | psoriatic arthritis | 4 |  |
| TNF | ETANERCEPT | Protein | TNF-alpha inhibitor | Inhibitor | psoriatic arthritis | 4 | Completed |
| TNF | ADALIMUMAB | Antibody | TNF-alpha inhibitor | Inhibitor | psoriatic arthritis | 4 |  |
| TNF | ADALIMUMAB | Antibody | TNF-alpha inhibitor | Inhibitor | psoriatic arthritis | 4 | Completed |
| TNF | INFLIXIMAB | Antibody | TNF-alpha inhibitor | Inhibitor | psoriatic arthritis | 4 |  |
| TNF | INFLIXIMAB | Antibody | TNF-alpha inhibitor | Inhibitor | psoriatic arthritis | 4 | Completed |
| TNF | CERTOLIZUMAB PEGOL | Antibody | TNF-alpha inhibitor | Inhibitor | psoriatic arthritis | 4 |  |
| TNF | GOLIMUMAB | Antibody | TNF-alpha inhibitor | Inhibitor | psoriatic arthritis | 4 |  |
| TNF | ETANERCEPT | Protein | TNF-alpha inhibitor | Inhibitor | psoriatic arthritis | 4 | Recruiting |
| TNF | ADALIMUMAB | Antibody | TNF-alpha inhibitor | Inhibitor | psoriatic arthritis | 4 | Recruiting |
| TNF | GOLIMUMAB | Antibody | TNF-alpha inhibitor | Inhibitor | psoriatic arthritis | 4 | Completed |
| TNF | GOLIMUMAB | Antibody | TNF-alpha inhibitor | Inhibitor | psoriatic arthritis | 4 | Recruiting |
| TNF | ETANERCEPT | Protein | TNF-alpha inhibitor | Inhibitor | ankylosing spondylitis | 4 |  |
| TNF | ETANERCEPT | Protein | TNF-alpha inhibitor | Inhibitor | ankylosing spondylitis | 4 | Completed |
| TNF | ETANERCEPT | Protein | TNF-alpha inhibitor | Inhibitor | ankylosing spondylitis | 4 | Not yet recruiting |
| TNF | ETANERCEPT | Protein | TNF-alpha inhibitor | Inhibitor | ankylosing spondylitis | 4 | Terminated |
| TNF | ETANERCEPT | Protein | TNF-alpha inhibitor | Inhibitor | ankylosing spondylitis | 4 | Unknown status |
| TNF | ADALIMUMAB | Antibody | TNF-alpha inhibitor | Inhibitor | ankylosing spondylitis | 4 | Completed |
| TNF | ADALIMUMAB | Antibody | TNF-alpha inhibitor | Inhibitor | ankylosing spondylitis | 4 | Not yet recruiting |
| TNF | INFLIXIMAB | Antibody | TNF-alpha inhibitor | Inhibitor | ankylosing spondylitis | 4 |  |
| TNF | INFLIXIMAB | Antibody | TNF-alpha inhibitor | Inhibitor | ankylosing spondylitis | 4 | Completed |
| TNF | INFLIXIMAB | Antibody | TNF-alpha inhibitor | Inhibitor | ankylosing spondylitis | 4 | Not yet recruiting |
| TNF | INFLIXIMAB | Antibody | TNF-alpha inhibitor | Inhibitor | ankylosing spondylitis | 4 | Unknown status |
| TNF | GOLIMUMAB | Antibody | TNF-alpha inhibitor | Inhibitor | ankylosing spondylitis | 4 |  |
| TNF | ADALIMUMAB | Antibody | TNF-alpha inhibitor | Inhibitor | ankylosing spondylitis | 4 |  |
| TNF | ADALIMUMAB | Antibody | TNF-alpha inhibitor | Inhibitor | ankylosing spondylitis | 4 | Active, not recruiting |
| TNF | ADALIMUMAB | Antibody | TNF-alpha inhibitor | Inhibitor | ankylosing spondylitis | 4 | Terminated |
| TNF | ADALIMUMAB | Antibody | TNF-alpha inhibitor | Inhibitor | ankylosing spondylitis | 4 | Unknown status |
| TNF | INFLIXIMAB | Antibody | TNF-alpha inhibitor | Inhibitor | ankylosing spondylitis | 4 | Withdrawn |
| TNF | CERTOLIZUMAB PEGOL | Antibody | TNF-alpha inhibitor | Inhibitor | ankylosing spondylitis | 4 |  |
| TNF | GOLIMUMAB | Antibody | TNF-alpha inhibitor | Inhibitor | ankylosing spondylitis | 4 | Completed |
| TNF | GOLIMUMAB | Antibody | TNF-alpha inhibitor | Inhibitor | ankylosing spondylitis | 4 | Not yet recruiting |
| TNF | ADALIMUMAB | Antibody | TNF-alpha inhibitor | Inhibitor | arthritis | 4 |  |
| TNF | ETANERCEPT | Protein | TNF-alpha inhibitor | Inhibitor | psoriasis vulgaris | 4 |  |
| TNF | ETANERCEPT | Protein | TNF-alpha inhibitor | Inhibitor | psoriasis vulgaris | 4 | Completed |
| TNF | GOLIMUMAB | Antibody | TNF-alpha inhibitor | Inhibitor | spondylitis | 4 |  |
| TNF | ETANERCEPT | Protein | TNF-alpha inhibitor | Inhibitor | spondylitis | 4 |  |
| TNF | CERTOLIZUMAB PEGOL | Antibody | TNF-alpha inhibitor | Inhibitor | inflammation | 4 |  |
| TNF | ADALIMUMAB | Antibody | TNF-alpha inhibitor | Inhibitor | Crohn's disease | 3 | Terminated |
| TNF | ADALIMUMAB | Antibody | TNF-alpha inhibitor | Inhibitor | Crohn's disease | 3 | Withdrawn |
| TNF | INFLIXIMAB | Antibody | TNF-alpha inhibitor | Inhibitor | Crohn's disease | 3 | Completed |
| TNF | INFLIXIMAB | Antibody | TNF-alpha inhibitor | Inhibitor | chronic obstructive pulmonary disease | 3 | Completed |
| TNF | CERTOLIZUMAB PEGOL | Antibody | TNF-alpha inhibitor | Inhibitor | Crohn's disease | 3 | Terminated |
| TNF | ADALIMUMAB | Antibody | TNF-alpha inhibitor | Inhibitor | Crohn's disease | 3 | Completed |
| TNF | ADALIMUMAB | Antibody | TNF-alpha inhibitor | Inhibitor | Crohn's disease | 3 | Unknown status |
| TNF | INFLIXIMAB | Antibody | TNF-alpha inhibitor | Inhibitor | Crohn's disease | 3 | Terminated |
| TNF | INFLIXIMAB | Antibody | TNF-alpha inhibitor | Inhibitor | Crohn's disease | 3 | Unknown status |
| TNF | CERTOLIZUMAB PEGOL | Antibody | TNF-alpha inhibitor | Inhibitor | Crohn's disease | 3 | Completed |
| TNF | CERTOLIZUMAB PEGOL | Antibody | TNF-alpha inhibitor | Inhibitor | Crohn's disease | 3 | Unknown status |
| TNF | CERTOLIZUMAB PEGOL | Antibody | TNF-alpha inhibitor | Inhibitor | Crohn's disease | 3 | Withdrawn |
| TNF | ETANERCEPT | Protein | TNF-alpha inhibitor | Inhibitor | psoriasis | 3 | Completed |
| TNF | ETANERCEPT | Protein | TNF-alpha inhibitor | Inhibitor | psoriasis | 3 | Unknown status |
| TNF | ETANERCEPT | Protein | TNF-alpha inhibitor | Inhibitor | psoriasis | 3 | Withdrawn |
| TNF | ADALIMUMAB | Antibody | TNF-alpha inhibitor | Inhibitor | psoriasis | 3 | Active, not recruiting |
| TNF | ADALIMUMAB | Antibody | TNF-alpha inhibitor | Inhibitor | psoriasis | 3 | Completed |
| TNF | INFLIXIMAB | Antibody | TNF-alpha inhibitor | Inhibitor | psoriasis | 3 | Terminated |
| TNF | CERTOLIZUMAB PEGOL | Antibody | TNF-alpha inhibitor | Inhibitor | psoriasis | 3 | Completed |
| TNF | CERTOLIZUMAB PEGOL | Antibody | TNF-alpha inhibitor | Inhibitor | psoriasis | 3 | Recruiting |
| TNF | ETANERCEPT | Protein | TNF-alpha inhibitor | Inhibitor | rheumatoid arthritis | 3 | Completed |
| TNF | ETANERCEPT | Protein | TNF-alpha inhibitor | Inhibitor | rheumatoid arthritis | 3 | Unknown status |
| TNF | ADALIMUMAB | Antibody | TNF-alpha inhibitor | Inhibitor | rheumatoid arthritis | 3 | Completed |
| TNF | ADALIMUMAB | Antibody | TNF-alpha inhibitor | Inhibitor | rheumatoid arthritis | 3 | Recruiting |
| TNF | ADALIMUMAB | Antibody | TNF-alpha inhibitor | Inhibitor | rheumatoid arthritis | 3 | Terminated |
| TNF | INFLIXIMAB | Antibody | TNF-alpha inhibitor | Inhibitor | rheumatoid arthritis | 3 | Terminated |
| TNF | INFLIXIMAB | Antibody | TNF-alpha inhibitor | Inhibitor | rheumatoid arthritis | 3 | Unknown status |
| TNF | CERTOLIZUMAB PEGOL | Antibody | TNF-alpha inhibitor | Inhibitor | rheumatoid arthritis | 3 | Completed |
| TNF | GOLIMUMAB | Antibody | TNF-alpha inhibitor | Inhibitor | rheumatoid arthritis | 3 | Terminated |
| TNF | ADALIMUMAB | Antibody | TNF-alpha inhibitor | Inhibitor | rheumatoid arthritis | 3 | Active, not recruiting |
| TNF | INFLIXIMAB | Antibody | TNF-alpha inhibitor | Inhibitor | rheumatoid arthritis | 3 | Completed |
| TNF | INFLIXIMAB | Antibody | TNF-alpha inhibitor | Inhibitor | rheumatoid arthritis | 3 | Recruiting |
| TNF | CERTOLIZUMAB PEGOL | Antibody | TNF-alpha inhibitor | Inhibitor | rheumatoid arthritis | 3 | Terminated |
| TNF | GOLIMUMAB | Antibody | TNF-alpha inhibitor | Inhibitor | rheumatoid arthritis | 3 | Completed |
| TNF | GOLIMUMAB | Antibody | TNF-alpha inhibitor | Inhibitor | rheumatoid arthritis | 3 | Recruiting |
| TNF | ETANERCEPT | Protein | TNF-alpha inhibitor | Inhibitor | psoriasis | 3 | Active, not recruiting |
| TNF | ADALIMUMAB | Antibody | TNF-alpha inhibitor | Inhibitor | psoriasis | 3 | Recruiting |
| TNF | INFLIXIMAB | Antibody | TNF-alpha inhibitor | Inhibitor | psoriasis | 3 | Completed |
| TNF | ADALIMUMAB | Antibody | TNF-alpha inhibitor | Inhibitor | spondyloarthropathy | 3 | Active, not recruiting |
| TNF | GOLIMUMAB | Antibody | TNF-alpha inhibitor | Inhibitor | spondyloarthropathy | 3 | Completed |
| TNF | ADALIMUMAB | Antibody | TNF-alpha inhibitor | Inhibitor | ulcerative colitis | 3 | Active, not recruiting |
| TNF | INFLIXIMAB | Antibody | TNF-alpha inhibitor | Inhibitor | ulcerative colitis | 3 | Active, not recruiting |
| TNF | INFLIXIMAB | Antibody | TNF-alpha inhibitor | Inhibitor | ulcerative colitis | 3 | Terminated |
| TNF | GOLIMUMAB | Antibody | TNF-alpha inhibitor | Inhibitor | ulcerative colitis | 3 | Completed |
| TNF | ADALIMUMAB | Antibody | TNF-alpha inhibitor | Inhibitor | ulcerative colitis | 3 | Completed |
| TNF | ADALIMUMAB | Antibody | TNF-alpha inhibitor | Inhibitor | ulcerative colitis | 3 | Unknown status |
| TNF | INFLIXIMAB | Antibody | TNF-alpha inhibitor | Inhibitor | ulcerative colitis | 3 | Completed |
| TNF | GOLIMUMAB | Antibody | TNF-alpha inhibitor | Inhibitor | ulcerative colitis | 3 | Active, not recruiting |
| TNF | ETANERCEPT | Protein | TNF-alpha inhibitor | Inhibitor | rheumatoid arthritis | 3 | Recruiting |
| TNF | ETANERCEPT | Protein | TNF-alpha inhibitor | Inhibitor | rheumatoid arthritis | 3 | Terminated |
| TNF | ETANERCEPT | Protein | TNF-alpha inhibitor | Inhibitor | rheumatoid arthritis | 3 | Withdrawn |
| TNF | ETANERCEPT | Protein | TNF-alpha inhibitor | Inhibitor | juvenile idiopathic arthritis | 3 | Completed |
| TNF | ETANERCEPT | Protein | TNF-alpha inhibitor | Inhibitor | juvenile idiopathic arthritis | 3 | Terminated |
| TNF | INFLIXIMAB | Antibody | TNF-alpha inhibitor | Inhibitor | juvenile idiopathic arthritis | 3 | Completed |
| TNF | GOLIMUMAB | Antibody | TNF-alpha inhibitor | Inhibitor | juvenile idiopathic arthritis | 3 | Active, not recruiting |
| TNF | GOLIMUMAB | Antibody | TNF-alpha inhibitor | Inhibitor | juvenile idiopathic arthritis | 3 | Terminated |
| TNF | ADALIMUMAB | Antibody | TNF-alpha inhibitor | Inhibitor | juvenile idiopathic arthritis | 3 | Completed |
| TNF | CERTOLIZUMAB PEGOL | Antibody | TNF-alpha inhibitor | Inhibitor | juvenile idiopathic arthritis | 3 | Completed |
| TNF | ETANERCEPT | Protein | TNF-alpha inhibitor | Inhibitor | pneumonia | 3 | Completed |
| TNF | ADALIMUMAB | Antibody | TNF-alpha inhibitor | Inhibitor | inflammatory bowel disease | 3 | Recruiting |
| TNF | ETANERCEPT | Protein | TNF-alpha inhibitor | Inhibitor | psoriatic arthritis | 3 | Recruiting |
| TNF | ADALIMUMAB | Antibody | TNF-alpha inhibitor | Inhibitor | psoriatic arthritis | 3 | Terminated |
| TNF | INFLIXIMAB | Antibody | TNF-alpha inhibitor | Inhibitor | psoriatic arthritis | 3 | Completed |
| TNF | INFLIXIMAB | Antibody | TNF-alpha inhibitor | Inhibitor | psoriatic arthritis | 3 | Recruiting |
| TNF | CERTOLIZUMAB PEGOL | Antibody | TNF-alpha inhibitor | Inhibitor | psoriatic arthritis | 3 | Recruiting |
| TNF | GOLIMUMAB | Antibody | TNF-alpha inhibitor | Inhibitor | psoriatic arthritis | 3 | Completed |
| TNF | ONERCEPT | Protein | TNF-alpha inhibitor | Inhibitor | psoriatic arthritis | 3 | Terminated |
| TNF | ADALIMUMAB | Antibody | TNF-alpha inhibitor | Inhibitor | Behcet's syndrome | 3 | Completed |
| TNF | INFLIXIMAB | Antibody | TNF-alpha inhibitor | Inhibitor | inflammatory bowel disease | 3 | Not yet recruiting |
| TNF | INFLIXIMAB | Antibody | TNF-alpha inhibitor | Inhibitor | inflammatory bowel disease | 3 | Withdrawn |
| TNF | ETANERCEPT | Protein | TNF-alpha inhibitor | Inhibitor | psoriatic arthritis | 3 | Completed |
| TNF | ADALIMUMAB | Antibody | TNF-alpha inhibitor | Inhibitor | psoriatic arthritis | 3 | Active, not recruiting |
| TNF | ADALIMUMAB | Antibody | TNF-alpha inhibitor | Inhibitor | psoriatic arthritis | 3 | Completed |
| TNF | ADALIMUMAB | Antibody | TNF-alpha inhibitor | Inhibitor | psoriatic arthritis | 3 | Recruiting |
| TNF | GOLIMUMAB | Antibody | TNF-alpha inhibitor | Inhibitor | psoriatic arthritis | 3 | Not yet recruiting |
| TNF | GOLIMUMAB | Antibody | TNF-alpha inhibitor | Inhibitor | psoriatic arthritis | 3 | Recruiting |
| TNF | INFLIXIMAB | Antibody | TNF-alpha inhibitor | Inhibitor | Behcet's syndrome | 3 | Completed |
| TNF | INFLIXIMAB | Antibody | TNF-alpha inhibitor | Inhibitor | colitis | 3 | Recruiting |
| TNF | ETANERCEPT | Protein | TNF-alpha inhibitor | Inhibitor | ankylosing spondylitis | 3 | Completed |
| TNF | ADALIMUMAB | Antibody | TNF-alpha inhibitor | Inhibitor | ankylosing spondylitis | 3 | Completed |
| TNF | ADALIMUMAB | Antibody | TNF-alpha inhibitor | Inhibitor | ankylosing spondylitis | 3 | Withdrawn |
| TNF | INFLIXIMAB | Antibody | TNF-alpha inhibitor | Inhibitor | ankylosing spondylitis | 3 | Completed |
| TNF | GOLIMUMAB | Antibody | TNF-alpha inhibitor | Inhibitor | ankylosing spondylitis | 3 | Completed |
| TNF | GOLIMUMAB | Antibody | TNF-alpha inhibitor | Inhibitor | ankylosing spondylitis | 3 | Terminated |
| TNF | CERTOLIZUMAB PEGOL | Antibody | TNF-alpha inhibitor | Inhibitor | ankylosing spondylitis | 3 | Completed |
| TNF | INFLIXIMAB | Antibody | TNF-alpha inhibitor | Inhibitor | mucocutaneous lymph node syndrome | 3 | Completed |
| TNF | INFLIXIMAB | Antibody | TNF-alpha inhibitor | Inhibitor | mucocutaneous lymph node syndrome | 3 | Withdrawn |
| TNF | INFLIXIMAB | Antibody | TNF-alpha inhibitor | Inhibitor | chronic hepatitis C virus infection | 3 | Completed |
| TNF | ETANERCEPT | Protein | TNF-alpha inhibitor | Inhibitor | Stevens-Johnson syndrome | 3 | Recruiting |
| TNF | INFLIXIMAB | Antibody | TNF-alpha inhibitor | Inhibitor | polymyalgia rheumatica | 3 | Unknown status |
| TNF | ADALIMUMAB | Antibody | TNF-alpha inhibitor | Inhibitor | interstitial cystitis | 3 | Completed |
| TNF | ETANERCEPT | Protein | TNF-alpha inhibitor | Inhibitor | polymyalgia rheumatica | 3 | Completed |
| TNF | INFLIXIMAB | Antibody | TNF-alpha inhibitor | Inhibitor | diabetic macular edema | 3 | Terminated |
| TNF | ADALIMUMAB | Antibody | TNF-alpha inhibitor | Inhibitor | psoriasis-related juvenile idiopathic arthritis | 3 | Recruiting |
| TNF | ADALIMUMAB | Antibody | TNF-alpha inhibitor | Inhibitor | hidradenitis suppurativa | 3 | Completed |
| TNF | CERTOLIZUMAB PEGOL | Antibody | TNF-alpha inhibitor | Inhibitor | chronic interstitial cystitis | 3 | Completed |
| TNF | INFLIXIMAB | Antibody | TNF-alpha inhibitor | Inhibitor | parapsoriasis | 3 | Completed |
| TNF | ADALIMUMAB | Antibody | TNF-alpha inhibitor | Inhibitor | osteoarthritis, hand | 3 | Completed |
| TNF | ADALIMUMAB | Antibody | TNF-alpha inhibitor | Inhibitor | uveitis | 3 | Completed |
| TNF | ADALIMUMAB | Antibody | TNF-alpha inhibitor | Inhibitor | uveitis | 3 | Not yet recruiting |
| TNF | ADALIMUMAB | Antibody | TNF-alpha inhibitor | Inhibitor | uveitis | 3 | Active, not recruiting |
| TNF | ADALIMUMAB | Antibody | TNF-alpha inhibitor | Inhibitor | uveitis | 3 | Recruiting |
| TNF | ADALIMUMAB | Antibody | TNF-alpha inhibitor | Inhibitor | temporal arteritis | 3 | Completed |
| TNF | ADALIMUMAB | Antibody | TNF-alpha inhibitor | Inhibitor | psoriasis vulgaris | 3 | Completed |
| TNF | CERTOLIZUMAB PEGOL | Antibody | TNF-alpha inhibitor | Inhibitor | psoriasis vulgaris | 3 | Recruiting |
| TNF | ADALIMUMAB | Antibody | TNF-alpha inhibitor | Inhibitor | Pyoderma | 3 | Completed |
| TNF | ETANERCEPT | Protein | TNF-alpha inhibitor | Inhibitor | Anorexia | 3 | Completed |
| TNF | INFLIXIMAB | Antibody | TNF-alpha inhibitor | Inhibitor | Anorexia | 3 | Completed |
| TNF | INFLIXIMAB | Antibody | TNF-alpha inhibitor | Inhibitor | Cachexia | 3 | Completed |
| TNF | INFLIXIMAB | Antibody | TNF-alpha inhibitor | Inhibitor | Fatigue | 3 | Completed |
| TNF | INFLIXIMAB | Antibody | TNF-alpha inhibitor | Inhibitor | Low back pain | 3 | Completed |
| TNF | ETANERCEPT | Protein | TNF-alpha inhibitor | Inhibitor | Cachexia | 3 | Completed |
| TNF | INFLIXIMAB | Antibody | TNF-alpha inhibitor | Inhibitor | scleritis | 3 | Withdrawn |
| TNF | ETANERCEPT | Protein | TNF-alpha inhibitor | Inhibitor | type 1 diabetes mellitus | 3 | Completed |
| TNF | GOLIMUMAB | Antibody | TNF-alpha inhibitor | Inhibitor | osteoarthritis | 3 | Completed |
| TNF | ADALIMUMAB | Antibody | TNF-alpha inhibitor | Inhibitor | osteoarthritis | 3 | Completed |
| TNF | INFLIXIMAB | Antibody | TNF-alpha inhibitor | Inhibitor | lung cancer | 3 | Completed |
| TNF | INFLIXIMAB | Antibody | TNF-alpha inhibitor | Inhibitor | graft versus host disease | 3 | Completed |
| TNF | ADALIMUMAB | Antibody | TNF-alpha inhibitor | Inhibitor | psoriasis 14, pustular | 3 | Completed |
| TNF | INFLIXIMAB | Antibody | TNF-alpha inhibitor | Inhibitor | sarcoidosis | 3 | Completed |
| TNF | ETANERCEPT | Protein | TNF-alpha inhibitor | Inhibitor | acute graft versus host disease | 3 | Completed |
| TNF | ETANERCEPT | Protein | TNF-alpha inhibitor | Inhibitor | polyarticular arthritis | 3 | Recruiting |
| TNF | ETANERCEPT | Protein | TNF-alpha inhibitor | Inhibitor | inflammation | 3 | Completed |
| TNF | ADALIMUMAB | Antibody | TNF-alpha inhibitor | Inhibitor | COVID-19 | 3 | Withdrawn |
| TNF | INFLIXIMAB | Antibody | TNF-alpha inhibitor | Inhibitor | COVID-19 | 3 | Active, not recruiting |
| TNF | INFLIXIMAB | Antibody | TNF-alpha inhibitor | Inhibitor | COVID-19 | 3 | Completed |
| TNF | ETANERCEPT | Protein | TNF-alpha inhibitor | Inhibitor | metabolic syndrome | 2 | Completed |
| TNF | INFLIXIMAB | Antibody | TNF-alpha inhibitor | Inhibitor | myelodysplastic syndrome | 2 | Completed |
| TNF | ADALIMUMAB | Antibody | TNF-alpha inhibitor | Inhibitor | benign prostatic hyperplasia | 2 | Recruiting |
| TNF | ADALIMUMAB | Antibody | TNF-alpha inhibitor | Inhibitor | Crohn's disease | 2 | Terminated |
| TNF | INFLIXIMAB | Antibody | TNF-alpha inhibitor | Inhibitor | Crohn's disease | 2 | Completed |
| TNF | INFLIXIMAB | Antibody | TNF-alpha inhibitor | Inhibitor | Crohn's disease | 2 | Recruiting |
| TNF | ETANERCEPT | Protein | TNF-alpha inhibitor | Inhibitor | chronic obstructive pulmonary disease | 2 | Completed |
| TNF | CERTOLIZUMAB PEGOL | Antibody | TNF-alpha inhibitor | Inhibitor | Crohn's disease | 2 | Completed |
| TNF | ETANERCEPT | Protein | TNF-alpha inhibitor | Inhibitor | dermatomyositis | 2 | Terminated |
| TNF | INFLIXIMAB | Antibody | TNF-alpha inhibitor | Inhibitor | dermatomyositis | 2 | Completed |
| TNF | ADALIMUMAB | Antibody | TNF-alpha inhibitor | Inhibitor | Crohn's disease | 2 | Completed |
| TNF | ADALIMUMAB | Antibody | TNF-alpha inhibitor | Inhibitor | Crohn's disease | 2 | Withdrawn |
| TNF | CERTOLIZUMAB PEGOL | Antibody | TNF-alpha inhibitor | Inhibitor | Crohn's disease | 2 | Terminated |
| TNF | GOLIMUMAB | Antibody | TNF-alpha inhibitor | Inhibitor | Crohn's disease | 2 | Recruiting |
| TNF | ETANERCEPT | Protein | TNF-alpha inhibitor | Inhibitor | leukemia | 2 | Terminated |
| TNF | ADALIMUMAB | Antibody | TNF-alpha inhibitor | Inhibitor | immune system disease | 2 | Recruiting |
| TNF | AFELIMOMAB | Antibody | TNF-alpha inhibitor | Inhibitor | immune system disease | 2 |  |
| TNF | ETANERCEPT | Protein | TNF-alpha inhibitor | Inhibitor | psoriasis | 2 | Completed |
| TNF | CERTOLIZUMAB PEGOL | Antibody | TNF-alpha inhibitor | Inhibitor | psoriasis | 2 | Completed |
| TNF | ETANERCEPT | Protein | TNF-alpha inhibitor | Inhibitor | rheumatoid arthritis | 2 | Active, not recruiting |
| TNF | ETANERCEPT | Protein | TNF-alpha inhibitor | Inhibitor | rheumatoid arthritis | 2 | Terminated |
| TNF | ADALIMUMAB | Antibody | TNF-alpha inhibitor | Inhibitor | rheumatoid arthritis | 2 | Active, not recruiting |
| TNF | ADALIMUMAB | Antibody | TNF-alpha inhibitor | Inhibitor | rheumatoid arthritis | 2 | Completed |
| TNF | ADALIMUMAB | Antibody | TNF-alpha inhibitor | Inhibitor | rheumatoid arthritis | 2 | Recruiting |
| TNF | INFLIXIMAB | Antibody | TNF-alpha inhibitor | Inhibitor | rheumatoid arthritis | 2 | Completed |
| TNF | GOLIMUMAB | Antibody | TNF-alpha inhibitor | Inhibitor | prostate adenocarcinoma | 2 | Recruiting |
| TNF | ADALIMUMAB | Antibody | TNF-alpha inhibitor | Inhibitor | rheumatoid arthritis | 2 | Terminated |
| TNF | ADALIMUMAB | Antibody | TNF-alpha inhibitor | Inhibitor | rheumatoid arthritis | 2 | Withdrawn |
| TNF | INFLIXIMAB | Antibody | TNF-alpha inhibitor | Inhibitor | rheumatoid arthritis | 2 | Terminated |
| TNF | CERTOLIZUMAB PEGOL | Antibody | TNF-alpha inhibitor | Inhibitor | rheumatoid arthritis | 2 | Completed |
| TNF | GOLIMUMAB | Antibody | TNF-alpha inhibitor | Inhibitor | rheumatoid arthritis | 2 | Completed |
| TNF | GOLIMUMAB | Antibody | TNF-alpha inhibitor | Inhibitor | rheumatoid arthritis | 2 | Terminated |
| TNF | ETANERCEPT | Protein | TNF-alpha inhibitor | Inhibitor | psoriasis | 2 | Active, not recruiting |
| TNF | ADALIMUMAB | Antibody | TNF-alpha inhibitor | Inhibitor | psoriasis | 2 | Completed |
| TNF | INFLIXIMAB | Antibody | TNF-alpha inhibitor | Inhibitor | psoriasis | 2 | Completed |
| TNF | OZORALIZUMAB | Antibody | TNF-alpha inhibitor | Inhibitor | rheumatoid arthritis | 2 | Completed |
| TNF | PEGSUNERCEPT | Protein | TNF-alpha inhibitor | Inhibitor | rheumatoid arthritis | 2 | Completed |
| TNF | PEGSUNERCEPT | Protein | TNF-alpha inhibitor | Inhibitor | rheumatoid arthritis | 2 | Terminated |
| TNF | PLACULUMAB | Antibody | TNF-alpha inhibitor | Inhibitor | rheumatoid arthritis | 2 | Completed |
| TNF | PLACULUMAB | Antibody | TNF-alpha inhibitor | Inhibitor | rheumatoid arthritis | 2 | Terminated |
| TNF | ABBV-3373 | Unknown | TNF-alpha binding agent | Inhibitor | rheumatoid arthritis | 2 | Completed |
| TNF | REMTOLUMAB | Antibody | TNF-alpha inhibitor | Inhibitor | rheumatoid arthritis | 2 | Completed |
| TNF | INFLIXIMAB | Antibody | TNF-alpha inhibitor | Inhibitor | spondyloarthropathy | 2 | Terminated |
| TNF | INFLIXIMAB | Antibody | TNF-alpha inhibitor | Inhibitor | spondyloarthropathy | 2 | Completed |
| TNF | ETANERCEPT | Protein | TNF-alpha inhibitor | Inhibitor | Sjogren syndrome | 2 | Completed |
| TNF | ADALIMUMAB | Antibody | TNF-alpha inhibitor | Inhibitor | ulcerative colitis | 2 | Recruiting |
| TNF | ADALIMUMAB | Antibody | TNF-alpha inhibitor | Inhibitor | ulcerative colitis | 2 | Terminated |
| TNF | CERTOLIZUMAB PEGOL | Antibody | TNF-alpha inhibitor | Inhibitor | ulcerative colitis | 2 | Completed |
| TNF | GOLIMUMAB | Antibody | TNF-alpha inhibitor | Inhibitor | ulcerative colitis | 2 | Recruiting |
| TNF | GOLIMUMAB | Antibody | TNF-alpha inhibitor | Inhibitor | ulcerative colitis | 2 | Terminated |
| TNF | ADALIMUMAB | Antibody | TNF-alpha inhibitor | Inhibitor | ulcerative colitis | 2 | Completed |
| TNF | INFLIXIMAB | Antibody | TNF-alpha inhibitor | Inhibitor | ulcerative colitis | 2 | Not yet recruiting |
| TNF | GOLIMUMAB | Antibody | TNF-alpha inhibitor | Inhibitor | ulcerative colitis | 2 | Completed |
| TNF | ETANERCEPT | Protein | TNF-alpha inhibitor | Inhibitor | rheumatoid arthritis | 2 | Completed |
| TNF | INFLIXIMAB | Antibody | TNF-alpha inhibitor | Inhibitor | endometriosis | 2 | Completed |
| TNF | ADALIMUMAB | Antibody | TNF-alpha inhibitor | Inhibitor | age-related macular degeneration | 2 | Completed |
| TNF | INFLIXIMAB | Antibody | TNF-alpha inhibitor | Inhibitor | age-related macular degeneration | 2 | Completed |
| TNF | INFLIXIMAB | Antibody | TNF-alpha inhibitor | Inhibitor | myositis | 2 | Completed |
| TNF | ETANERCEPT | Protein | TNF-alpha inhibitor | Inhibitor | juvenile idiopathic arthritis | 2 | Completed |
| TNF | INFLIXIMAB | Antibody | TNF-alpha inhibitor | Inhibitor | juvenile idiopathic arthritis | 2 | Terminated |
| TNF | GOLIMUMAB | Antibody | TNF-alpha inhibitor | Inhibitor | prostate carcinoma | 2 | Recruiting |
| TNF | INFLIXIMAB | Antibody | TNF-alpha inhibitor | Inhibitor | metastatic melanoma | 2 | Recruiting |
| TNF | ETANERCEPT | Protein | TNF-alpha inhibitor | Inhibitor | ischemia reperfusion injury | 2 | Completed |
| TNF | INFLIXIMAB | Antibody | TNF-alpha inhibitor | Inhibitor | ischemia reperfusion injury | 2 | Completed |
| TNF | INFLIXIMAB | Antibody | TNF-alpha inhibitor | Inhibitor | hepatitis C virus infection | 2 | Completed |
| TNF | ADALIMUMAB | Antibody | TNF-alpha inhibitor | Inhibitor | psoriatic arthritis | 2 | Completed |
| TNF | GOLIMUMAB | Antibody | TNF-alpha inhibitor | Inhibitor | psoriatic arthritis | 2 | Active, not recruiting |
| TNF | INFLIXIMAB | Antibody | TNF-alpha inhibitor | Inhibitor | polymyositis | 2 | Completed |
| TNF | REMTOLUMAB | Antibody | TNF-alpha inhibitor | Inhibitor | psoriatic arthritis | 2 | Completed |
| TNF | REMTOLUMAB | Antibody | TNF-alpha inhibitor | Inhibitor | psoriatic arthritis | 2 | Terminated |
| TNF | ETANERCEPT | Protein | TNF-alpha inhibitor | Inhibitor | cutaneous lupus erythematosus | 2 | Completed |
| TNF | ADALIMUMAB | Antibody | TNF-alpha inhibitor | Inhibitor | colitis | 2 | Withdrawn |
| TNF | INFLIXIMAB | Antibody | TNF-alpha inhibitor | Inhibitor | colitis | 2 | Withdrawn |
| TNF | ETANERCEPT | Protein | TNF-alpha inhibitor | Inhibitor | chronic kidney disease | 2 | Terminated |
| TNF | ADALIMUMAB | Antibody | TNF-alpha inhibitor | Inhibitor | Dupuytren Contracture | 2 | Completed |
| TNF | ETANERCEPT | Protein | TNF-alpha inhibitor | Inhibitor | Vitiligo | 2 | Completed |
| TNF | ADALIMUMAB | Antibody | TNF-alpha inhibitor | Inhibitor | ankylosing spondylitis | 2 | Not yet recruiting |
| TNF | ADALIMUMAB | Antibody | TNF-alpha inhibitor | Inhibitor | ankylosing spondylitis | 2 | Unknown status |
| TNF | ETANERCEPT | Protein | TNF-alpha inhibitor | Inhibitor | ankylosing spondylitis | 2 | Unknown status |
| TNF | CERTOLIZUMAB PEGOL | Antibody | TNF-alpha inhibitor | Inhibitor | ankylosing spondylitis | 2 | Completed |
| TNF | ETANERCEPT | Protein | TNF-alpha inhibitor | Inhibitor | mucocutaneous lymph node syndrome | 2 | Completed |
| TNF | INFLIXIMAB | Antibody | TNF-alpha inhibitor | Inhibitor | eosinophilic esophagitis | 2 | Completed |
| TNF | ADALIMUMAB | Antibody | TNF-alpha inhibitor | Inhibitor | focal segmental glomerulosclerosis | 2 | Completed |
| TNF | ADALIMUMAB | Antibody | TNF-alpha inhibitor | Inhibitor | osteoarthritis, knee | 2 | Completed |
| TNF | INFLIXIMAB | Antibody | TNF-alpha inhibitor | Inhibitor | acute graft vs. host disease | 2 | Terminated |
| TNF | INFLIXIMAB | Antibody | TNF-alpha inhibitor | Inhibitor | toxic epidermal necrolysis | 2 | Withdrawn |
| TNF | ETANERCEPT | Protein | TNF-alpha inhibitor | Inhibitor | pemphigus vulgaris | 2 | Completed |
| TNF | ETANERCEPT | Protein | TNF-alpha inhibitor | Inhibitor | Granulomatosis with Polyangiitis | 2 | Completed |
| TNF | INFLIXIMAB | Antibody | TNF-alpha inhibitor | Inhibitor | Granulomatosis with Polyangiitis | 2 | Completed |
| TNF | ETANERCEPT | Protein | TNF-alpha inhibitor | Inhibitor | lupus nephritis | 2 | Terminated |
| TNF | ETANERCEPT | Protein | TNF-alpha inhibitor | Inhibitor | temporomandibular joint disorder | 2 | Completed |
| TNF | ETANERCEPT | Protein | TNF-alpha inhibitor | Inhibitor | synovitis | 2 | Completed |
| TNF | INFLIXIMAB | Antibody | TNF-alpha inhibitor | Inhibitor | reactive arthritis | 2 | Unknown status |
| TNF | ETANERCEPT | Protein | TNF-alpha inhibitor | Inhibitor | pulmonary fibrosis | 2 | Completed |
| TNF | INFLIXIMAB | Antibody | TNF-alpha inhibitor | Inhibitor | tropical spastic paraparesis | 2 | Terminated |
| TNF | ETANERCEPT | Protein | TNF-alpha inhibitor | Inhibitor | stomatitis | 2 | Terminated |
| TNF | ADALIMUMAB | Antibody | TNF-alpha inhibitor | Inhibitor | hidradenitis suppurativa | 2 | Terminated |
| TNF | INFLIXIMAB | Antibody | TNF-alpha inhibitor | Inhibitor | hidradenitis suppurativa | 2 | Completed |
| TNF | ETANERCEPT | Protein | TNF-alpha inhibitor | Inhibitor | lichen planus | 2 | Terminated |
| TNF | ETANERCEPT | Protein | TNF-alpha inhibitor | Inhibitor | SAPHO syndrome | 2 | Recruiting |
| TNF | INFLIXIMAB | Antibody | TNF-alpha inhibitor | Inhibitor | microscopic polyangiitis | 2 | Completed |
| TNF | ADALIMUMAB | Antibody | TNF-alpha inhibitor | Inhibitor | uveitis | 2 | Completed |
| TNF | ADALIMUMAB | Antibody | TNF-alpha inhibitor | Inhibitor | uveitis | 2 | Unknown status |
| TNF | ADALIMUMAB | Antibody | TNF-alpha inhibitor | Inhibitor | uveitis | 2 | Not yet recruiting |
| TNF | ADALIMUMAB | Antibody | TNF-alpha inhibitor | Inhibitor | uveitis | 2 | Recruiting |
| TNF | ETANERCEPT | Protein | TNF-alpha inhibitor | Inhibitor | hidradenitis suppurativa | 2 | Completed |
| TNF | ADALIMUMAB | Antibody | TNF-alpha inhibitor | Inhibitor | hidradenitis suppurativa | 2 | Completed |
| TNF | ETANERCEPT | Protein | TNF-alpha inhibitor | Inhibitor | lichen planus | 2 | Active, not recruiting |
| TNF | INFLIXIMAB | Antibody | TNF-alpha inhibitor | Inhibitor | pemphigus | 2 | Completed |
| TNF | ADALIMUMAB | Antibody | TNF-alpha inhibitor | Inhibitor | frozen shoulder | 2 | Completed |
| TNF | INFLIXIMAB | Antibody | TNF-alpha inhibitor | Inhibitor | temporal arteritis | 2 | Terminated |
| TNF | INFLIXIMAB | Antibody | TNF-alpha inhibitor | Inhibitor | Takayasu arteritis | 2 | Unknown status |
| TNF | INFLIXIMAB | Antibody | TNF-alpha inhibitor | Inhibitor | Takayasu arteritis | 2 | Not yet recruiting |
| TNF | ADALIMUMAB | Antibody | TNF-alpha inhibitor | Inhibitor | Pyoderma | 2 | Withdrawn |
| TNF | ETANERCEPT | Protein | TNF-alpha inhibitor | Inhibitor | Tinnitus | 2 | Recruiting |
| TNF | ADALIMUMAB | Antibody | TNF-alpha inhibitor | Inhibitor | Sciatica | 2 | Completed |
| TNF | ETANERCEPT | Protein | TNF-alpha inhibitor | Inhibitor | Histiocytosis | 2 | Completed |
| TNF | ETANERCEPT | Protein | TNF-alpha inhibitor | Inhibitor | radiculopathy | 2 | Completed |
| TNF | ETANERCEPT | Protein | TNF-alpha inhibitor | Inhibitor | lupus erythematosus | 2 | Completed |
| TNF | ETANERCEPT | Protein | TNF-alpha inhibitor | Inhibitor | Alzheimer disease | 2 | Completed |
| TNF | ETANERCEPT | Protein | TNF-alpha inhibitor | Inhibitor | asthma | 2 | Completed |
| TNF | ADALIMUMAB | Antibody | TNF-alpha inhibitor | Inhibitor | asthma | 2 | Withdrawn |
| TNF | GOLIMUMAB | Antibody | TNF-alpha inhibitor | Inhibitor | asthma | 2 | Completed |
| TNF | GOLIMUMAB | Antibody | TNF-alpha inhibitor | Inhibitor | asthma | 2 | Withdrawn |
| TNF | INFLIXIMAB | Antibody | TNF-alpha inhibitor | Inhibitor | bipolar disorder | 2 | Completed |
| TNF | ETANERCEPT | Protein | TNF-alpha inhibitor | Inhibitor | type 1 diabetes mellitus | 2 | Completed |
| TNF | INFLIXIMAB | Antibody | TNF-alpha inhibitor | Inhibitor | type 1 diabetes mellitus | 2 | Completed |
| TNF | GOLIMUMAB | Antibody | TNF-alpha inhibitor | Inhibitor | type 1 diabetes mellitus | 2 | Completed |
| TNF | GOLIMUMAB | Antibody | TNF-alpha inhibitor | Inhibitor | type 1 diabetes mellitus | 2 | Withdrawn |
| TNF | ADALIMUMAB | Antibody | TNF-alpha inhibitor | Inhibitor | osteoarthritis | 2 | Completed |
| TNF | INFLIXIMAB | Antibody | TNF-alpha inhibitor | Inhibitor | osteoarthritis | 2 | Withdrawn |
| TNF | ADALIMUMAB | Antibody | TNF-alpha inhibitor | Inhibitor | Netherton syndrome | 2 | Completed |
| TNF | CERTOLIZUMAB PEGOL | Antibody | TNF-alpha inhibitor | Inhibitor | psoriasis 14, pustular | 2 | Completed |
| TNF | ETANERCEPT | Protein | TNF-alpha inhibitor | Inhibitor | graft versus host disease | 2 | Completed |
| TNF | INFLIXIMAB | Antibody | TNF-alpha inhibitor | Inhibitor | graft versus host disease | 2 | Completed |
| TNF | INFLIXIMAB | Antibody | TNF-alpha inhibitor | Inhibitor | graft versus host disease | 2 | Terminated |
| TNF | ETANERCEPT | Protein | TNF-alpha inhibitor | Inhibitor | primary systemic amyloidosis | 2 | Completed |
| TNF | ADALIMUMAB | Antibody | TNF-alpha inhibitor | Inhibitor | sarcoidosis | 2 | Completed |
| TNF | GOLIMUMAB | Antibody | TNF-alpha inhibitor | Inhibitor | sarcoidosis | 2 | Completed |
| TNF | ETANERCEPT | Protein | TNF-alpha inhibitor | Inhibitor | discoid lupus erythematosus | 2 | Unknown status |
| TNF | ADALIMUMAB | Antibody | TNF-alpha inhibitor | Inhibitor | sarcoidosis | 2 | Terminated |
| TNF | ADALIMUMAB | Antibody | TNF-alpha inhibitor | Inhibitor | sarcoidosis | 2 | Withdrawn |
| TNF | INFLIXIMAB | Antibody | TNF-alpha inhibitor | Inhibitor | COVID-19 | 2 | Completed |
| TNF | INFLIXIMAB | Antibody | TNF-alpha inhibitor | Inhibitor | choroidal neovascularization | 2 | Completed |
| TNF | ADALIMUMAB | Antibody | TNF-alpha inhibitor | Inhibitor | inflammation | 2 | Withdrawn |
| TNF | ADALIMUMAB | Antibody | TNF-alpha inhibitor | Inhibitor | choroidal neovascularization | 2 | Completed |
| TNF | ETANERCEPT | Protein | TNF-alpha inhibitor | Inhibitor | inflammation | 2 | Completed |
| TNF | ETANERCEPT | Protein | TNF-alpha inhibitor | Inhibitor | chronic idiopathic urticaria | 2 | Withdrawn |
| TNF | ETANERCEPT | Protein | TNF-alpha inhibitor | Inhibitor | chronic lymphocytic leukemia | 1 | Completed |
| TNF | ETANERCEPT | Protein | TNF-alpha inhibitor | Inhibitor | myelodysplastic syndrome | 1 | Completed |
| TNF | ETANERCEPT | Protein | TNF-alpha inhibitor | Inhibitor | dermatomyositis | 1 | Completed |
| TNF | INFLIXIMAB | Antibody | TNF-alpha inhibitor | Inhibitor | Crohn's disease | 1 | Completed |
| TNF | ADALIMUMAB | Antibody | TNF-alpha inhibitor | Inhibitor | immune system disease | 1 | Completed |
| TNF | ETANERCEPT | Protein | TNF-alpha inhibitor | Inhibitor | neoplasm | 1 | Completed |
| TNF | ETANERCEPT | Protein | TNF-alpha inhibitor | Inhibitor | rheumatoid arthritis | 1 | Active, not recruiting |
| TNF | CERTOLIZUMAB PEGOL | Antibody | TNF-alpha inhibitor | Inhibitor | rheumatoid arthritis | 1 | Completed |
| TNF | GOLIMUMAB | Antibody | TNF-alpha inhibitor | Inhibitor | rheumatoid arthritis | 1 | Completed |
| TNF | INFLIXIMAB | Antibody | TNF-alpha inhibitor | Inhibitor | rheumatoid arthritis | 1 | Completed |
| TNF | ETANERCEPT | Protein | TNF-alpha inhibitor | Inhibitor | psoriasis | 1 | Completed |
| TNF | ETANERCEPT | Protein | TNF-alpha inhibitor | Inhibitor | psoriasis | 1 | Unknown status |
| TNF | ADALIMUMAB | Antibody | TNF-alpha inhibitor | Inhibitor | psoriasis | 1 | Completed |
| TNF | ADALIMUMAB | Antibody | TNF-alpha inhibitor | Inhibitor | psoriasis | 1 | Terminated |
| TNF | OZORALIZUMAB | Antibody | TNF-alpha inhibitor | Inhibitor | rheumatoid arthritis | 1 | Completed |
| TNF | REMTOLUMAB | Antibody | TNF-alpha inhibitor | Inhibitor | rheumatoid arthritis | 1 | Completed |
| TNF | ADALIMUMAB | Antibody | TNF-alpha inhibitor | Inhibitor | ulcerative colitis | 1 | Completed |
| TNF | INFLIXIMAB | Antibody | TNF-alpha inhibitor | Inhibitor | ulcerative colitis | 1 | Completed |
| TNF | INFLIXIMAB | Antibody | TNF-alpha inhibitor | Inhibitor | ulcerative colitis | 1 | Not yet recruiting |
| TNF | GOLIMUMAB | Antibody | TNF-alpha inhibitor | Inhibitor | ulcerative colitis | 1 | Completed |
| TNF | ETANERCEPT | Protein | TNF-alpha inhibitor | Inhibitor | rheumatoid arthritis | 1 | Completed |
| TNF | ETANERCEPT | Protein | TNF-alpha inhibitor | Inhibitor | rheumatoid arthritis | 1 | Terminated |
| TNF | ETANERCEPT | Protein | TNF-alpha inhibitor | Inhibitor | rheumatoid arthritis | 1 | Unknown status |
| TNF | ADALIMUMAB | Antibody | TNF-alpha inhibitor | Inhibitor | rheumatoid arthritis | 1 | Completed |
| TNF | INFLIXIMAB | Antibody | TNF-alpha inhibitor | Inhibitor | melanoma | 1 | Completed |
| TNF | CERTOLIZUMAB PEGOL | Antibody | TNF-alpha inhibitor | Inhibitor | melanoma | 1 | Completed |
| TNF | ETANERCEPT | Protein | TNF-alpha inhibitor | Inhibitor | subarachnoid hemorrhage | 1 | Withdrawn |
| TNF | ETANERCEPT | Protein | TNF-alpha inhibitor | Inhibitor | HIV infection | 1 | Completed |
| TNF | INFLIXIMAB | Antibody | TNF-alpha inhibitor | Inhibitor | ischemia reperfusion injury | 1 | Completed |
| TNF | GOLIMUMAB | Antibody | TNF-alpha inhibitor | Inhibitor | psoriatic arthritis | 1 | Completed |
| TNF | INFLIXIMAB | Antibody | TNF-alpha inhibitor | Inhibitor | inflammatory bowel disease | 1 | Completed |
| TNF | INFLIXIMAB | Antibody | TNF-alpha inhibitor | Inhibitor | ankylosing spondylitis | 1 | Completed |
| TNF | ADALIMUMAB | Antibody | TNF-alpha inhibitor | Inhibitor | ankylosing spondylitis | 1 | Not yet recruiting |
| TNF | INFLIXIMAB | Antibody | TNF-alpha inhibitor | Inhibitor | mucocutaneous lymph node syndrome | 1 | Completed |
| TNF | ADALIMUMAB | Antibody | TNF-alpha inhibitor | Inhibitor | focal segmental glomerulosclerosis | 1 | Completed |
| TNF | INFLIXIMAB | Antibody | TNF-alpha inhibitor | Inhibitor | Stevens-Johnson syndrome | 1 | Withdrawn |
| TNF | ETANERCEPT | Protein | TNF-alpha inhibitor | Inhibitor | persian gulf syndrome | 1 | Recruiting |
| TNF | INFLIXIMAB | Antibody | TNF-alpha inhibitor | Inhibitor | berylliosis | 1 | Terminated |
| TNF | INFLIXIMAB | Antibody | TNF-alpha inhibitor | Inhibitor | diabetic macular edema | 1 | Unknown status |
| TNF | ETANERCEPT | Protein | TNF-alpha inhibitor | Inhibitor | Sciatica | 1 | Completed |
| TNF | PLACULUMAB | Antibody | TNF-alpha inhibitor | Inhibitor | Sciatica | 1 | Completed |
| TNF | INFLIXIMAB | Antibody | TNF-alpha inhibitor | Inhibitor | scleritis | 1 | Completed |
| TNF | ADALIMUMAB | Antibody | TNF-alpha inhibitor | Inhibitor | thyroid cancer | 1 | Withdrawn |
| TNF | ETANERCEPT | Protein | TNF-alpha inhibitor | Inhibitor | Alzheimer disease | 1 | Completed |
| TNF | ETANERCEPT | Protein | TNF-alpha inhibitor | Inhibitor | type 1 diabetes mellitus | 1 | Active, not recruiting |
| TNF | ETANERCEPT | Protein | TNF-alpha inhibitor | Inhibitor | type 1 diabetes mellitus | 1 | Completed |
| TNF | GOLIMUMAB | Antibody | TNF-alpha inhibitor | Inhibitor | type 1 diabetes mellitus | 1 | Completed |
| TNF | ETANERCEPT | Protein | TNF-alpha inhibitor | Inhibitor | type 1 diabetes mellitus | 1 | Withdrawn |
| TNF | INFLIXIMAB | Antibody | TNF-alpha inhibitor | Inhibitor | glaucoma | 1 | Recruiting |
| TNF | ADALIMUMAB | Antibody | TNF-alpha inhibitor | Inhibitor | osteoarthritis | 1 | Completed |
| TNF | ETANERCEPT | Protein | TNF-alpha inhibitor | Inhibitor | prostate cancer | 1 | Completed |
| TNF | ADALIMUMAB | Antibody | TNF-alpha inhibitor | Inhibitor | mucopolysaccharidosis type 6 | 1 | Active, not recruiting |
| TNF | ADALIMUMAB | Antibody | TNF-alpha inhibitor | Inhibitor | mucopolysaccharidosis type 2 | 1 | Active, not recruiting |
| TNF | INFLIXIMAB | Antibody | TNF-alpha inhibitor | Inhibitor | graft versus host disease | 1 | Completed |
| TNF | INFLIXIMAB | Antibody | TNF-alpha inhibitor | Inhibitor | chronic granulomatous disease | 1 | Terminated |
| TNF | GOLIMUMAB | Antibody | TNF-alpha inhibitor | Inhibitor | autoimmune inner ear disease | 1 | Terminated |
| TNF | ETANERCEPT | Protein | TNF-alpha inhibitor | Inhibitor | inclusion body myositis | 0.5 | Completed |
| TNF | INFLIXIMAB | Antibody | TNF-alpha inhibitor | Inhibitor | hidradenitis suppurativa | 0.5 | Completed |
| TNF | ADALIMUMAB | Antibody | TNF-alpha inhibitor | Inhibitor | innate immune response | 0.5 | Completed |
| TNF | ETANERCEPT | Protein | TNF-alpha inhibitor | Inhibitor | type 1 diabetes mellitus | 0.5 | Completed |
| TNF | GOLIMUMAB | Antibody | TNF-alpha inhibitor | Inhibitor | type 1 diabetes mellitus | 0.5 | Completed |
| TNF | ETANERCEPT | Protein | TNF-alpha inhibitor | Inhibitor | Fanconi anemia | 0.5 | Completed |


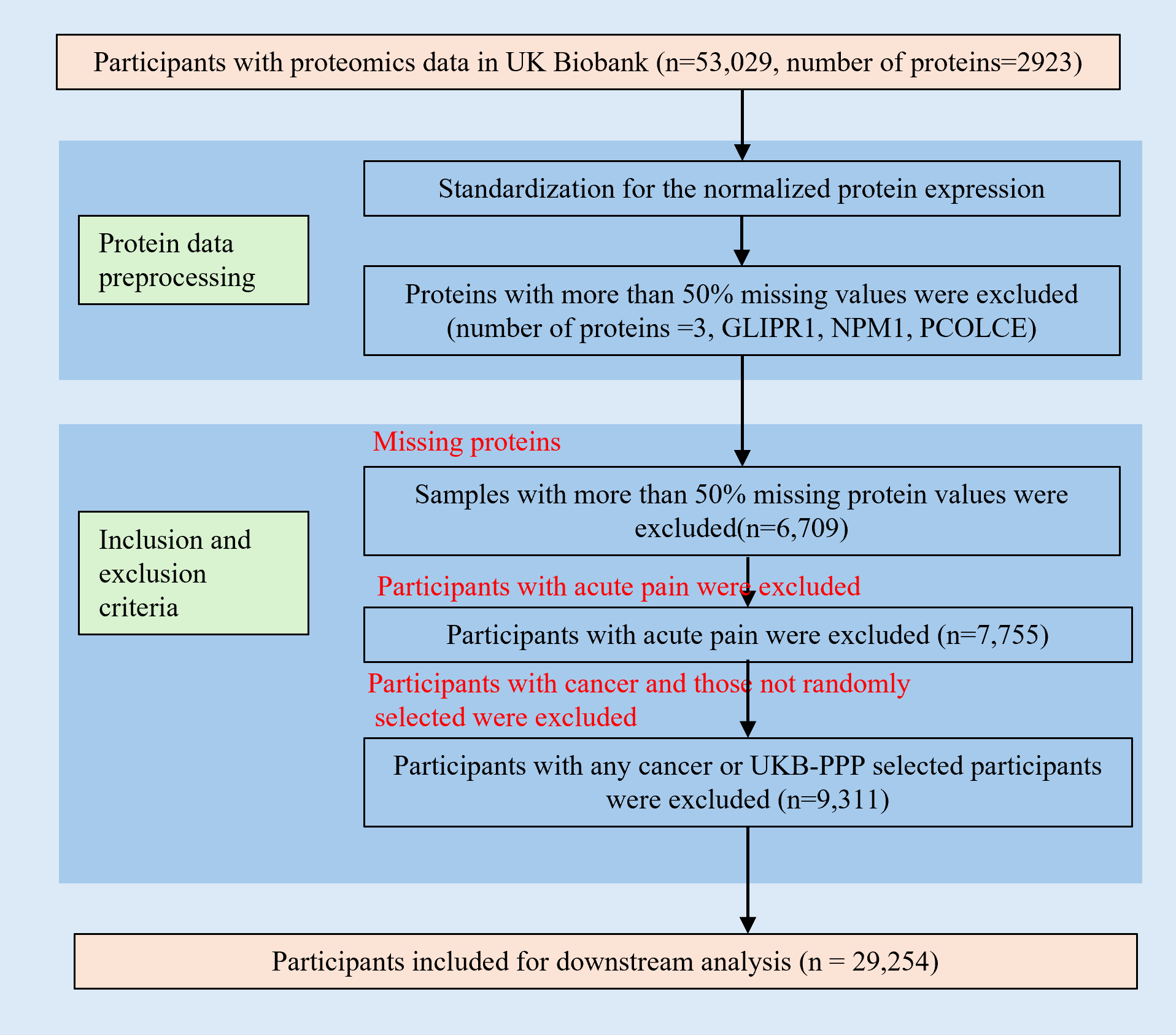


**Figure S1. Flowchart of sample and proteins selection pipeline.**

Notes: This cohort study is based on a random subset of UK Biobank individuals (N=53,029). Three proteins (GLIPR1, Glioma pathogenesis-related protein 1; NPM1, Nucleophosmin; PCOLCE, Procollagen C-endopeptidase enhancer 1) were excluded. Participants with acute pain were excluded from the main analysis.


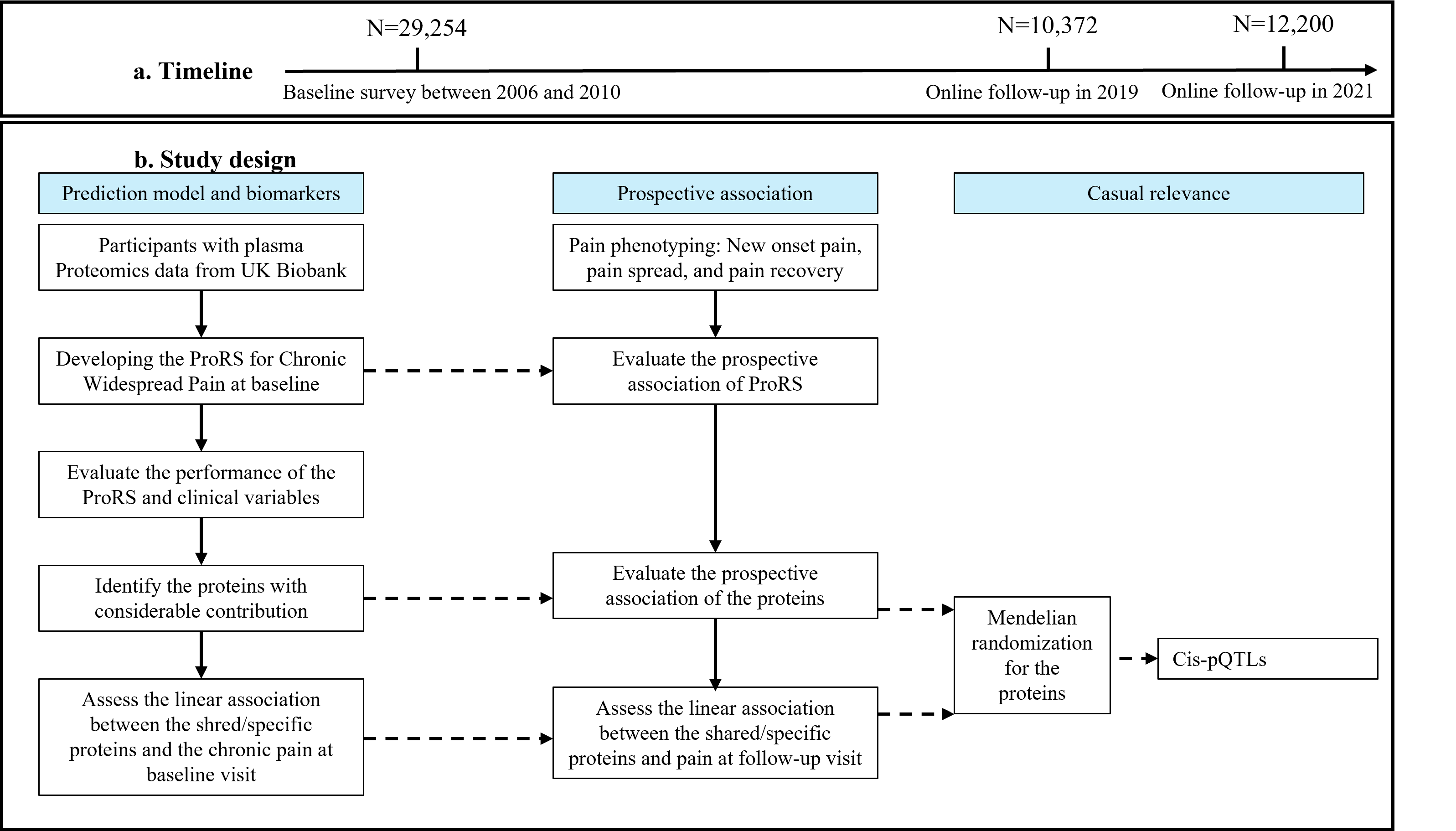


**Figure S2 Study design and timeline.**

Notes: a. The baseline survey was conducted between 2006 and 2010, and the online follow-up survey was conducted in 2010. The number of participants at the baseline visit was 29,254 , and the number of participants at the follow-up visit was 10,374 and 12,200.

b. The proteins significantly associated with overall chronic pain and chronic pain types after Bonferroni correction were selected to generate the proteomic risk score for overall chronic pain and chronic pain types. The contribution of each protein to overall chronic pain and chronic pain types was evaluated based on relative importance. These proteins with considerable contributions to the chronic pain types were reordered to identify the shared proteins associated with all chronic pain types and the specific proteins associated with specific chronic pain types. The linear or non-linear association between the shared/specific proteins and chronic pain and its subtypes was assessed. The longitudinal cohort of chronic pain was used to evaluate the prospective association between the proteomic risk score and the new onset of chronic pain, chronic pain types, pain spread, and pain recovery. The linear and non-linear association between the shared/specific proteins and the new onset, spreading, and recovery of chronic pain was also assessed in the longitudinal cohort. The causal relationship between the shared/specific proteins and multisite chronic pain was evaluated using Mendelian randomization. The genetic instrument included cis-SNPs.


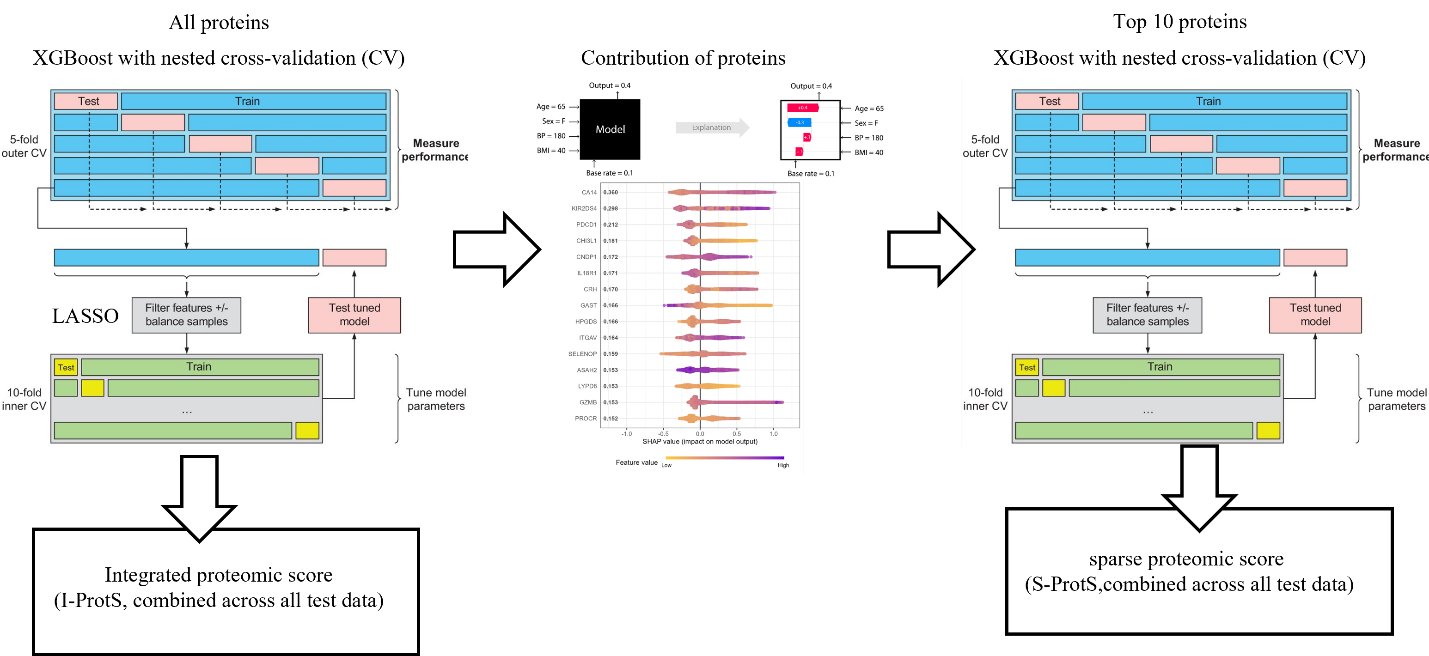


**Figure S3. Flowchart of developing the proteomics risk scores pipeline.**


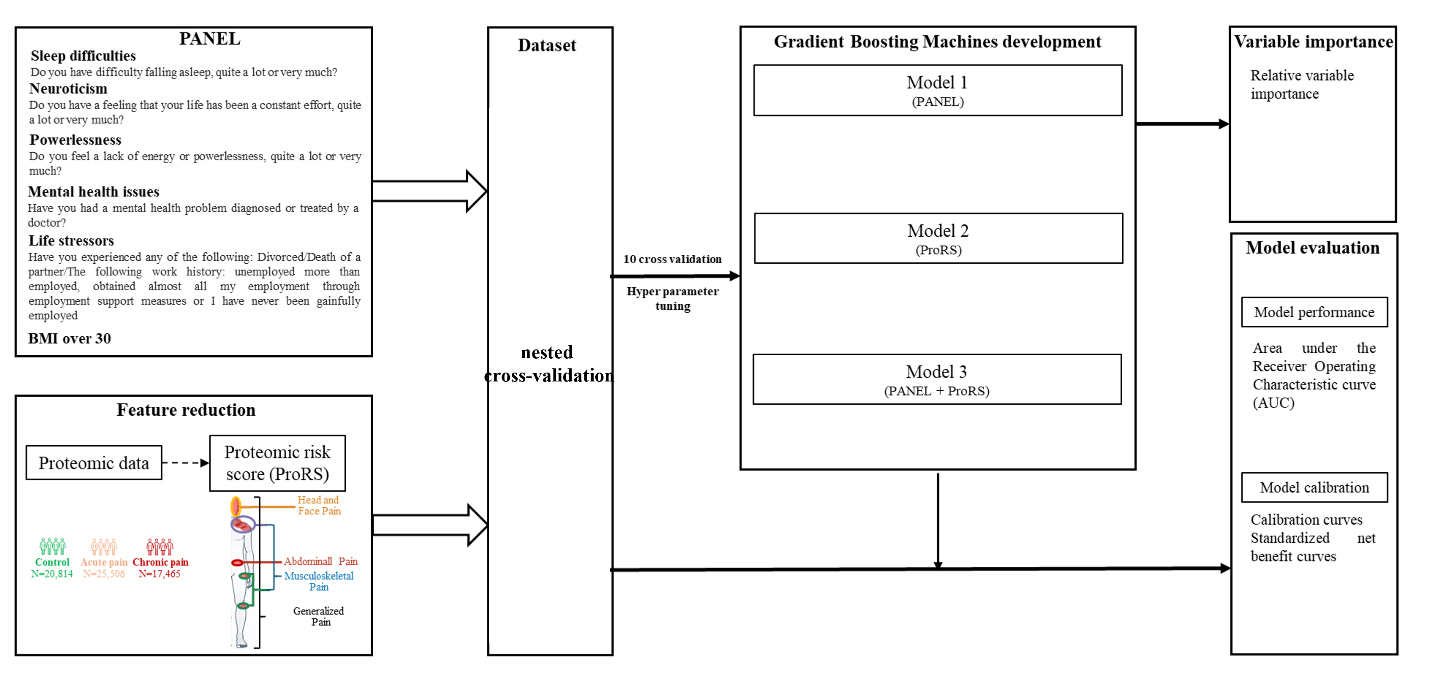


**Figure S4 Flowchart of developing the gradient boosting model with different variables sets.**


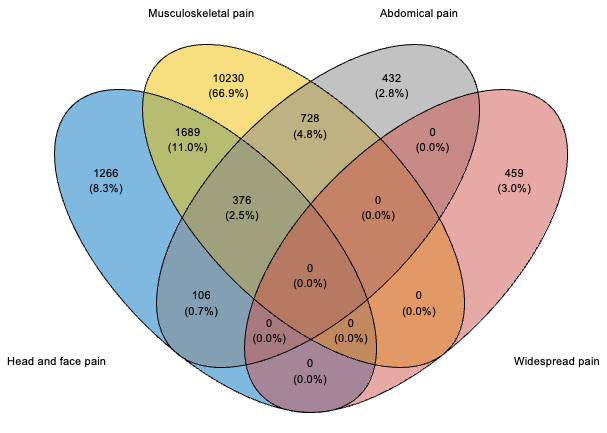


**Figure S5**. Overlap among chronic pain types at baseline


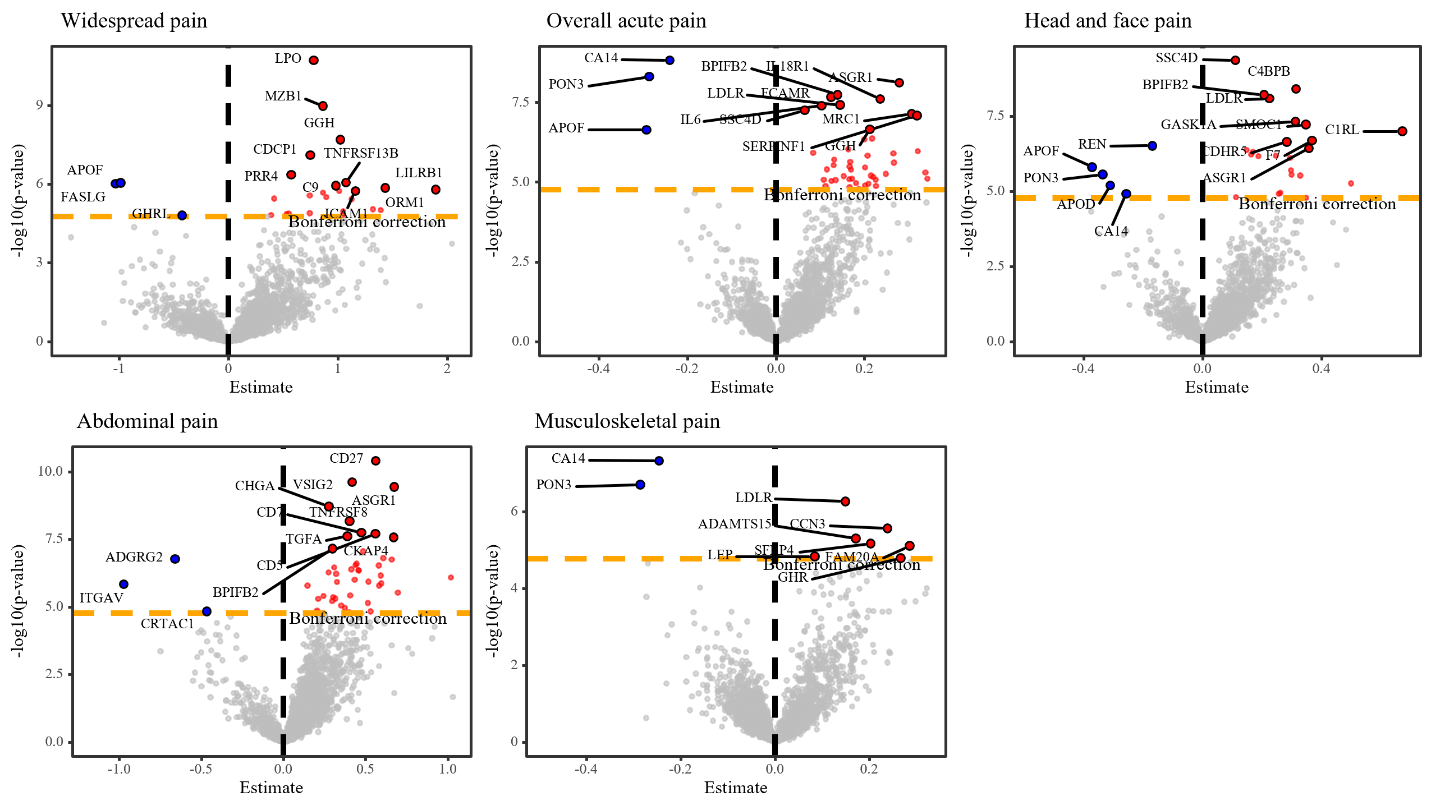


**Figure S6** Volcano Plot for Overall and Specific Acute Pain


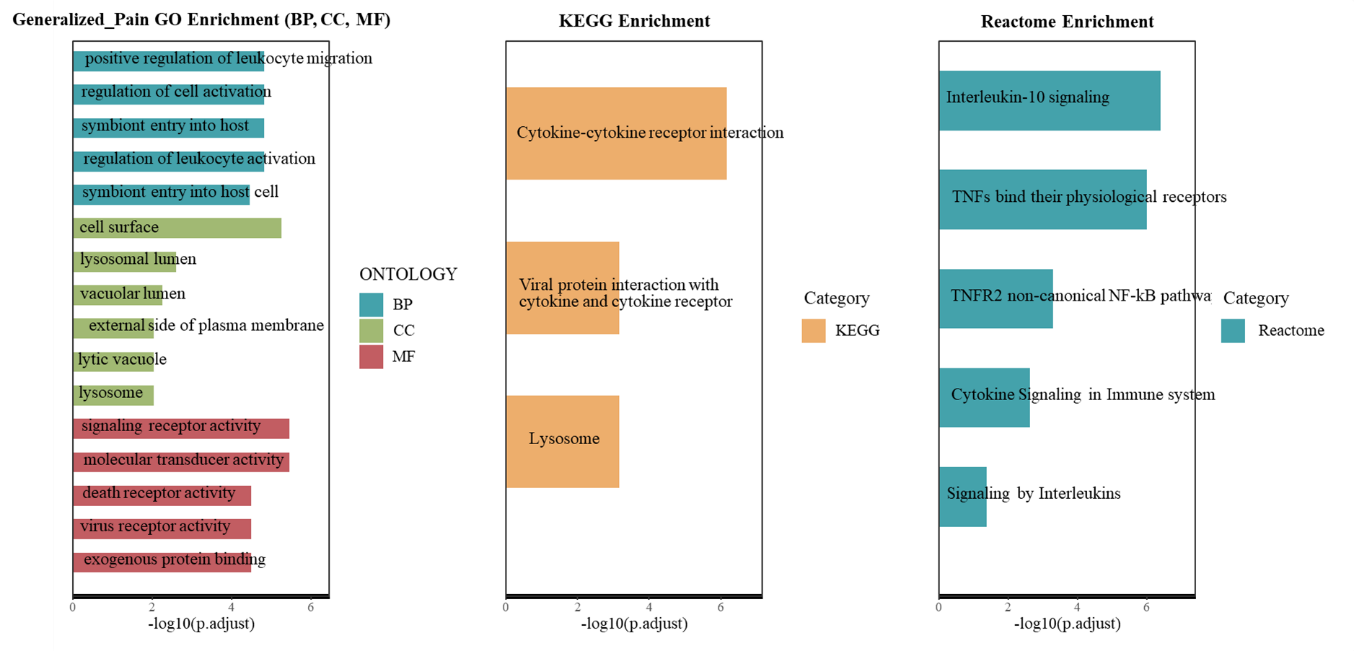


**Figure S7** Enrichment of the proteins associated with chronic widespread pain.


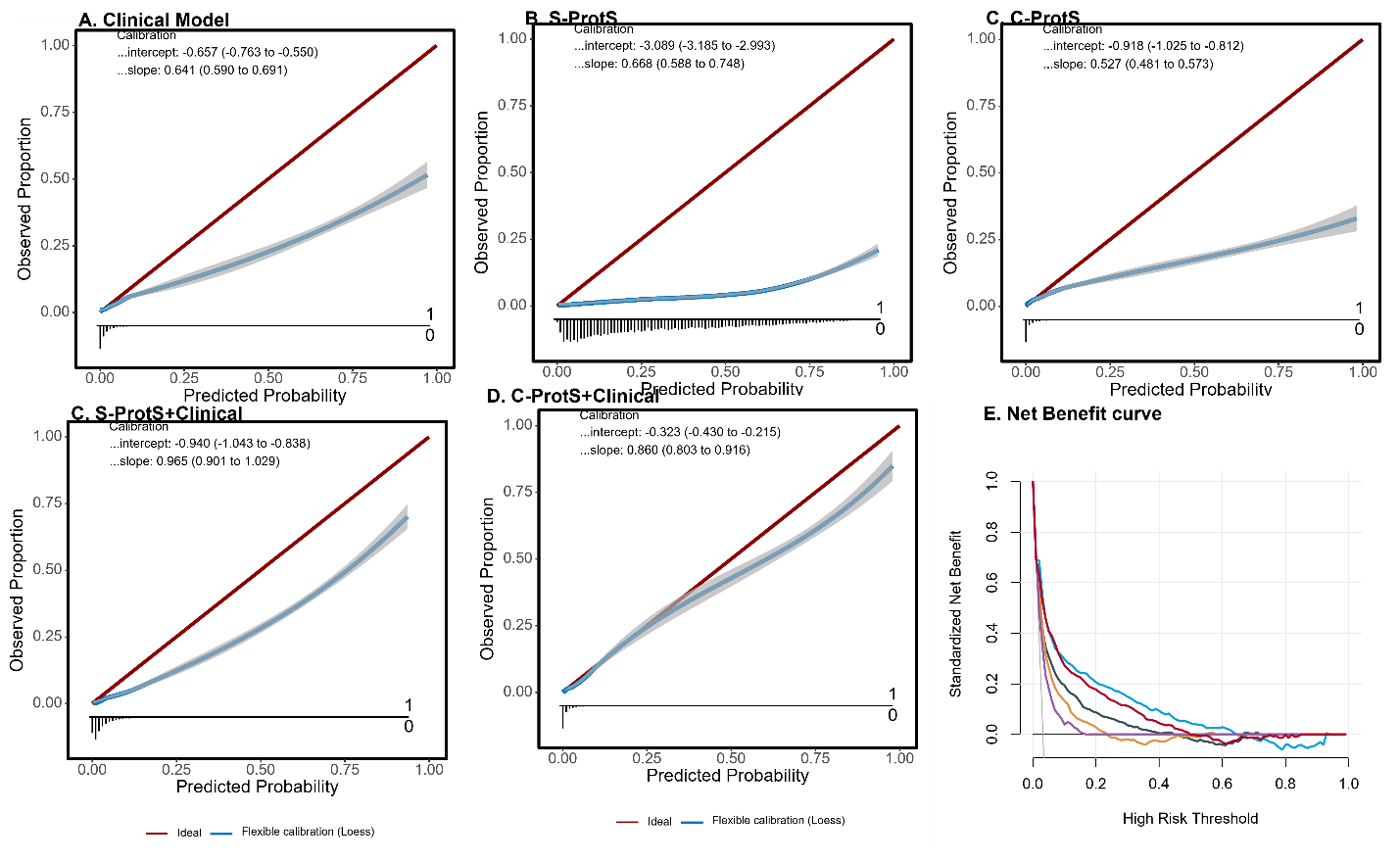


**Figure S8**. Model performance and calibration of proteomic score and clinical predictors


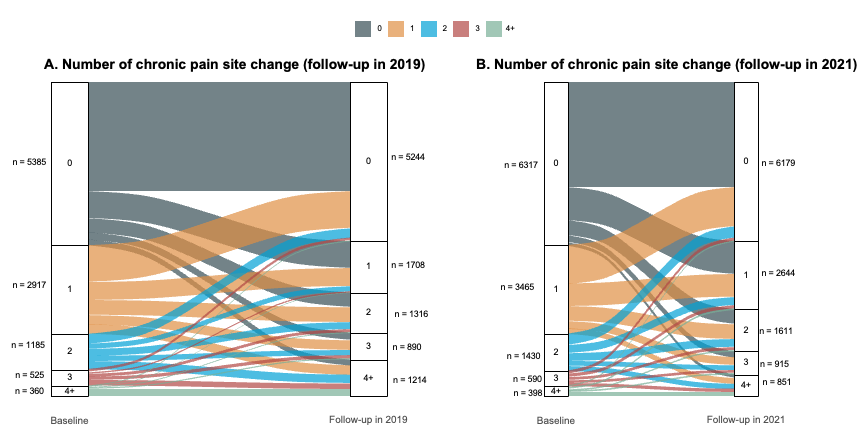


**Figure S9. Changes in the Number of Chronic Pain Sites Between Baseline and Follow-Up Visits**

**
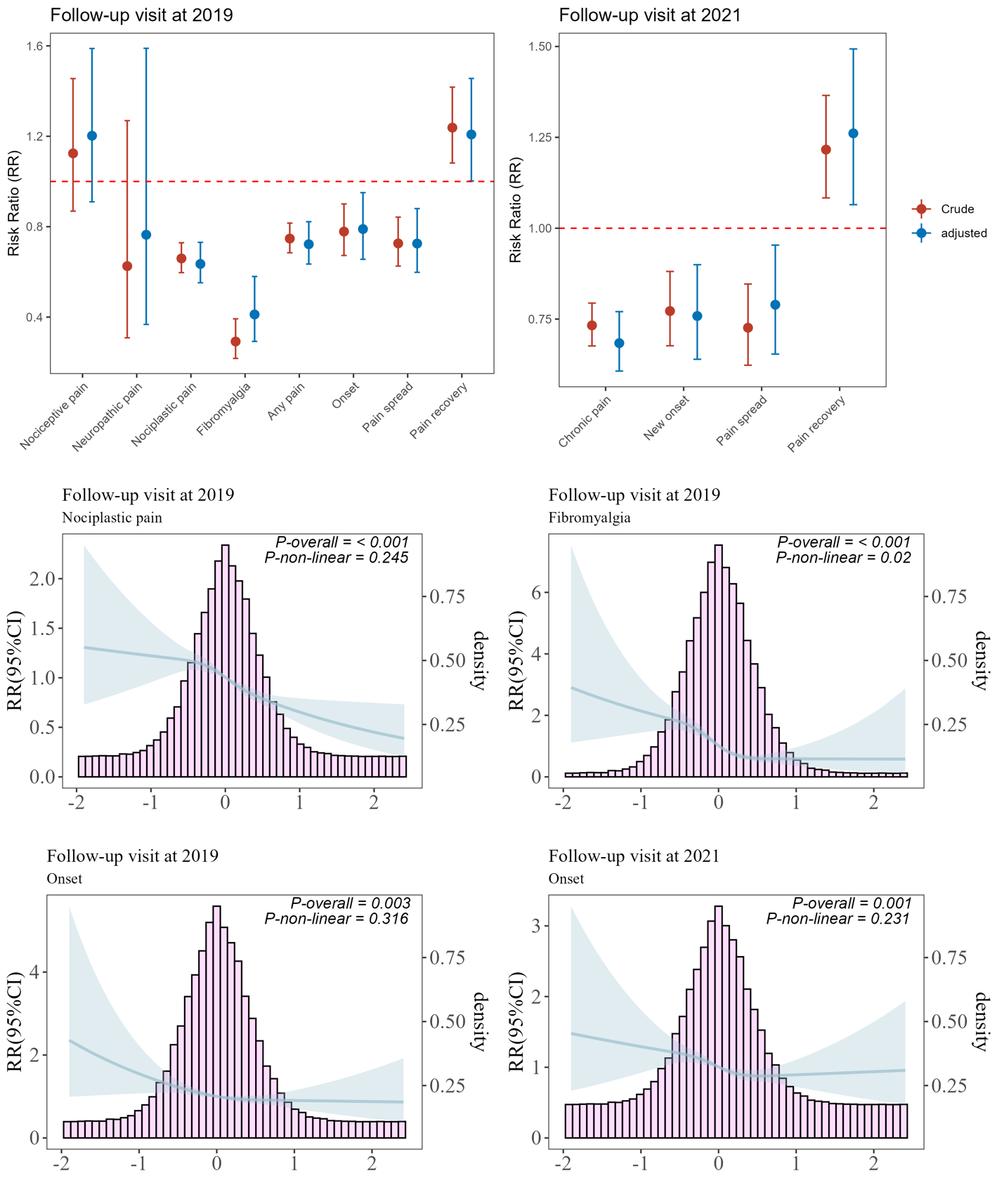
**

**Figure S10. The perspective association of CA14 and chronic pain.**


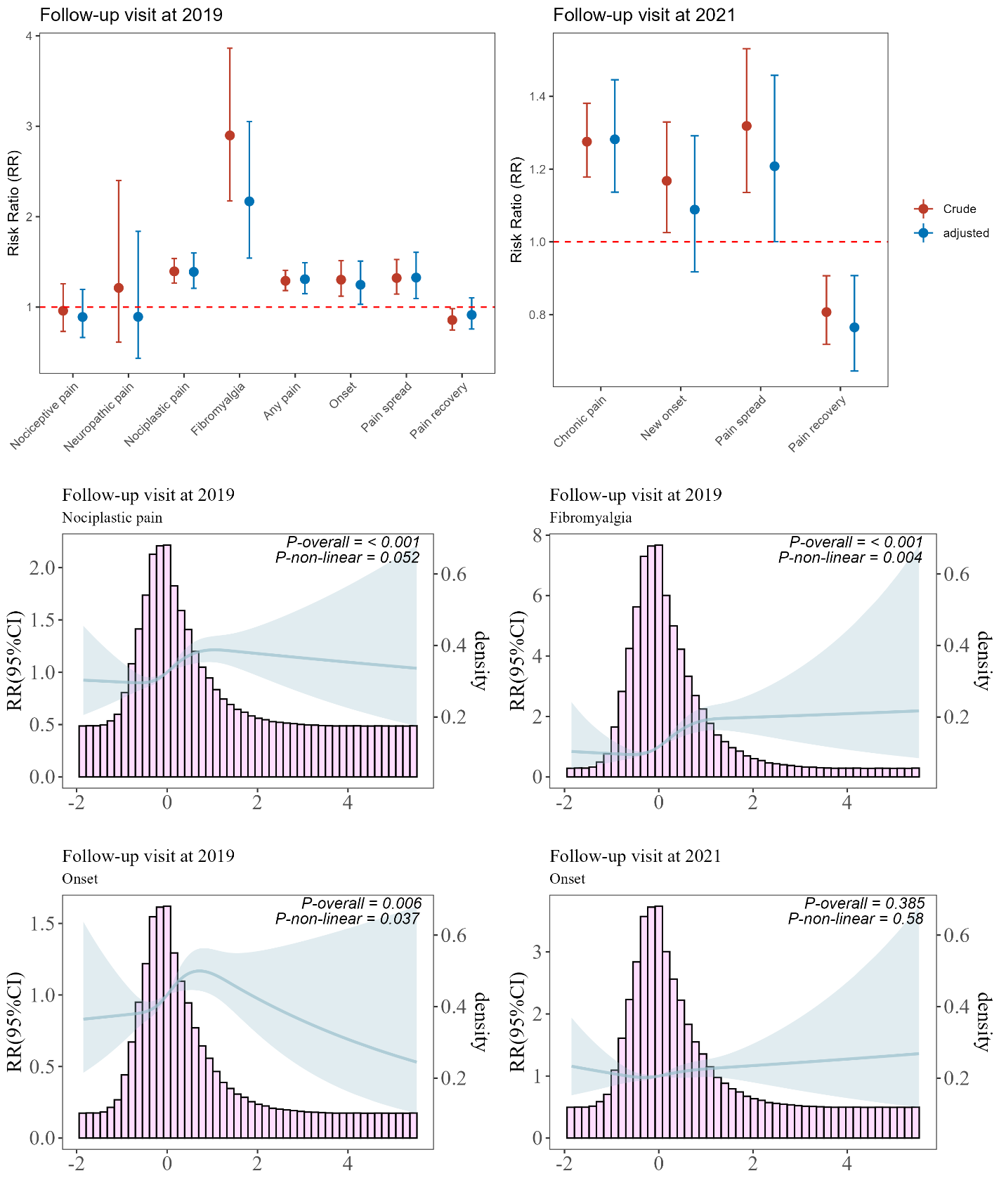


**Figure S11. The perspective association of BPIFB2 and chronic pain.**

**
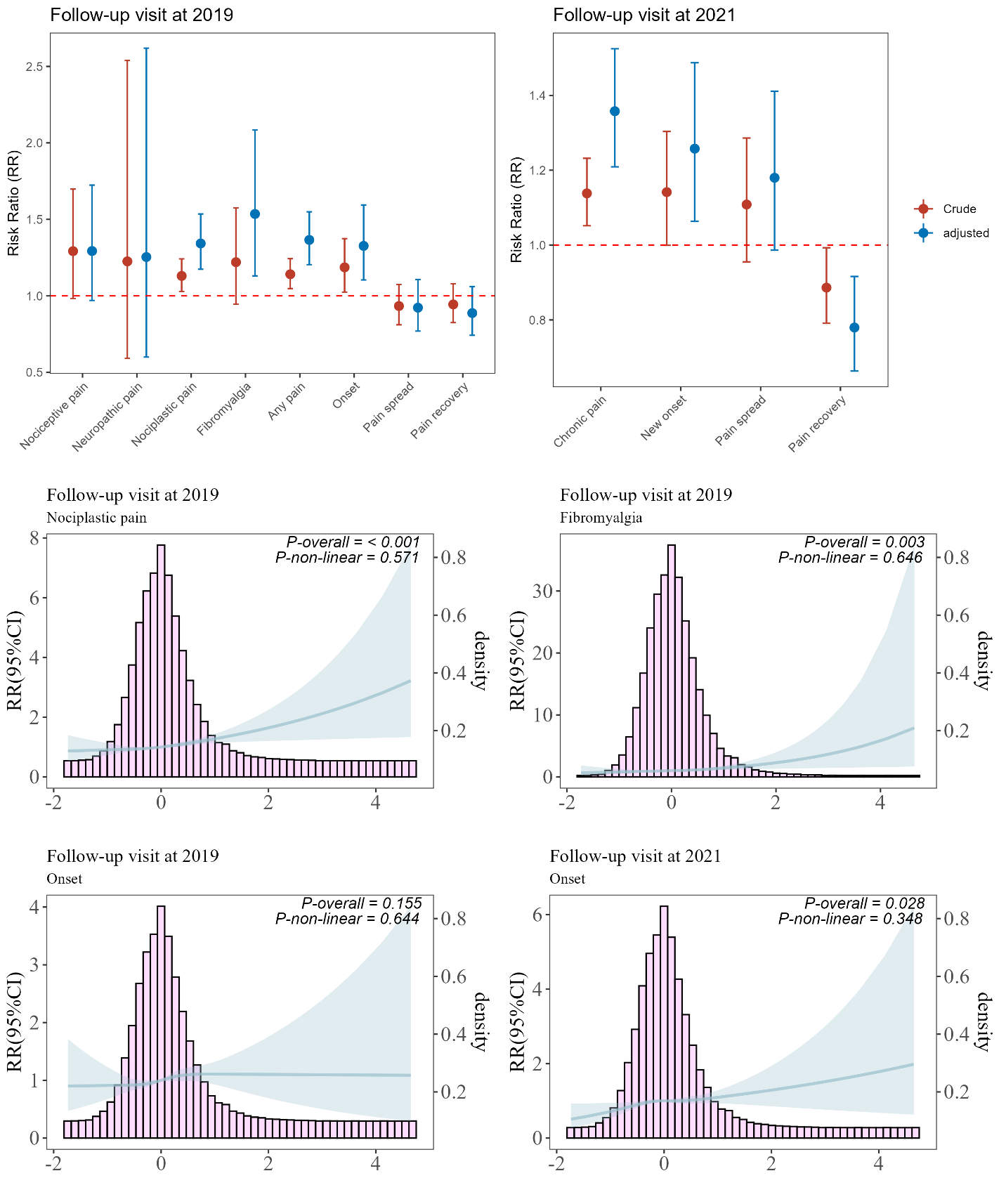
**

**Figure S12. The perspective association of COL9A1 and chronic pain.**


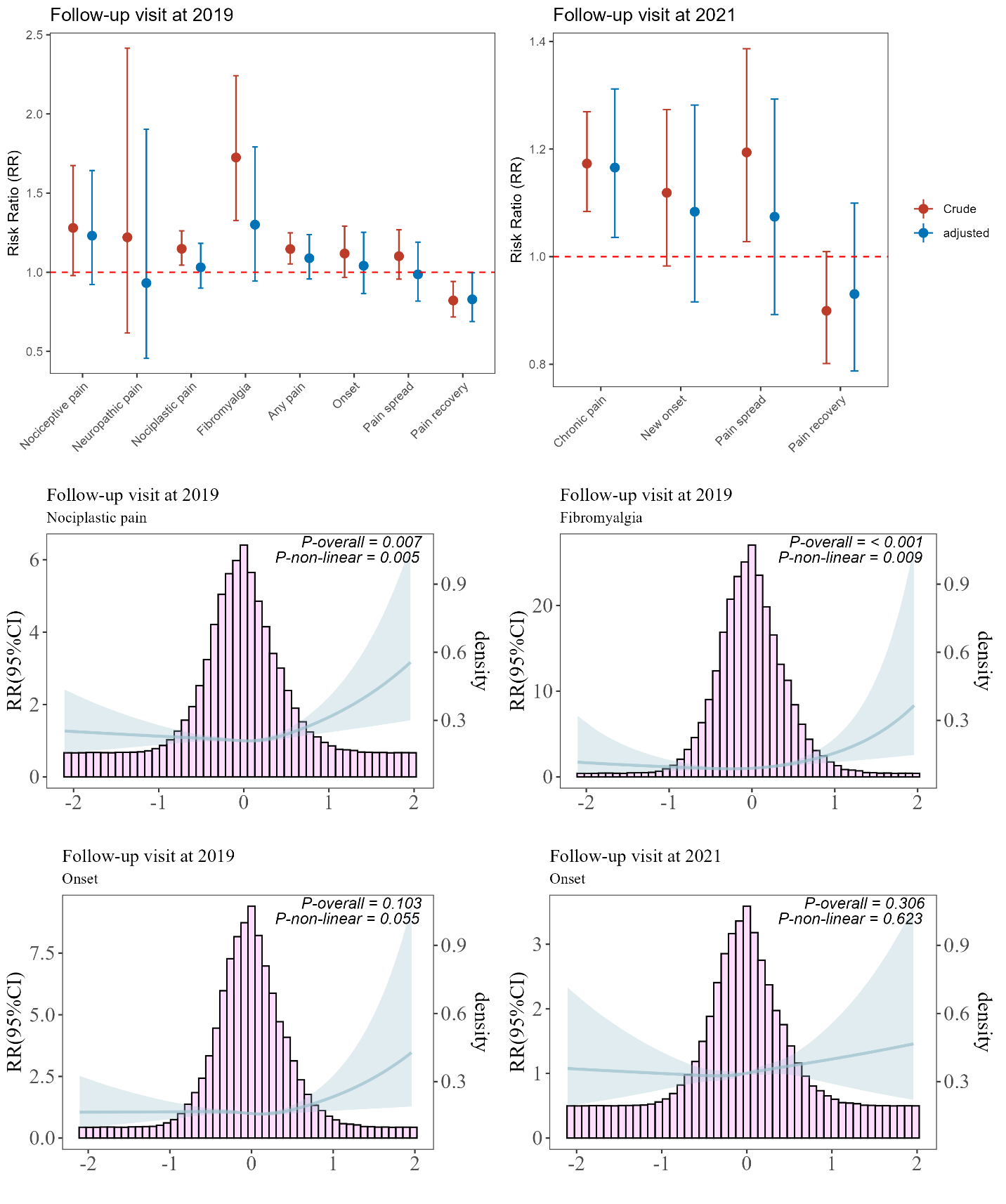


**Figure S13. The perspective association of CRELD1 and chronic pain.**

**
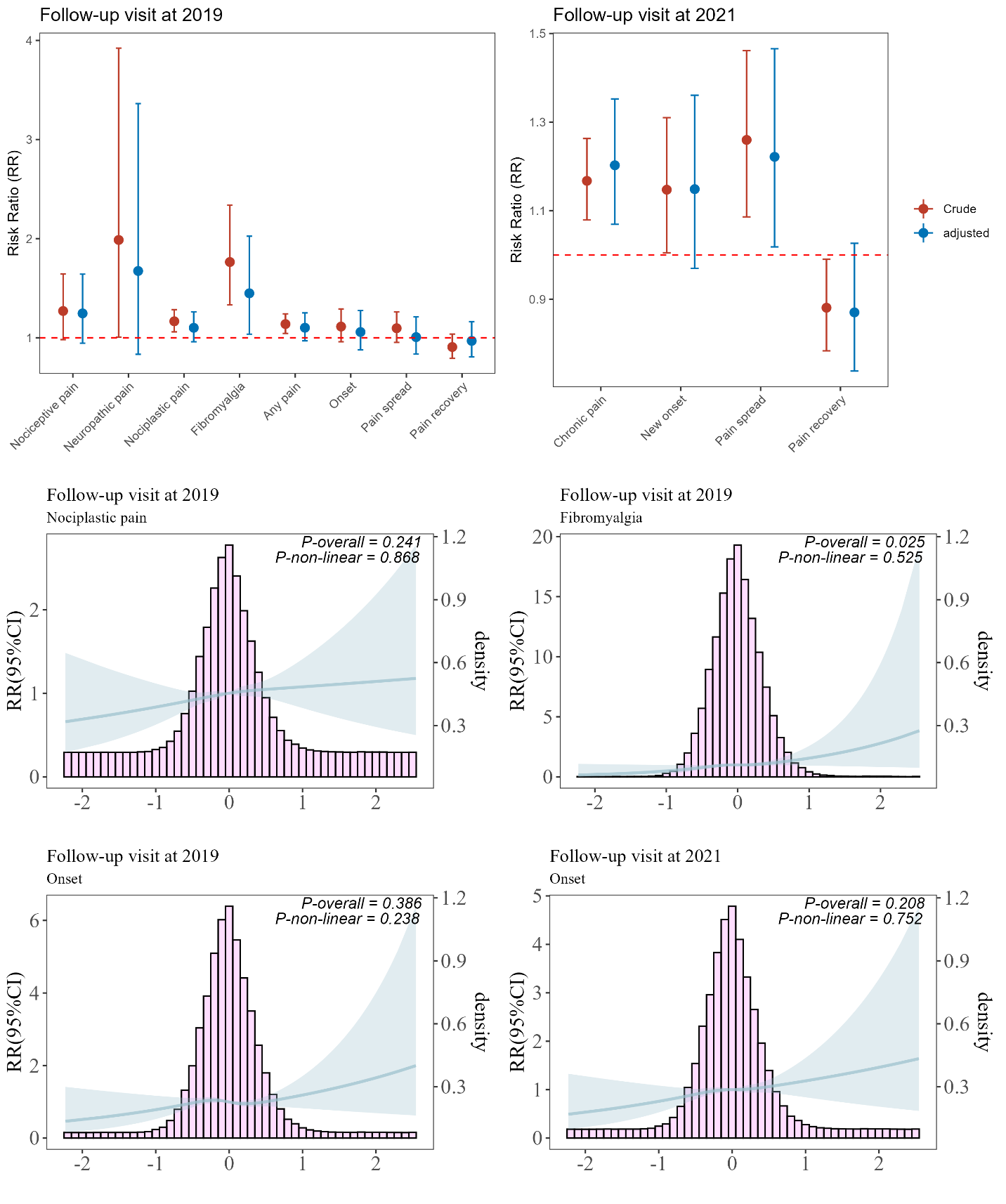
**

**Figure S14. The perspective association of CTSO and chronic pain.**

**
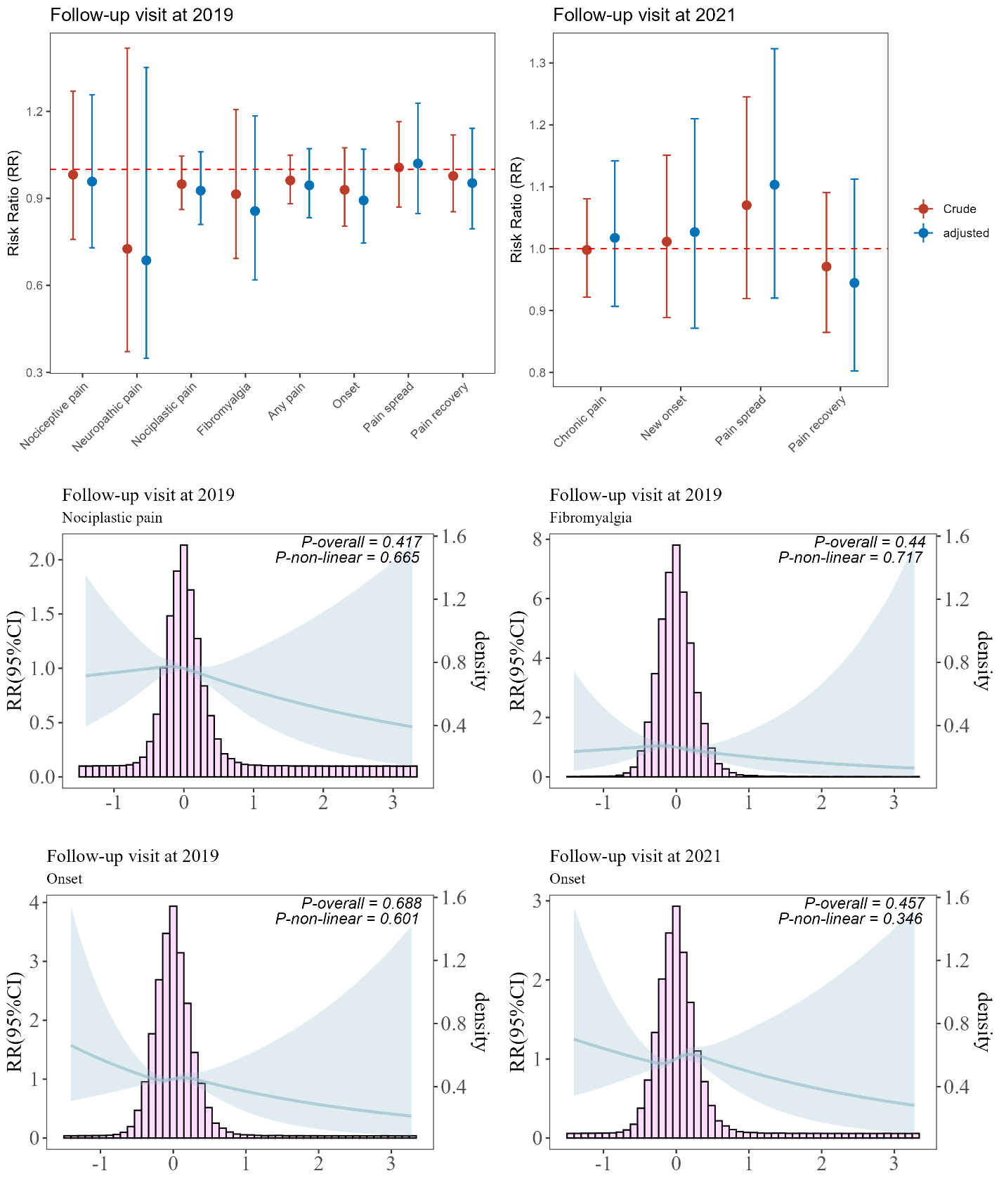
**

**Figure S15. The perspective association of DDR1 and chronic pain.**

**
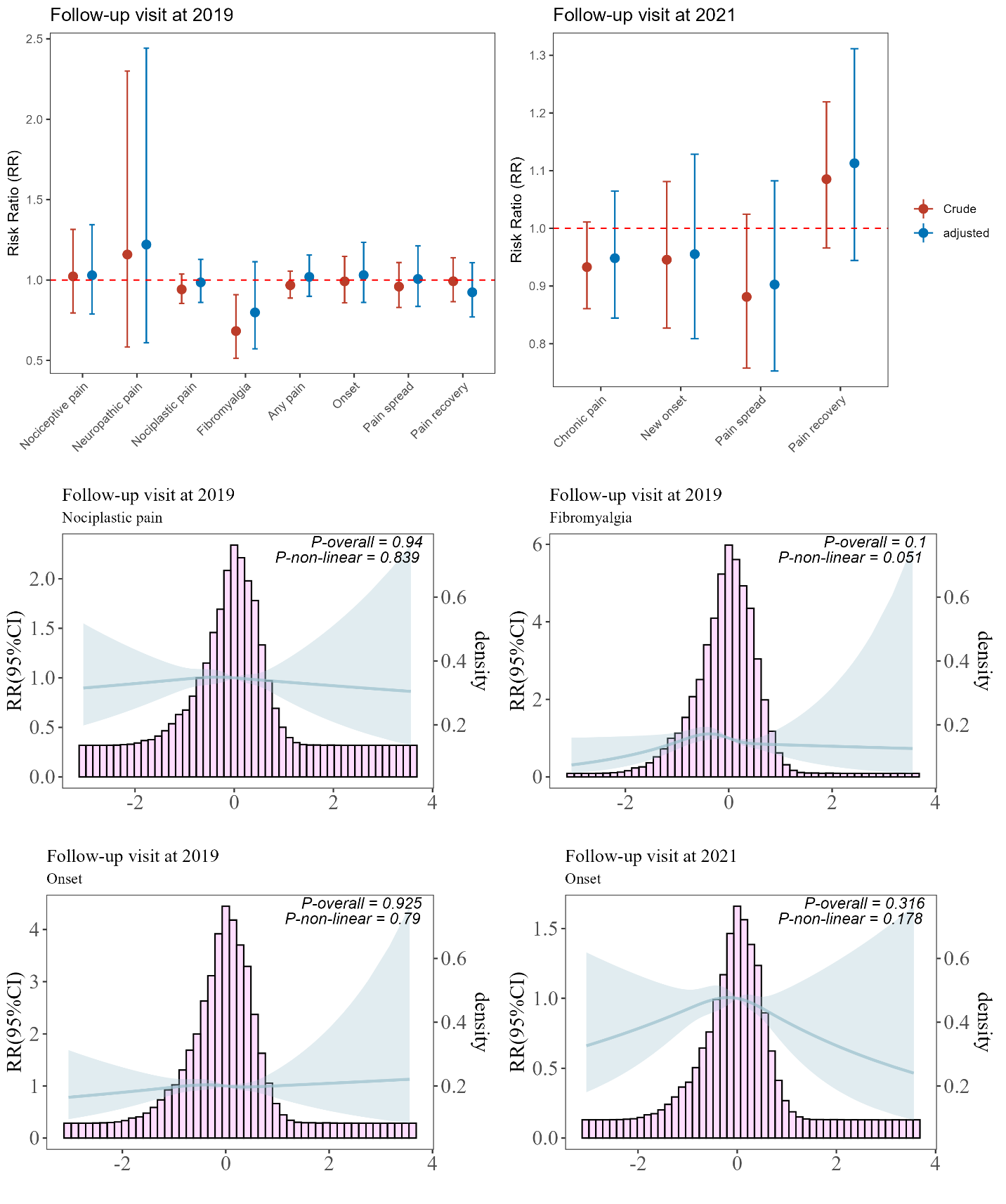
**

**Figure S16. The perspective association of DPEP1 and chronic pain.**

**
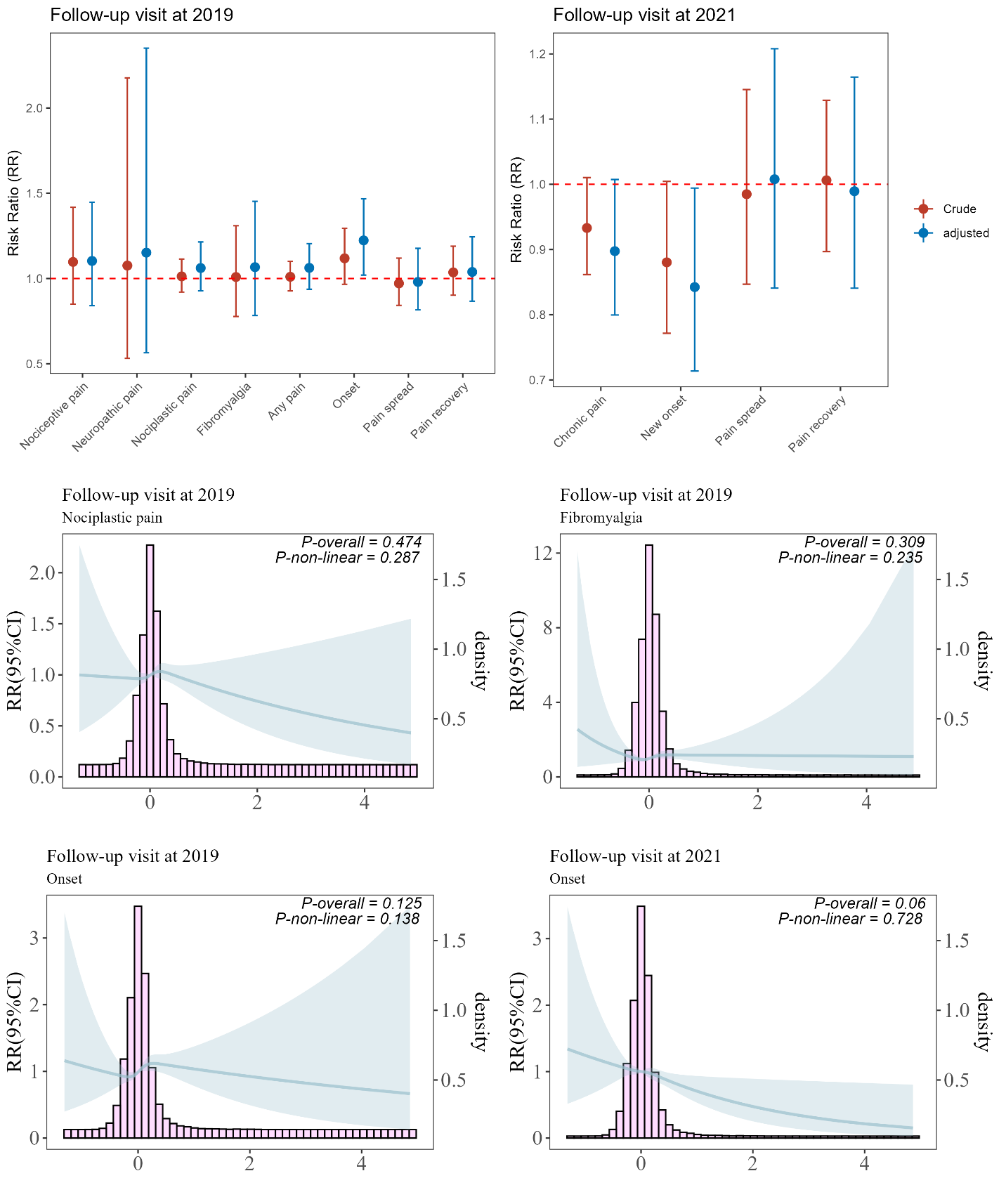
**

**Figure S17. The perspective association of FAM171B and chronic pain.**

**
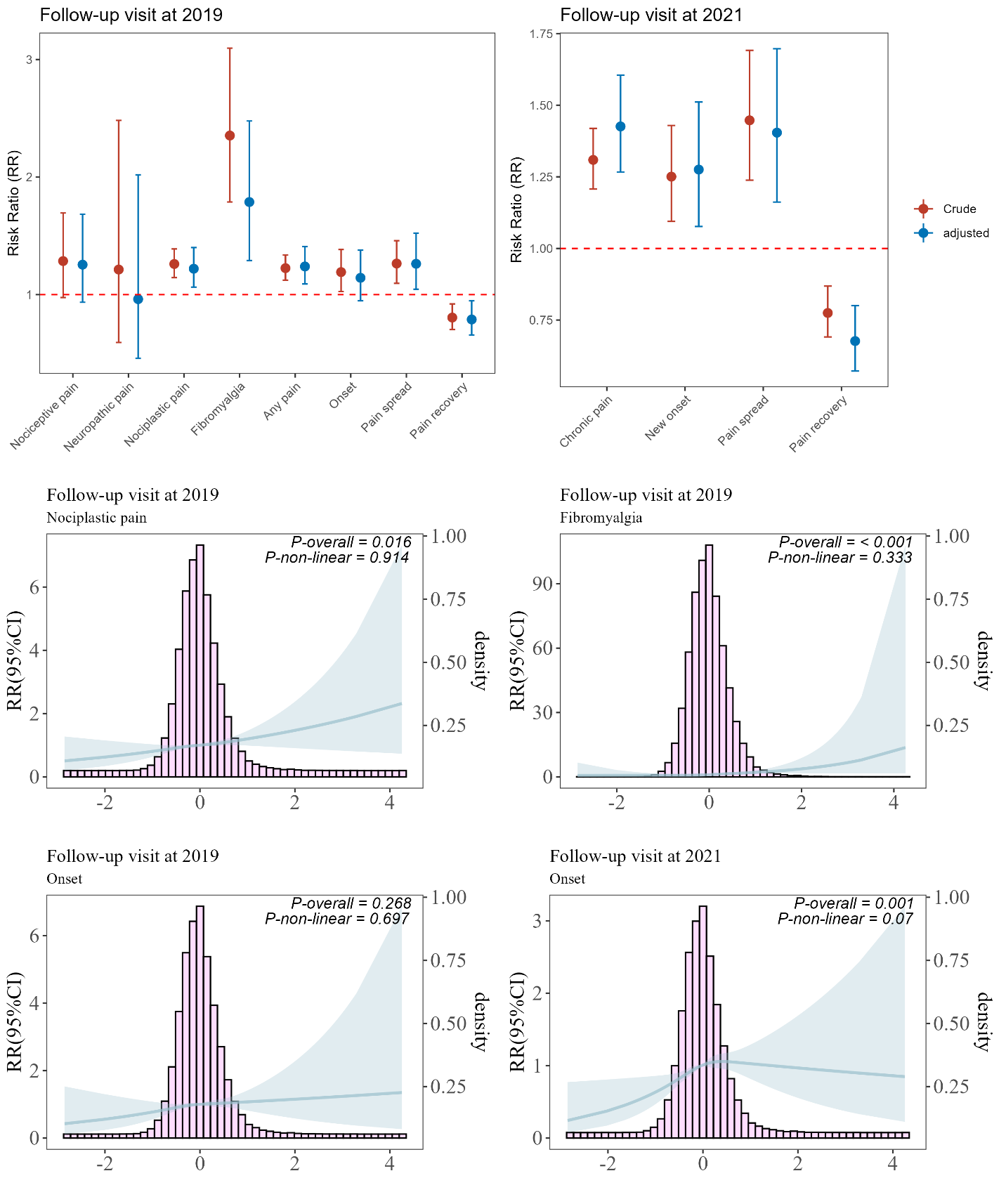
**

**Figure S18. The perspective association of IFI30 and chronic pain.**

**
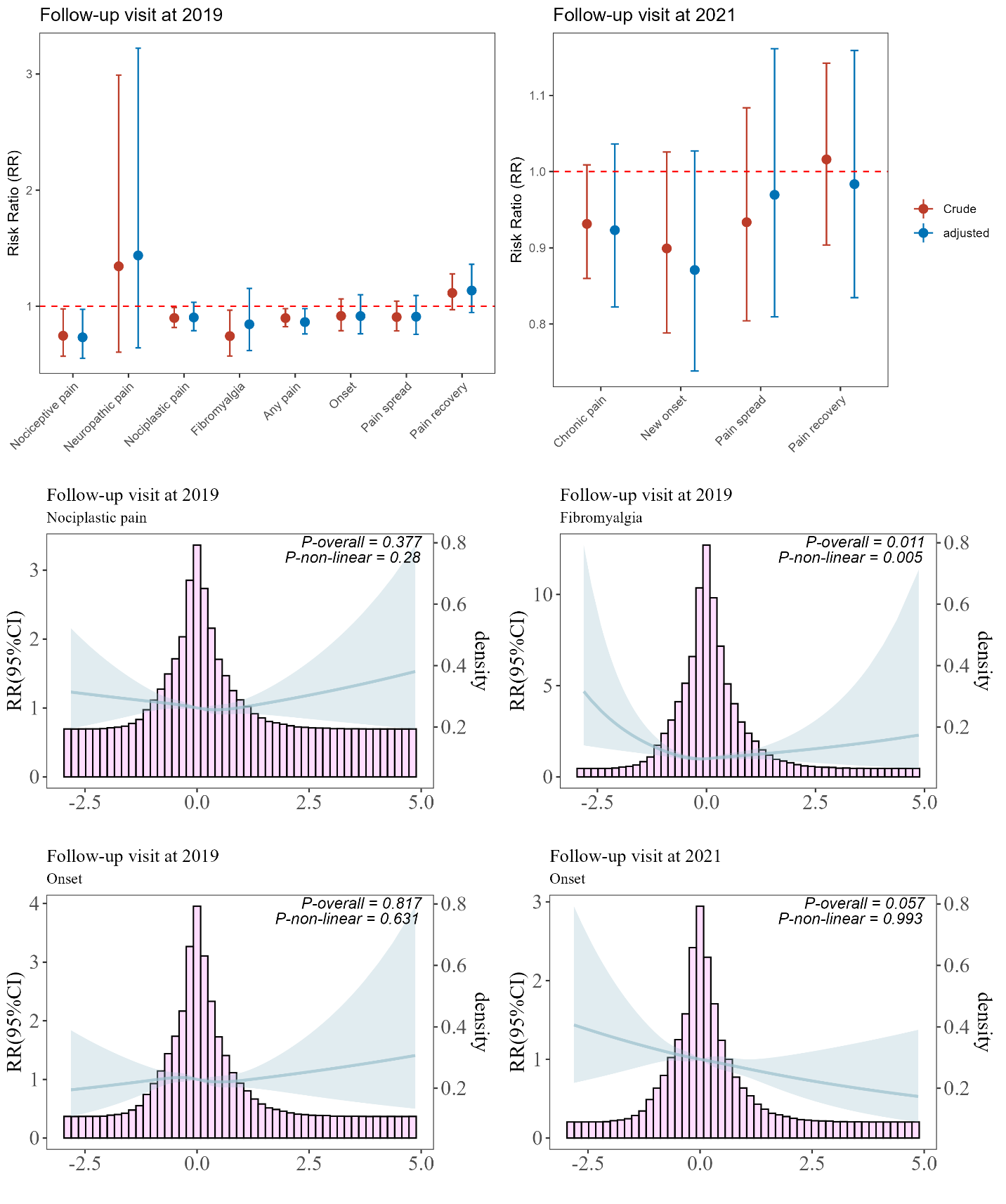
**

**Figure S19. The perspective association of LEG1 and chronic pain.**

**
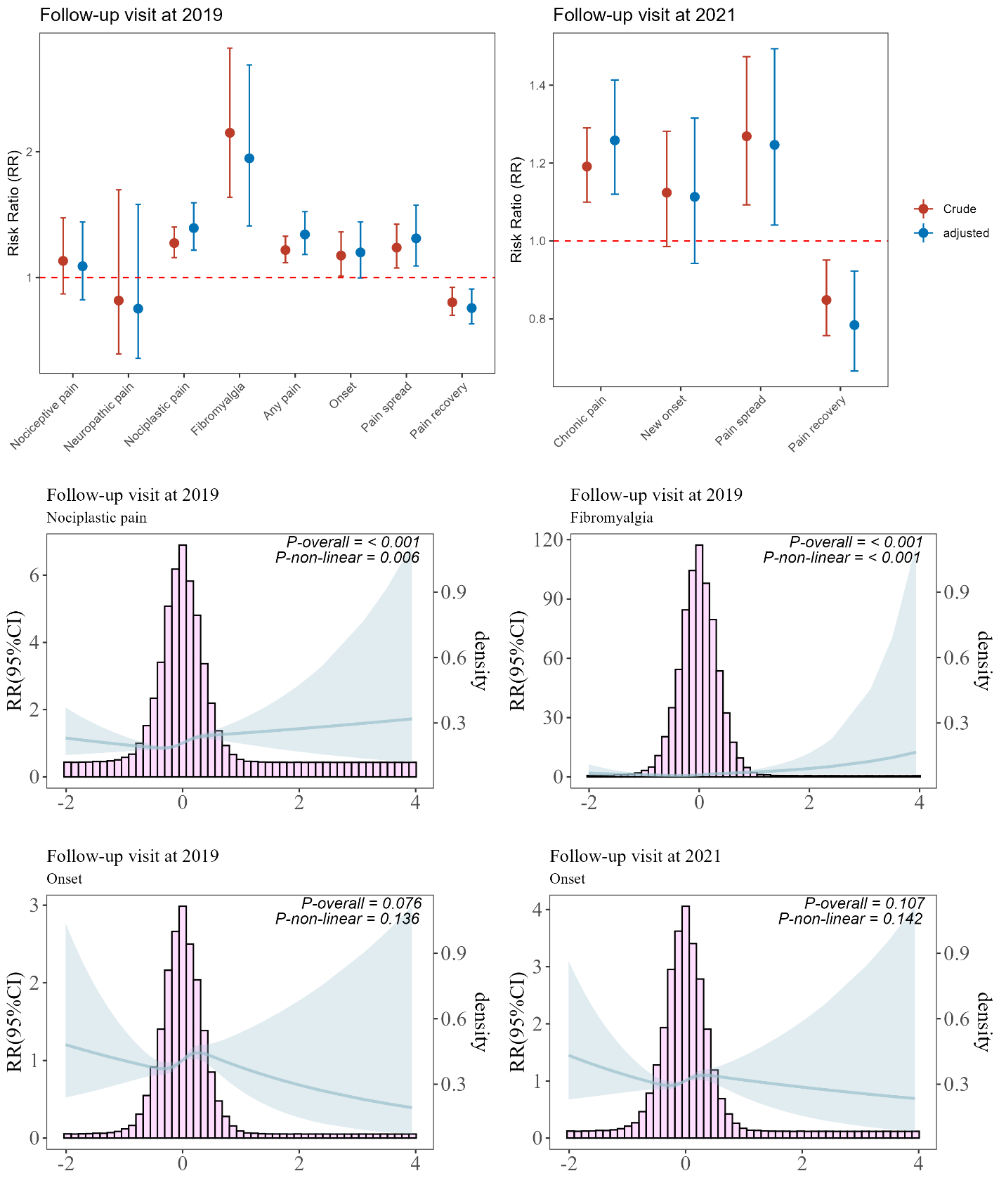
**

**Figure S20. The perspective association of LGALS3 and chronic pain.**

**
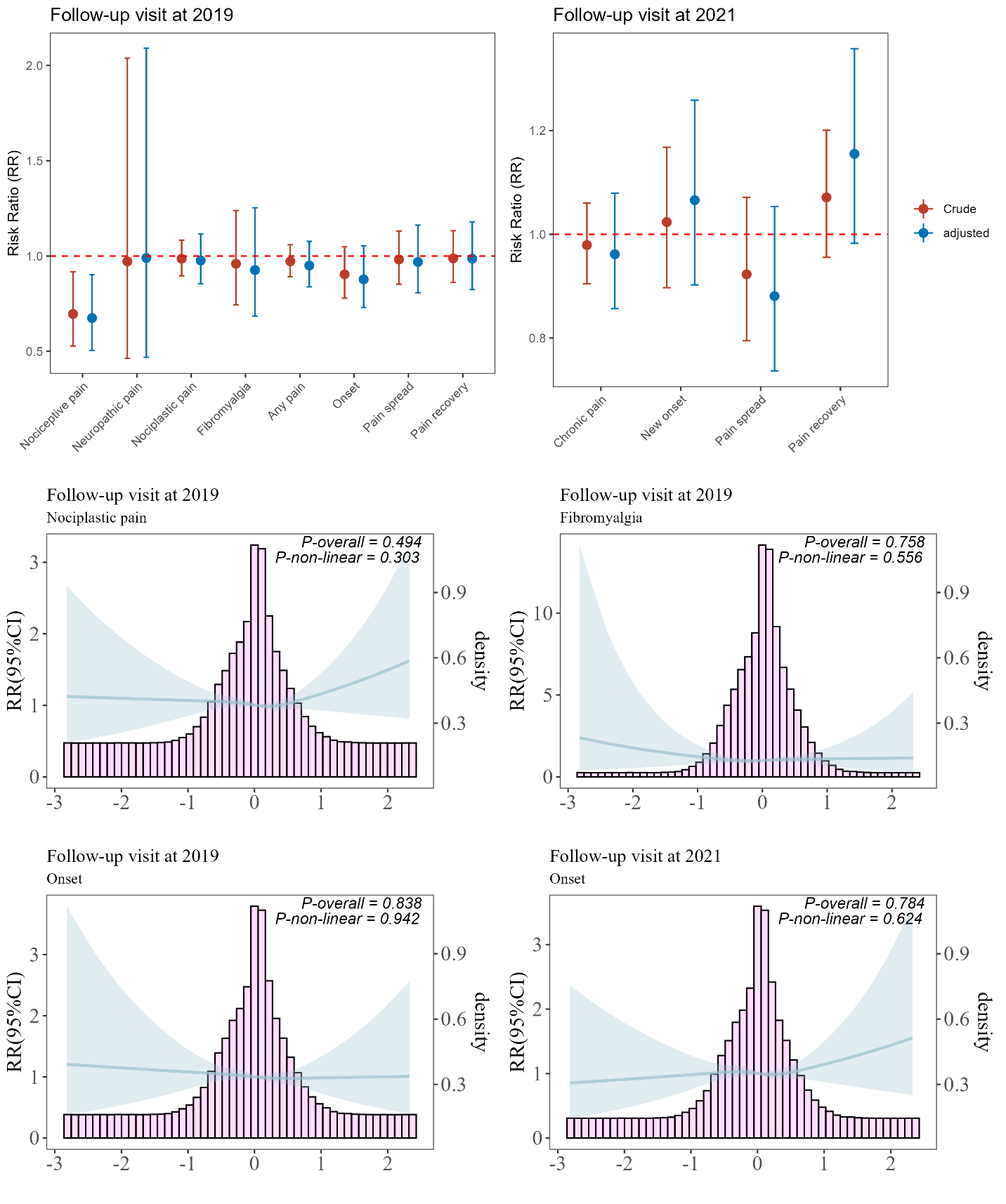
**

**Figure S21. The perspective association of LRRC37A2 and chronic pain.**

**
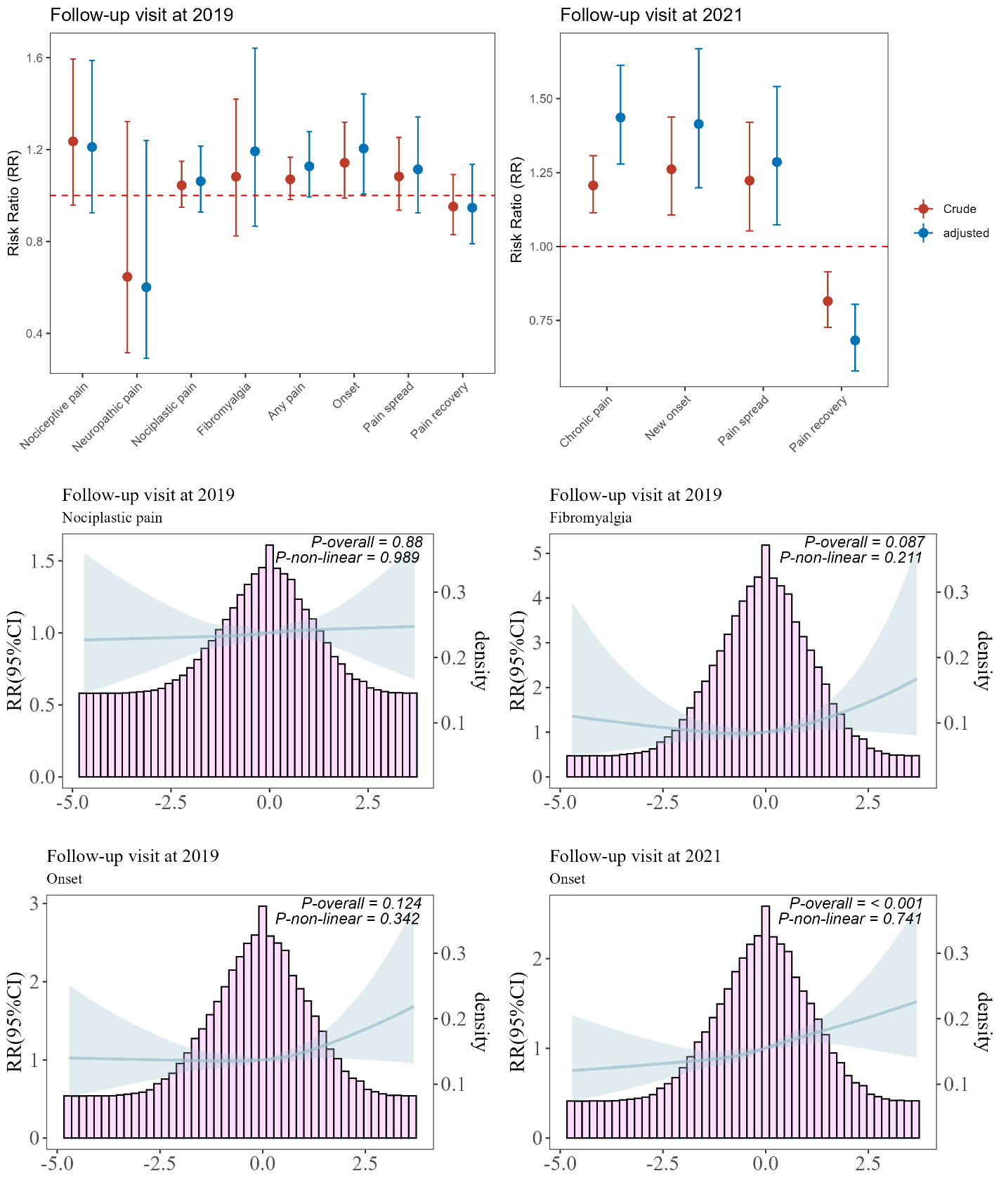
**

**Figure S22. The perspective association of MLN and chronic pain.**

**
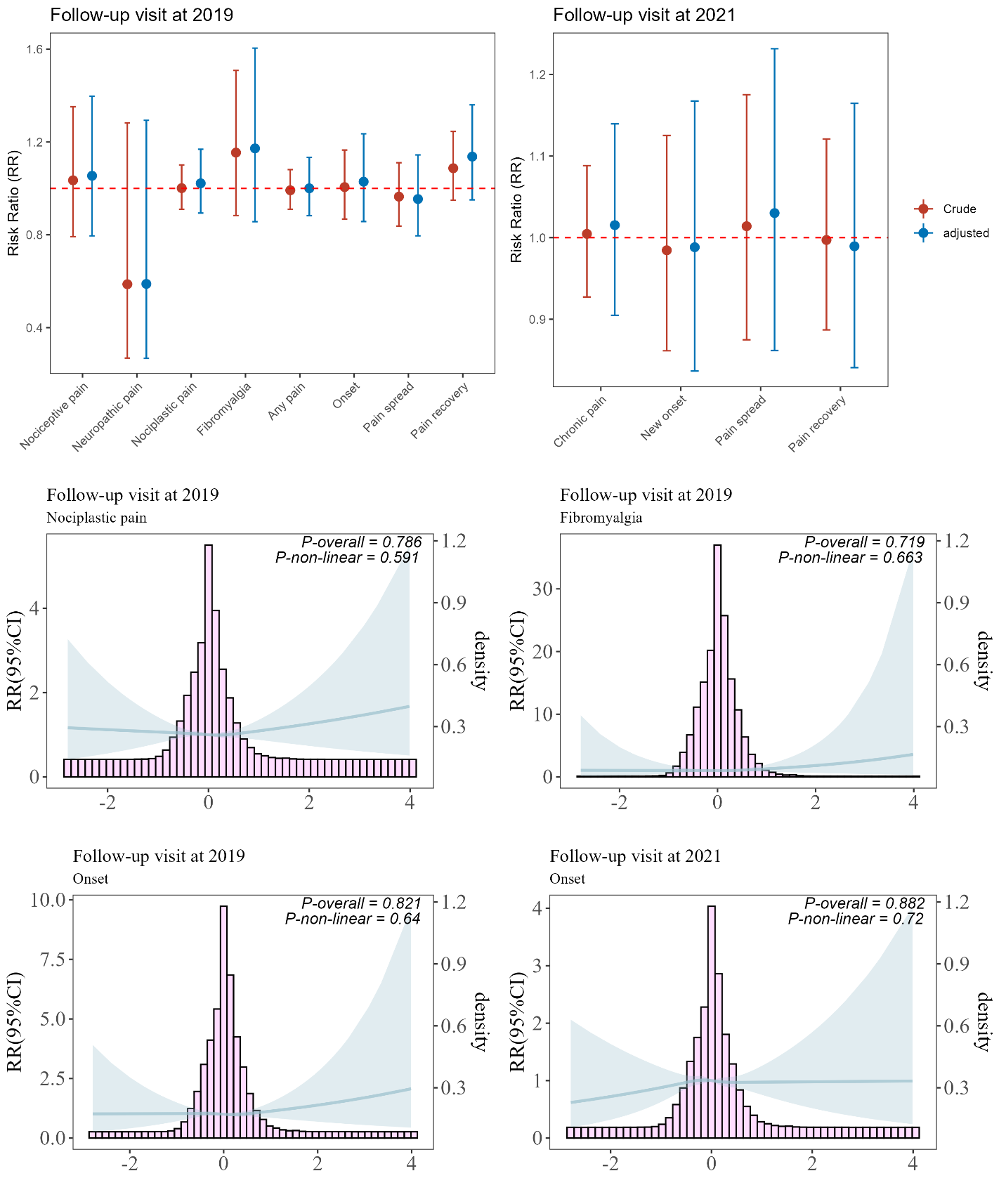
**

**Figure S23. The perspective association of PRSS53 and chronic pain.**

**
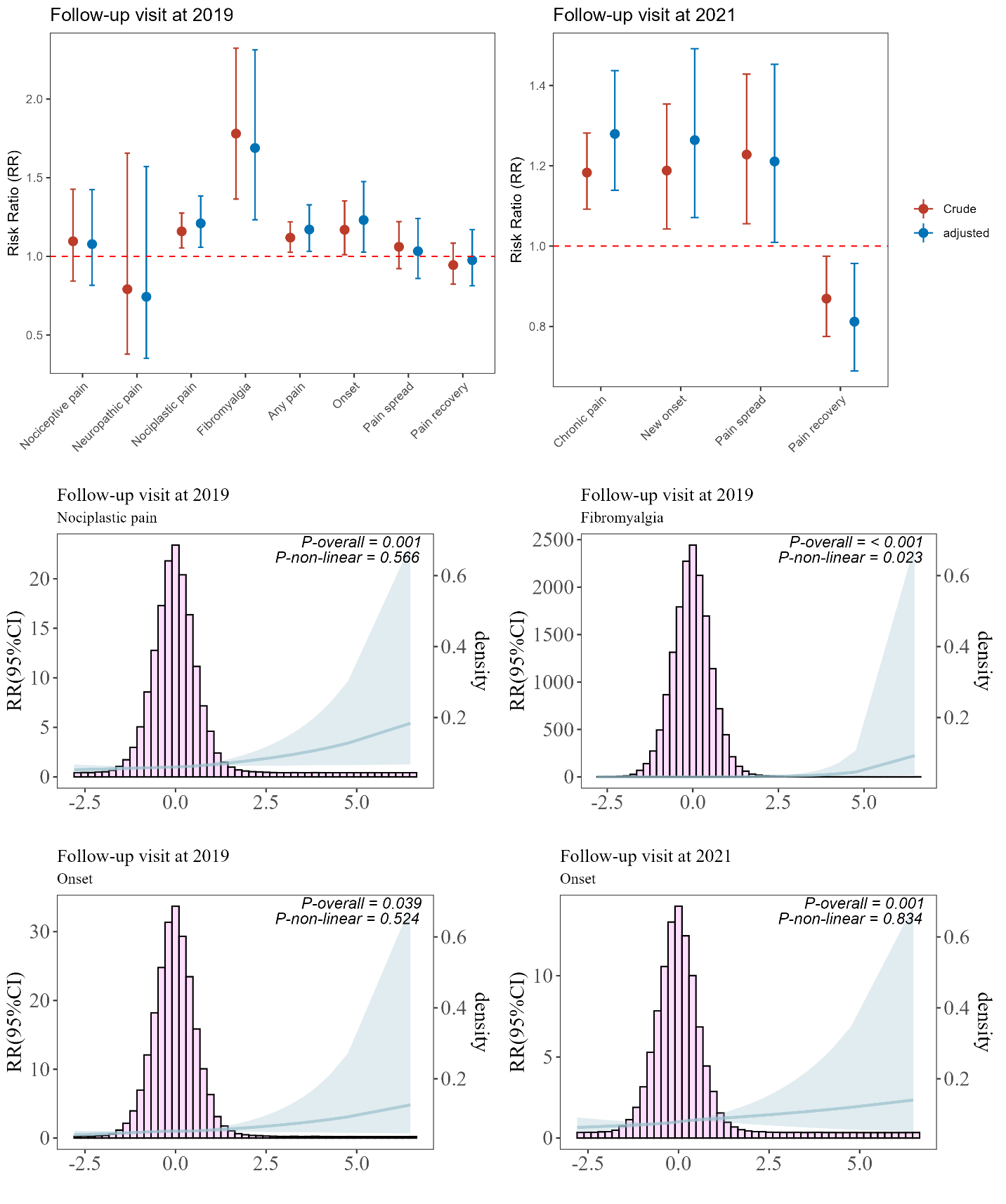
**

**Figure S24. The perspective association of PTN and chronic pain.**

**
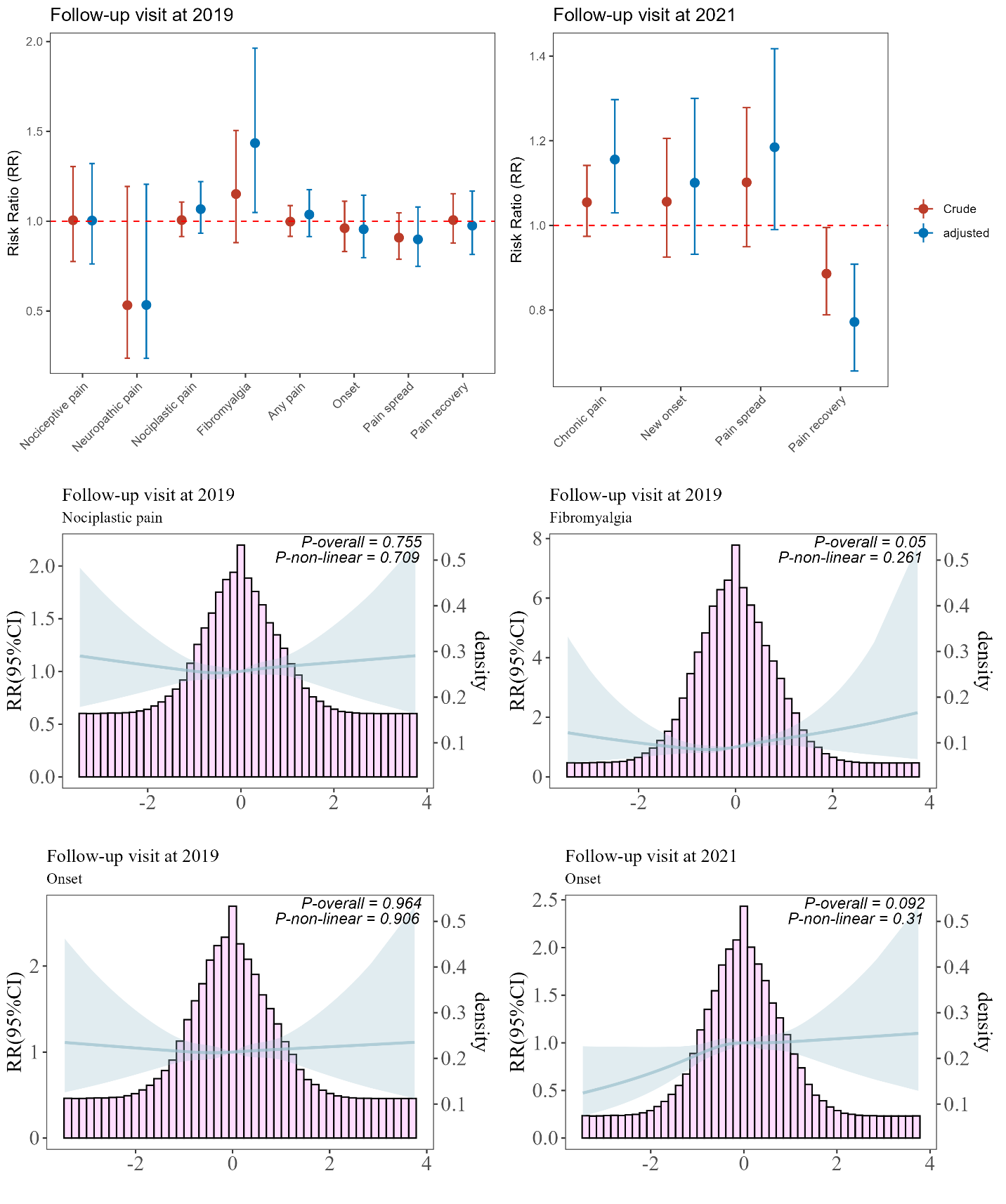
**

**Figure S25. The perspective association of SFTPD and chronic pain.**

**
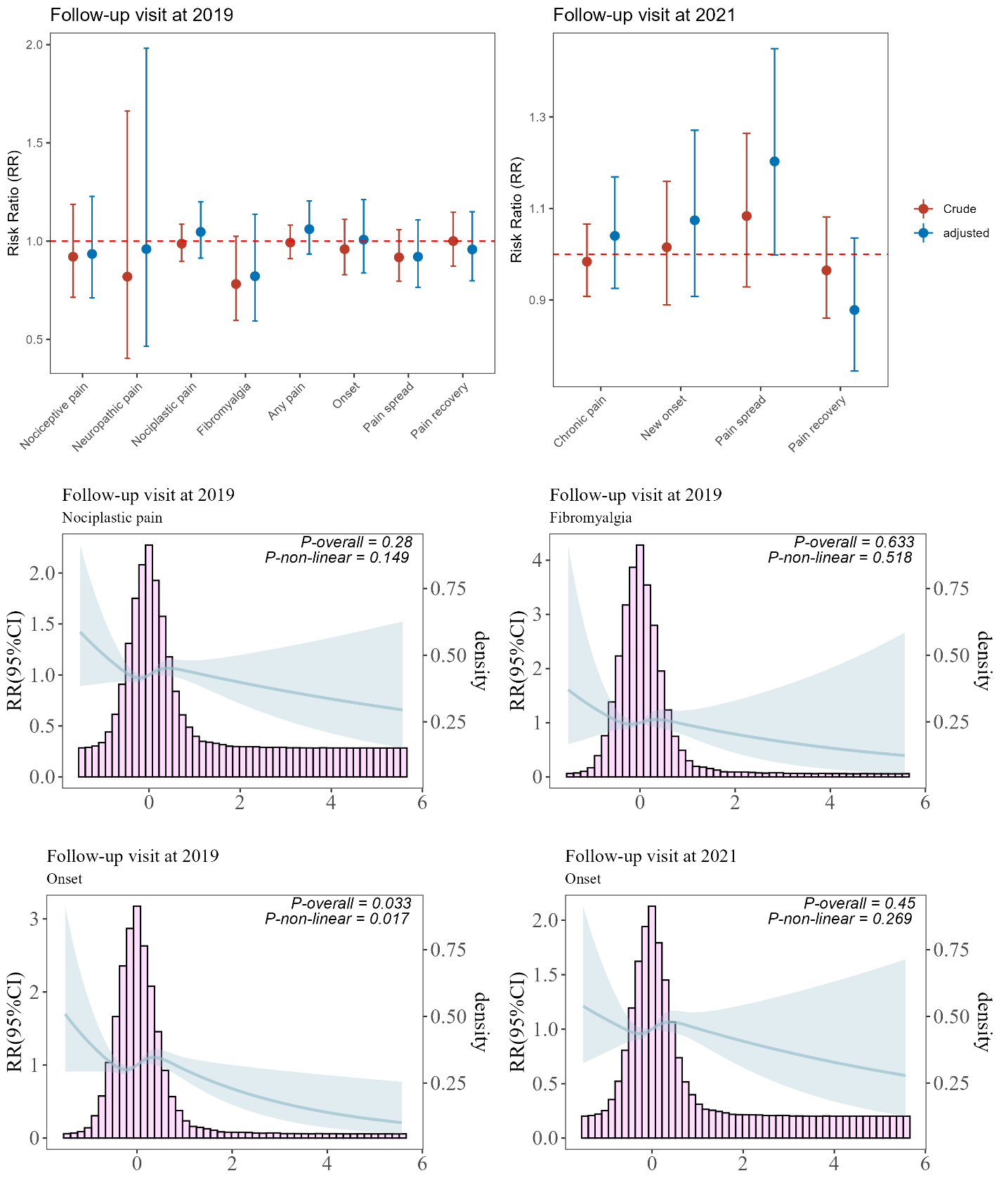
**

**Figure S26. The perspective association of ST3GAL1 and chronic pain.**

**
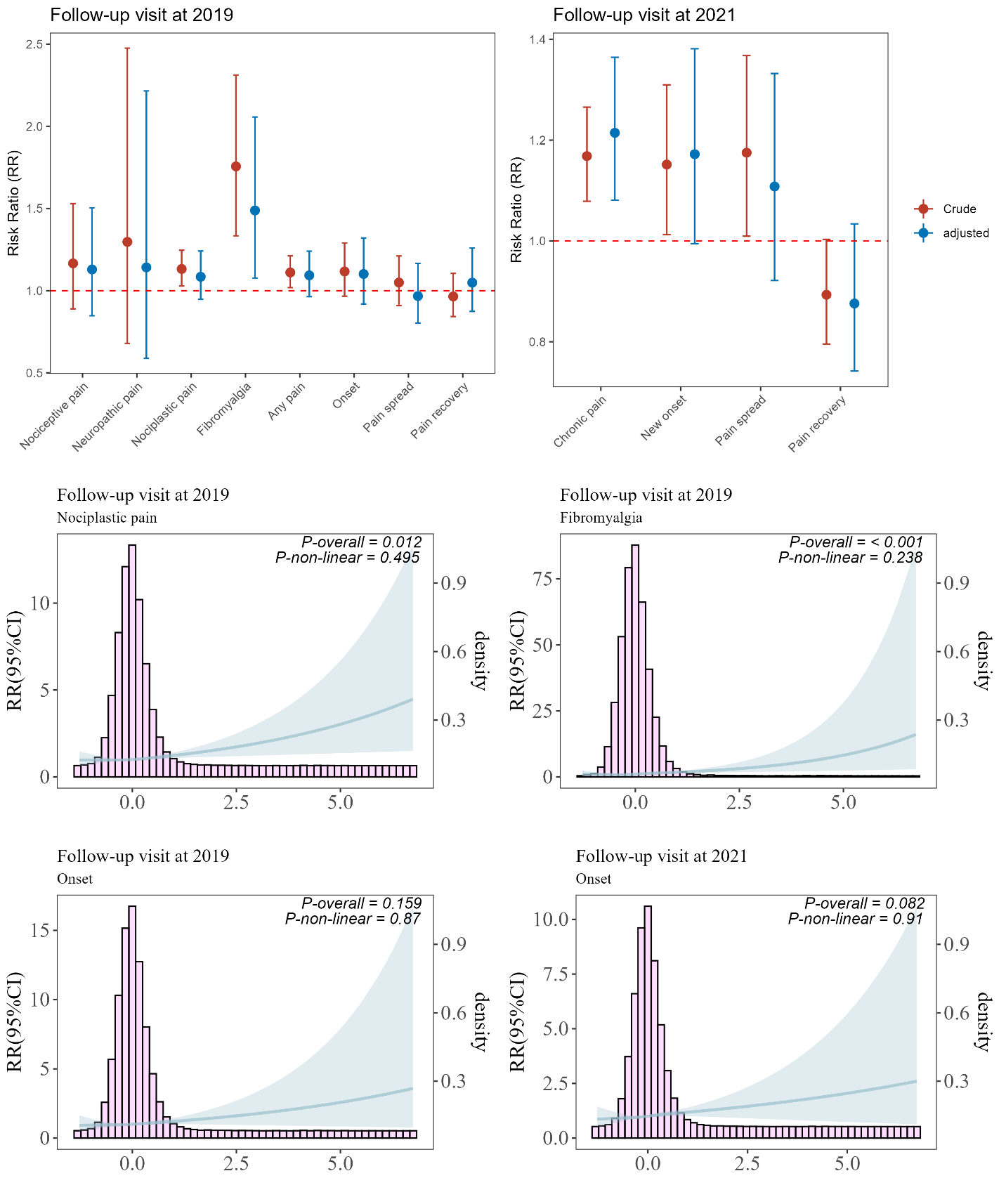
**

**Figure S27. The perspective association of TNF and chronic pain.**

**
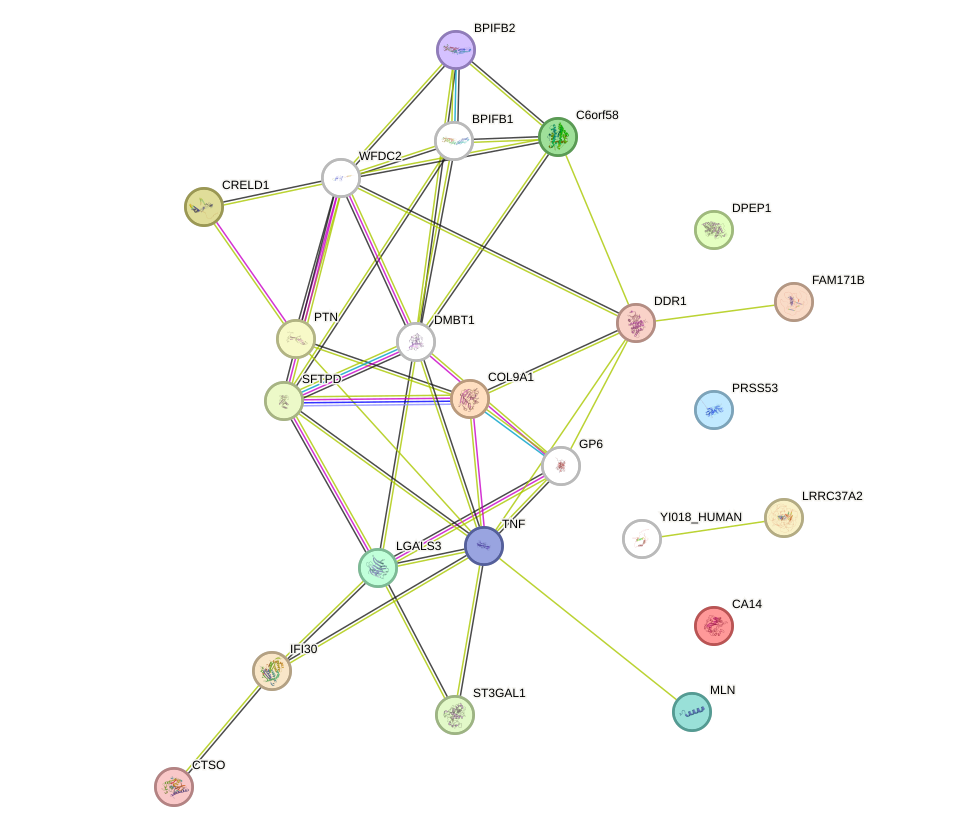
**

**Figure S28. protein-protein interaction.**


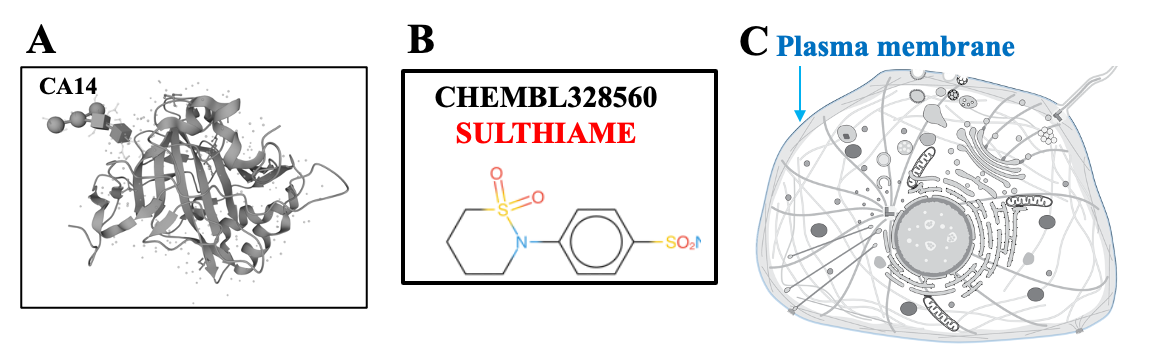


**Figure S29 Structural Interaction Between CA14 and SULTHIAME, Highlighting Its Target on the Plasma Membrane.**
